# Unexpected reactions of NHC*—Cu^I^ and —Ag^I^ bromides with potassium thio- or seleno­cyanate

**DOI:** 10.1107/S2056989019013719

**Published:** 2019-10-22

**Authors:** Matthias Tacke, Daniel Marhöfer, Hessah Althani, Helge Müller-Bunz

**Affiliations:** a School of Chemistry, University College Dublin, Belfield, Dublin 4, Ireland

**Keywords:** N-Heterocyclic carbene, copper(I), silver(I), thio­cyanate, seleno­cyanate, *d*^10^ electron configuration, crystal structure

## Abstract

The reactions of N-heterocyclic carbene Cu^I^ and Ag^I^ halides with potassium thio- or seleno­cyanate gave unexpected products. The attempted substitution reaction of bromido­(1,3-dibenzyl-4,5-di­phenyl­imidazol-2-yl­idene)silver (NHC*—Ag—Br) with KSCN yielded bis­[bis­(1,3-dibenzyl-4,5-di­phenyl­imidazol-2-yl­idene)silver(I)] tris­(thio­cyanato)­argentate(I) diethyl ether disolvate, [Ag(C_29_H_24_N_2_)_2_][Ag(NCS)_3_]·2C_4_H_10_O or [NHC*_2_Ag]_2_[Ag(SCN)_3_]·2Et_2_O, (**1**), while reaction with KSeCN led to bis­(μ-1,3-dibenzyl-4,5-diphenyl-2-seleno­imidazole-κ^2^ *Se*:*Se*)bis­[bromido­(1,3-dibenzyl-4,5-diphenyl-2-seleno­imid­azole-κ*Se*)silver(I)] di­chloro­methane hexa­solvate, [Ag_2_Br_2_(C_29_H_24_N_2_Se)_4_]·6CH_2_Cl_2_ or (NHC*Se)_4_Ag_2_Br_2_·6CH_2_Cl_2_, (**2**), *via* oxidation of the NHC* fragment to 2-seleno­imidazole. This oxidation was observed again in the reaction of NHC*—Cu—Br with KSeCN, yielding *catena*-poly[[[(1,3-dibenzyl-4,5-diphenyl-2-seleno­imidazole-κ*Se*)copper(I)]-μ-cyanido-κ^2^ *C*:*N*] aceto­nitrile monosolvate], {[Cu(CN)(C_29_H_24_N_2_Se)]·C_2_H_3_N}_*n*_ or NHC*Se—CuCN·CH_3_CN, (**3**). Compound (**1**) represents an organic/inorganic salt with Ag^I^ in a linear coordination in each of the two cations and in a trigonal coordination in the anion, accompanied by diethyl ether solvent mol­ecules. The tri-blade boomerang-shaped complex anion [Ag(SCN)_3_]^2−^ present in (**1**) is characterized by X-ray diffraction for the first time. Compound (**2**) comprises an isolated centrosymmetric mol­ecule with Ag^I^ in a distorted tetra­hedral BrSe_3_ coordination, together with di­chloro­methane solvent mol­ecules. Compound (**3**) exhibits a linear polymeric ^1^_∞_[Cu—C≡N—Cu—] chain structure with a seleno­imidazole moiety additionally coordinating to each Cu^I^ atom, and completed by aceto­nitrile solvent mol­ecules. Electron densities associated with an additional ether solvent mol­ecule in (**1**) and two additional di­chloro­methane solvent mol­ecules in (**2**) were removed with the SQUEEZE procedure [Spek (2015 ▸). *Acta Cryst.* C**71**, 9–18] in *PLATON*.

## Abstract 2

This article reports the unexpected reactions of Cu and Ag NHC bromides with potassium thio- or seleno­cyanates. It contains the first report of the boomerang-shaped [Ag(SCN)_3_]^2−^ ion.

## Chemical context   

**Figure 1:**
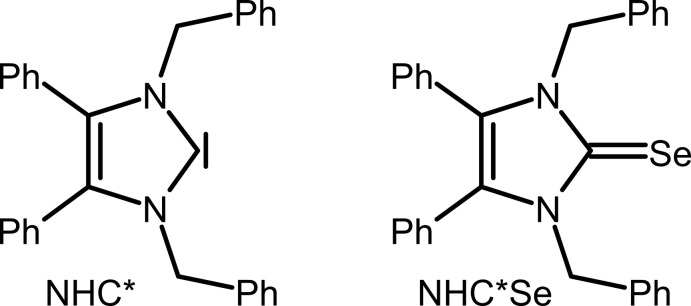
Lewis structures of the ligands NHC* and NHC*Se.

Copper and silver and their compounds exhibit fungicidal properties. For example, a copper di­hydroxide suspension in water – *The Bordeaux Mixture* – is a well-known early fungicide used in vineyards, while metallic silver has been used as an anti­microbial agent to purify drinking water for a long time. Alexander the Great stored drinking water in silver vessels during his military campaigns (White, 2002 ▸). Later, silver nitrate was used to treat wounds and infectious diseases even before the discovery of bacteria (Klasen, 2000 ▸). Silver sulfadiazine, discovered in the 1960s, was found to be more effective and safer than silver nitrate in treating burn wounds and is currently the most widely used remedy in burn centres (Fox, 1968 ▸). Silver is considered nontoxic to mammalian cells within the determined exposure limits of 0.01–0.1 mg m^−3^ (Drake & Hazelwood, 2005 ▸). Nevertheless, silver compounds may cause skin discoloration, known as Argyria (Kim *et al.*, 2009 ▸). The active species, the Ag^I^ cation, inhibits the respiratory path of sensitive strain organisms, destroys the cell wall, impairs essential enzymes, obstructs metabolic activity and/or causes RNA and DNA alteration (Silver, 2003 ▸; Starodub & Trevors, 1989 ▸). This topic saw renewed inter­est when Youngs and co-workers published the clean synthesis of N-heterocyclic (NHC) silver acetate derivatives from easily accessible imidazolium halides (Liang *et al.*, 2018 ▸); NHC–silver acetates exhibit good chemical stability through covalently bonded silver, which results in significant anti­biotic activity. Particularly well suited are complexes with benzyl-substituted ligands like 1,3-dibenzyl-4,5-di­phenyl­imidazol-2-yl­idene (NHC*; Fig. 1 ▸), *e.g.* NHC*–Ag–OAc (SBC3) (Patil *et al.*, 2011 ▸; Streciwilk *et al.*, 2014 ▸; Hackenberg & Tacke, 2014 ▸), which combine synthesis from a commercially available precursor (4,5-di­phenyl­imidazole) with high anti­microbial activity *in vitro* (Sharkey *et al.*, 2012 ▸) and *in vivo* (Browne *et al.* 2014 ▸). In the homologue gold series, the introduction of pseudohalide anions led to stable and bioactive compounds (Dada *et al.*, 2017 ▸). In this context, the attempted syntheses of the analogous NHC*—Ag^I^ and —Cu^I^ thio- and seleno­cyanates as potential anti­microbial species led to unexpected results with the formation of bis­[bis­(1,3-dibenzyl-4,5-di­phenyl­imidazol-2-yl­idene)silver(I)] tris­(thio­cyanato)­argentate(I) diethyl ether disolvate, (**1**), bis­(μ-1,3-dibenzyl-4,5-diphenyl-2-seleno­imid­azole-κ^2^ *Se*:*Se*)bis­[bromido­(1,3-dibenzyl-4,5-diphenyl-2-seleno­imidazole-κ*Se*)silver(I)] di­chloro­methane hexa­solvate, (**2**), and *catena*-poly[[[(1,3-dibenzyl-4,5-diphenyl-2-seleno­imidazole-κ*Se*)copper(I)]-μ-cyanido-κ^2^ *C*:*N*] aceto­nitrile monosolvate], {[Cu(CN)(C_29_H_24_N_2_Se)]·C_2_H_3_N}_*n*_ or NHC*Se—CuCN·CH_3_CN, (**3**), the crystal structures of which are reported in this communication.
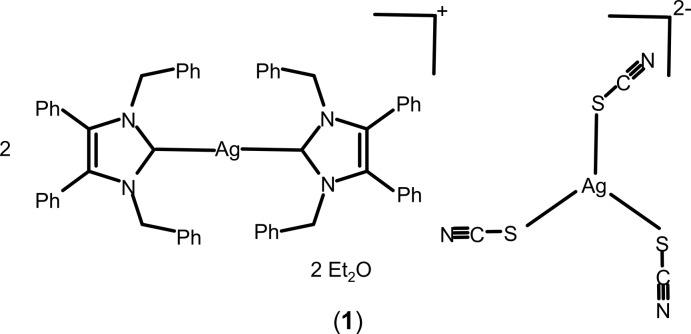
 
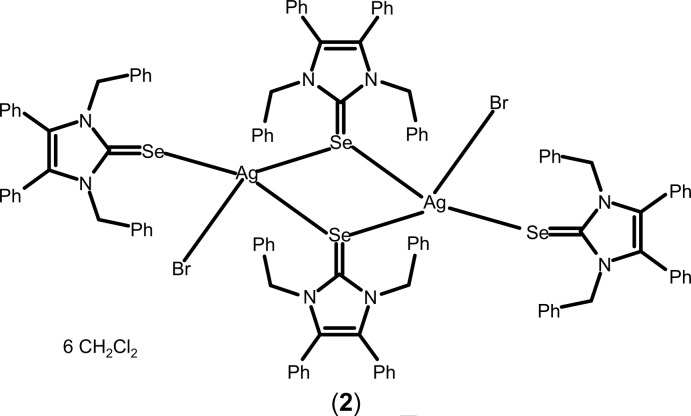


## Structural commentary   

### [NHC*_2_Ag]_2_[Ag(SCN)_3_]·2Et_2_O, (1)   

**Figure 2:**
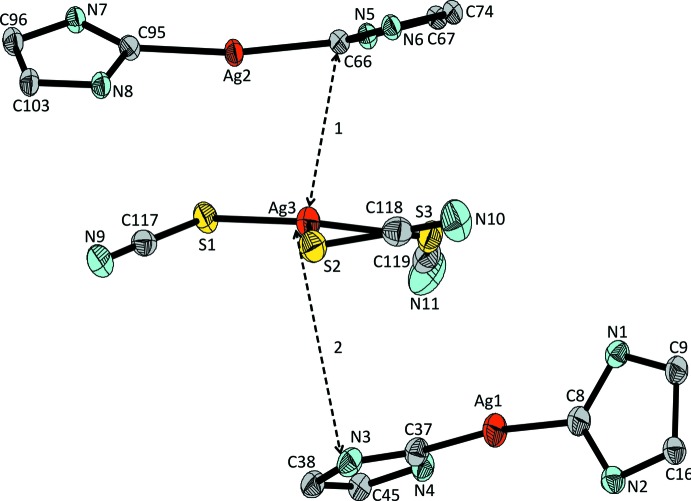
The [Ag(SCN)_3_]^2−^ anion in (**1**) with the two closest [Ag(NHC*)_2_]^+^ cations. Substituents on the imidazole moiety have been omitted for clarity and displacement ellipsoids are drawn at the 50% probability level.

**Figure 3:**
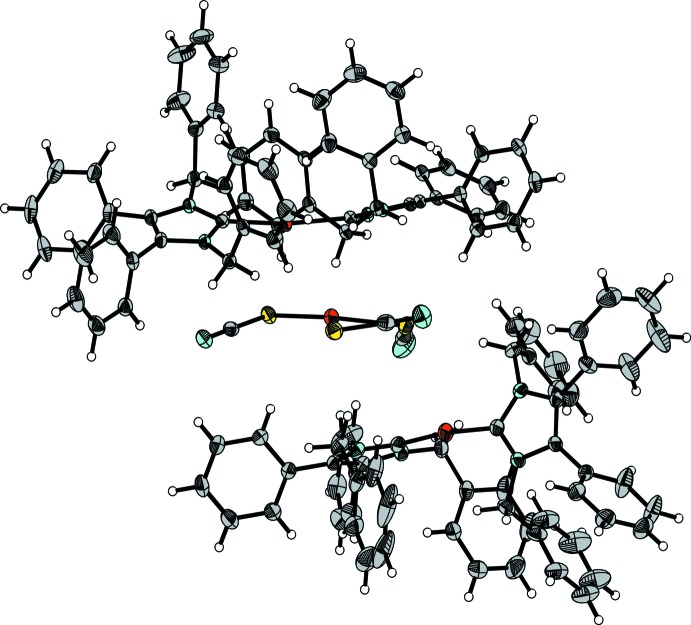
The [Ag(SCN)_3_]^2−^ anion in (**1**) with the two closest [Ag(NHC*)_2_]^+^ cations. Displacement ellipsoids are drawn at the 50% probability level.

Both of the crystallographically distinct [NHC*_2_Ag]^+^ cations (Ag1 and Ag2) in the organic/inorganic salt show a nearly linear C—Ag—C angle and two almost identical Ag—C bond lengths (Table 1 ▸). In between these two bulky cations is located one [Ag(SCN)_3_]^2−^ anion with a rare coordination number of three for monovalent silver(I) (Ag3). The corresponding Ag—S bond lengths cover the range 2.4657 (5)–2.5377 (6) Å. The flexibility of the [Ag(SCN)_3_]^2−^ anion also shows itself in the bond angles, with Ag—S—C angles ranging from 93.45 (8) to 105.56 (9)°, and S—Ag—S angles ranging from 104.96 (2) to 127.68 (2)° (Table 1 ▸). As expected, all three SCN^−^ ligands are virtually linear. The sum of the S—Ag—S bond angles (Table 1 ▸) indicates that the anion is almost planar [the deviation of the Ag3 from the least-squares plane of the three S atoms is 0.1270 (5) Å]. The [Ag(SCN)_3_]^2−^ anion is situated between the two crystallographically independent cations, but not in the middle (Fig. 2 ▸): cation 1 (Ag1) has a shortest distance of 4.161 (2) Å from N3 to Ag3 (line 2 in Fig. 2 ▸), whereas cation 2 (Ag2) has a shortest distance of 3.069 (2) Å from C66 to Ag3 (line 1 in Fig. 2 ▸). As a consequence of this close association, the benzyl groups in cation 2 are all aligned away from the anion. Due to its greater distance from the anion, the benzyl groups of cation 1 have greater flexibility, allowing it to take a shape suitable to fill gaps in the packing caused by the constraint on cation 2 (Fig. 3 ▸). The remaining gaps are filled by two noncoordinating diethyl ether mol­ecules, one of which is highly disordered and could not be refined in terms of atomic sites. The SQUEEZE option (Spek, 2015 ▸) in *PLATON* was used to compensate for the ill-defined electron density.
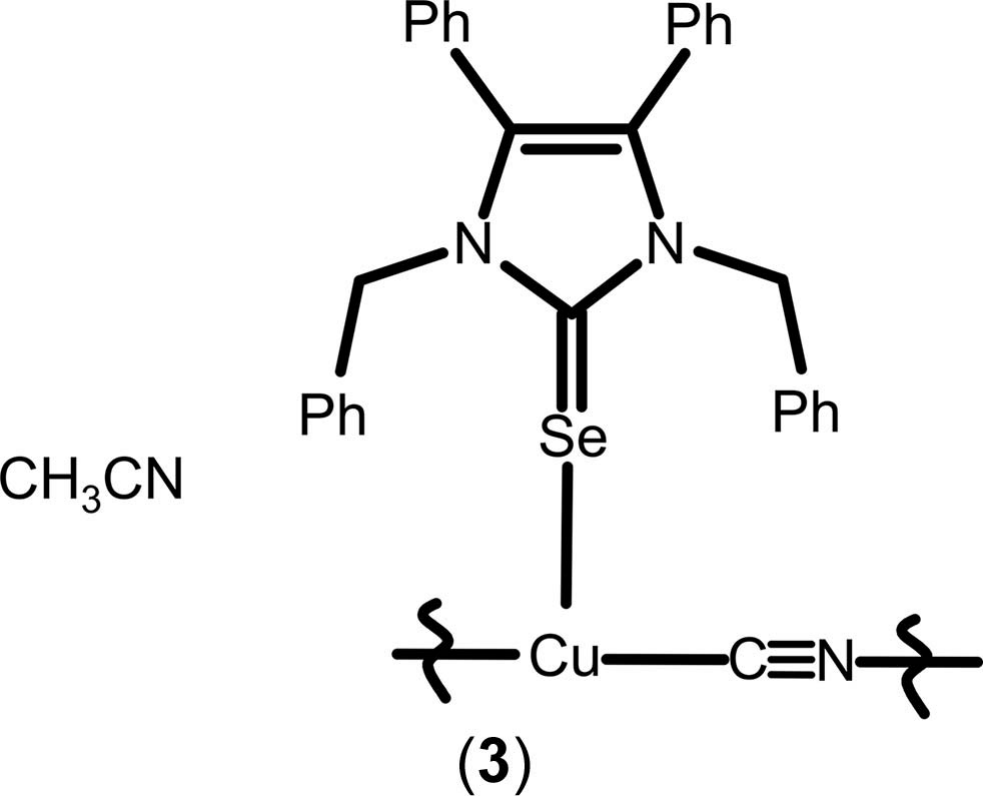


### (NHC*Se)_4_Ag_2_Br_2_·6CH_2_Cl_2_, (2)   

**Figure 4:**
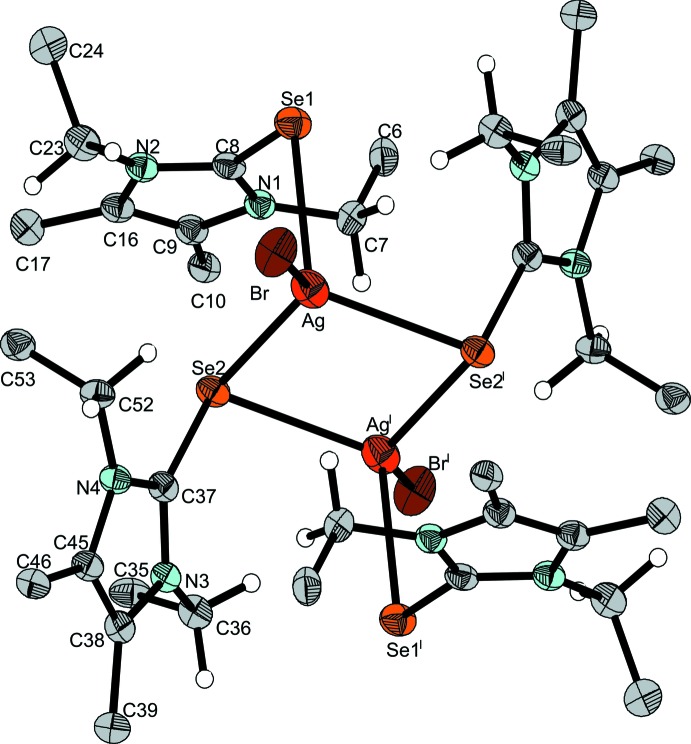
The mol­ecular structure of (**2**), with phenyl groups represented by their *ipso* carbon atoms only. Displacement ellipsoids are drawn at the 50% probability level. [Symmetry code: (I) −*x* + 1, −*y* + 1, −*z* + 1.]

Compound (**2**) is characterized by a mol­ecular structure complemented by di­chloro­methane solvent mol­ecules. Two Ag^I^ cations and two bridging NHC*Se ligands build up a centrosymmetric four-membered Ag_2_Se_2_ ring. Each silver cation carries a further terminal NHC*Se ligand and a terminal bromide ligand, in each case leading to a coordination number of 4 in the shape of a distorted tetra­hedron (Table 1 ▸ and Fig. 4 ▸). One of the bridging Ag—Se distances is 2.7677 (4) Å, significantly longer than the other [2.7187 (4) Å] or the terminal one [2.6899 (4) Å], suggesting that two AgBr(NHC*Se)_2_ moieties are weakly attached to each other. The Ag—Se—C angles are all strongly bent (Table 1 ▸), as one would expect. The bridging and terminal NHC*Se ligand pairs, as well as the two bromide ligands, end up in pseudo-*trans* positions with respect to each other, allowing an overall compact shape of the uncharged [AgBr(NHC*Se)_2_]_2_ complex. Gaps in the packing are filled by di­chloro­methane solvent mol­ecules, two of which were treated with the SQUEEZE option in *PLATON*.

### NHC*Se—CuCN·CH_3_CN, (3)   

**Figure 5:**
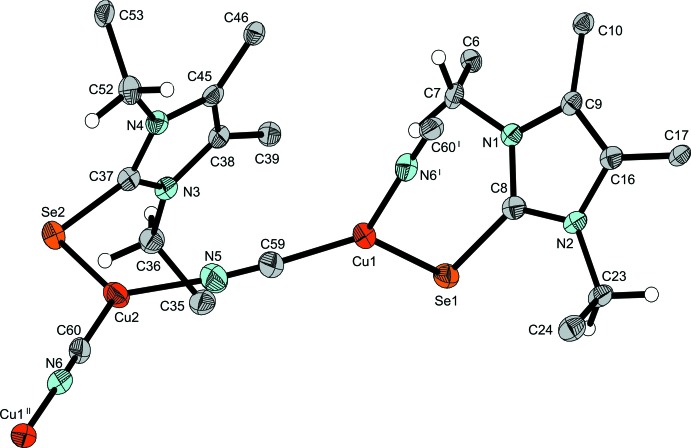
Section of the polymeric structure of (**3**), with displacement ellipsoids drawn at the 50% probability level. Phenyl groups are represented by their *ipso* carbons only and aceto­nitrile solvent mol­ecules have been omitted for clarity. [Symmetry codes: (I) −*x* + 2, *y* + 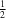, −*z* + 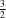; (II) −*x* + 2, *y* − 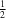, −*z* + 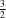.]

The structure of (**3**) is polymeric in nature and contains two distinct Cu^I^ atoms. The backbone of the structure is a linear copper–cyanide polymer ^1^_∞_[Cu1—C≡N—Cu2—], where every Cu^I^ atom is also coordinated by selenium from a terminal NHC*Se ligand (Fig. 5 ▸). The Cu—Se—C angles are in the same region as those in (**2**) (Table 1 ▸). The carbon and nitro­gen atoms of the two cyanide anions can be distinguished, not only by their electron densities, but also by their different bond lengths to Cu^I^ atoms, with Cu—N shorter by ≃ 0.04 Å (Table 1 ▸). The relatively bulky NHC*Se ligands, which lead to the rare coordination number of 3 of the two Cu^I^ cations, move to opposite positions with respect to the copper cyanide polymer, allowing better packing for the overall structure (Fig. 5 ▸). The sum of the three angles at Cu1 and Cu2 (Table 1 ▸) indicate that the coordination is practically planar at the central copper(I) atoms (the displacement of Cu1 from the least-squares Se1/N6/C5 plane is 0.099 Å and of Cu2 from the least-squares Se2/N5/C60 plane is 0.054 Å). Aceto­nitrile solvent mol­ecules fill voids in the crystal packing.

All reactions reported here include cleavage of an Ag– or Cu–carbene bond, suggesting that even at room temperature (Cu) or in refluxing di­chloro­methane (Ag) the targeted NHC*—*M*—SeCN complexes are not very stable in solution, but are liable to Schlenk-type equilibrium exchange. It is worth mentioning that the syntheses of (**2**) and (**3**) require SeCN^−^ acting as a selenating agent similar to selenium powder (Verlinden *et al.*, 2015 ▸) or Woollins Reagent (Bockfeld *et al.*, 2017 ▸), even under the relatively mild conditions reported here. Thus, neither thio­cyanate nor seleno­cyanate take up their roles as unreactive substituents in these planned substitution reactions. These findings raise questions about the suitability of NHC—*M*—*X* (*M* = Cu and Ag; *X* = pseudohalide) as drugs because drug mol­ecules need to be stable in solution under ambient conditions.

## Supra­molecular features   

**Figure 6:**
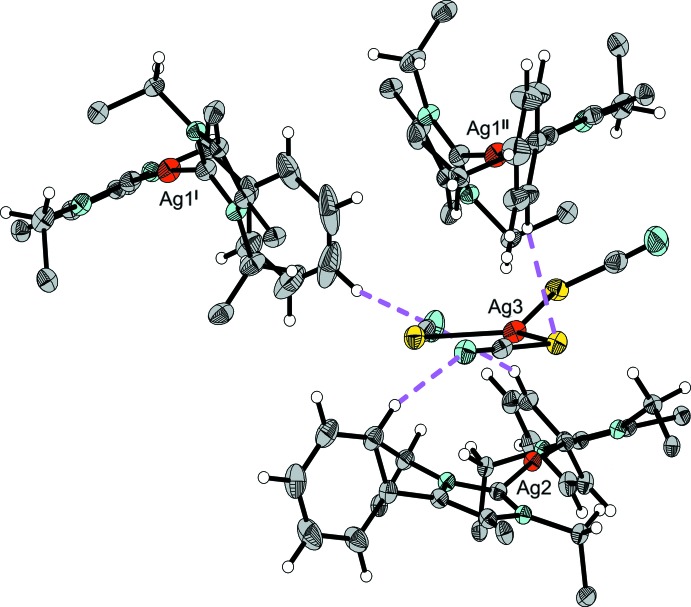
The [Ag(SCN)_3_]^2−^ anion in (**1**) involved in nonclassical hydrogen-bonding inter­actions, shown as pink dashed lines. Phenyl groups are represented by their *ipso* carbons only and displacement ellipsoids are drawn at the 50% probability level. [Symmetry codes: (I) −*x* + 2, −*y* + 2, −*z* + 1; (II) *x*, *y*, *z* + 1.]

**Figure 7:**
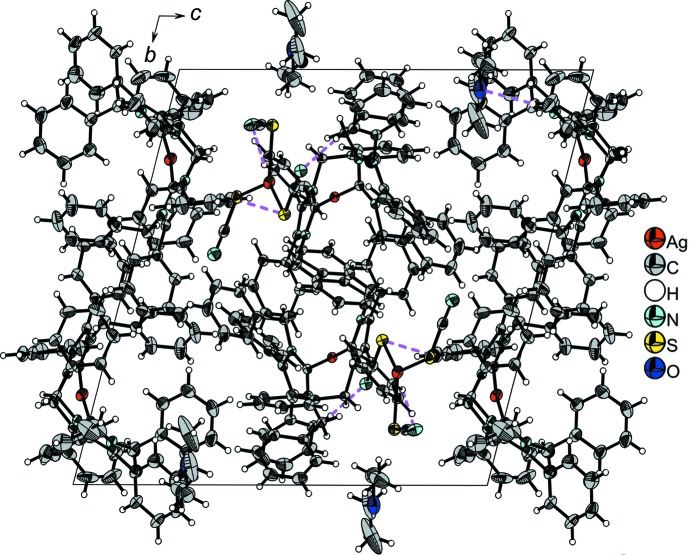
View of the crystal structure of (**1**) along [100]. Displacement ellipsoids are drawn at the 50% probability level and hydrogen-bonding inter­actions are shown as pink dashed lines.

In (**1**), there are some weak nonclassical hydrogen bonds between the cations and the solvent mol­ecules, as detailed in Table 2 ▸. One anion has connections to the two cations closest to it, as described above, and one to the cation of an adjacent ion triplet (Fig. 6 ▸), linking the ion triplets into a one-dimensional chain. Fig. 7 ▸ shows a view of (**1**) along [100] with these contacts shown as dashed lines.

All nonclassical hydrogen bonds in (**2**) and (**3**) are intra­molecular, and we are not aware of any other noteworthy inter­molecular features in these structures.

## Database survey   

To the best of our knowledge, the crystal structure of (**1**) is the first report of a salt with the tri-blade boomerang-shaped [Ag(SCN)_3_]^2−^ ion. The alkaline metals salts Rb_2_Ag(SCN)_3_ and Rb_2_Ag(SCN)_3_ have one-dimensional polymeric chains as anions rather than isolated [Ag(SCN)_3_]^2−^ (Thiele & Kehr, 1984 ▸). Hathaway *et al.* (1970 ▸) reported the spectroscopic properties of [Cu(NH_3_)_2_Ag(SCN)_3_] and indicated that they had determined its crystal structure as well. However, in this article, only the space group type (*P* 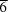
2*c*) and the number of formula units (*Z* = 2) were given, not the crystal structure itself. From what is reported it can be gleaned that the anion must be situated on a 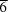 position, *i.e.* it is planar and adhering to threefold rotation symmetry. If this is true then this is in stark contrast to the [Ag(SCN)_3_]^2−^ anion reported here, where the Ag—S bond lengths and S—Ag—S and Ag—S—C bond angles cover a wide range. However, since together with the information above only a schematic drawing of the surrounding of the Cu^II^ atom was given, we cannot establish structural details of the anion in [Cu(NH_3_)_2_Ag(SCN)_3_] with any degree of certainty.

The Ag—Se distances in (**2**) fall well within the region reported for similar compounds (Perras *et al.*, 2018 ▸; Nahra *et al.*, 2018 ▸). Remarkably, at least for the neutral compounds, the distances do not depend on whether the coordination number around the silver is 3 or 4: In *N*,*N*-dimesityl­seleno­imidazole–silver nitrate, the Ag—Se bond lengths range from 2.65 to 2.68 Å for the four-coordinate atom and from 2.63 to 2.71 for the three-coordinate atom (Perras *et al.*, 2018 ▸).

A comparison with the compounds reported by Kimani *et al.* (2011 ▸) shows a remarkable impact of the cyanide anion on the Cu—Se bond length compared with the corresponding halides. For threefold-coordinated Cu, the distances between Cu and nonbridging Se range from 2.33 to 2.35 Å, whereas both of them in (**3**) are about 2.39 Å (Table 1 ▸). This is closer to the Cu—μ-Se distances (2.41–2.42 Å) reported by Kimani *et al.* (2011 ▸). In other words, cyanide is the better ligand for Cu^I^ when compared with halides, and as a result relatively long Cu—Se distances are observed for cyanide derivative (**3**).

## Synthesis and crystallization   

### [NHC*_2_Ag]_2_[Ag(SCN)_3_]·2Et_2_O, (1)   

1,3-Dibenzyl-4,5-di­phenyl­imidazolium bromide (481 mg, 1.00 mmol), silver oxide (116 mg, 0.500 mmol) and potassium thio­cyanate (107 mg, 1.10 mmol) were suspended in di­chloro­methane (35 ml). After stirring for 20 h under reflux, the solution was filtered and the volume of the solvent was reduced to approximately 5 ml. Pentane (40 ml) was then added and a colourless solid precipitated. The product was filtered off, washed with pentane and dried *in vacuo* (yield: 366 mg, 0.647 mmol, 65%) as a colourless powder. ^1^H NMR (300 MHz, CDCl_3_): δ 1.60 (*s*, 2H, H_2_O), 5.49 (*s*, 4H, C_aliph_—H), 6.90–7.43 (*m*, 20H, C_ar_—H). IR (ATR, cm^−1^): 3028 [ν(CH_ar_)], 2924 [ν(CH_aliph_)], 2053 [ν(S—C≡N)], 1445, 1348, 1021, 764, 731, 696. Elemental analysis calculated (%): C 63.61, H 4.27, N 7.42; found: C 64.52, H 4.69, N 6.64.

Diethyl ether was diffused into a saturated solution of the crude product in THF; from this solution, that was kept for 10 d at 277 K, the title compound [NHC*_2_Ag]_2_[Ag(SCN)_3_] crystallized in the form of needles of the diethyl ether disolvate.

### (NHC*Se)_4_Ag_2_Br_2_·6CH_2_Cl_2_, (2)   

1,3-Dibenzyl-4,5-di­phenyl­imidazolium bromide (481 mg, 1.00 mmol), silver oxide (116 mg, 0.500 mmol) and potassium seleno­cyanate (159 mg, 1.10 mmol) were suspended in 35 ml of di­chloro­methane. After stirring for 20 h under reflux, the solution was filtered and the volume of the solvent was reduced to approximately 5 ml. Then 40 ml of pentane were added and a yellow solid precipitated. The product was filtered off, washed with pentane and dried *in vacuo* (yield: 365 mg, 0.159 mmol, 64%) as a yellow powder. ^1^H NMR (300 MHz, CDCl_3_): δ 5.23–5.57 (*m*, 16H, C_aliph_—H), 6.77–7.43 (*m*, 80H, C_ar_—H). IR (ATR, cm^−1^): 3029 [ν(CH_ar_)], 2926 [ν(CH_aliph_)], 1447, 1402, 764, 732, 696, 520. Elemental analysis calculated (%): C 59.08, H 4.15, N 4.71; found: C 58.39, H 3.86, N 5.08.

A saturated solution of the compound in di­chloro­methane was prepared at 313 K; from this solution, that was kept for 7 d at 253 K, the title compound (NHC*Se)_4_Ag_2_Br_2_ crystallized as clear pale-yellow block-like prisms of the di­chloro­methane hexa­solvate.

### NHC*Se—CuCN·CH_3_CN, (3)   

(1,3-Dibenzyl-4,5-di­phenyl­imidazol-2-yl­idene)copper(I) bro­mide (270 mg, 0.50 mmol) and potassium seleno­cyanate (72 mg, 0.50 mmol) were suspended in 15 ml of di­chloro­methane followed by 5 ml of water. After stirring for 16 h at room temperature (*ca* 295K) under nitro­gen, the solutions were filtered and the two phases separated. The aqueous phase was washed with di­chloro­methane (2 × 10 ml) and the organic phase was washed with deionized water (2 × 10 ml). The organic phases were combined and dried over magnesium sulfate. The volume of the solvent was reduced to approximately 5 ml before 20 ml of pentane were added and a colourless solid precipitated. The product was filtered off, washed with pentane and dried *in vacuo* (yield: 121 mg, 0.212 mmol, 42%) as a colourless powder. ^1^H NMR (300MHz, CDCl_3_): δ 7.29–6.97 (*m*, 20H, CH_arom_), 5.50 (*s*, 4H, CH_2benz­yl_). IR (ATR, cm^−1^): 3048 (*w*), 2105 (*s*) (ν_CN_), 1445 (*m*, *v*), 695 (*s*). M.p. 368 K. Elemental analysis calculated (%) for C_30_H_25_N_3_CuSe: C 63.21, H 4.97, N 7.37; found: C 61.68, H 4.06, N 7.11.

Pentane was diffused into a saturated solution of the crude product in di­chloro­methane/aceto­nitrile 1:1 (*v*:*v*). From this solution, kept for 10 d at 277 K, the title compound [(NHC*Se)CuCN]_∞_ crystallized as needles of the aceto­nitrile solvate.

## Refinement details   

Crystal data, data collection and structure refinement details are summarized in Table 3 ▸. H atoms were placed at calculated positions and treated as riders, with *U*_iso_ values set at 1.2*U*_eq_ or 1.5*U*_eq_ of their respective bonding partners.

In the crystal structure of (**1**), one of the phenyl groups was refined as partially disordered over two positions rotated against each other around the *ipso*–*para* axis [occupancy ratio 0.55 (2):0.45 (2)]. In the crystal structure of (**2**), one of the dichoro­methane solvent mol­ecules was refined over two positions [ratio 0.898 (4):0.102 (4)] due to positional disorder around one C—Cl bond.

The SQUEEZE procedure (Spek, 2015 ▸) in *PLATON* was used to treat regions of disordered solvent mol­ecules in (**1**) and (**2**) which could not be modelled in terms of atomic sites. In (**1**), the number of electrons found in these regions in the unit cell, 82, was assigned to two diethyl ether solvent mol­ecules (ideal 84 electrons). In (**2**), 84 electrons were found and assigned to two solvent mol­ecules of di­chloro­methane in the unit cell (ideal 84 electrons). Since *Z* = 2 for (**1**) and *Z* = 1 for (**2**), one solvent mol­ecule of diethyl ether in (**1**) and two solvent mol­ecules of di­chloro­methane in (**2**) are missing in the final models and the given chemical formulae and other crystal data given in Table 3 ▸ take into account these solvent mol­ecules.

## Supplementary Material

Crystal structure: contains datablock(s) 1, 2, 3, global. DOI: 10.1107/S2056989019013719/wm5519sup1.cif

Structure factors: contains datablock(s) 1. DOI: 10.1107/S2056989019013719/wm55191sup2.hkl

Structure factors: contains datablock(s) 2. DOI: 10.1107/S2056989019013719/wm55192sup3.hkl

Structure factors: contains datablock(s) 3. DOI: 10.1107/S2056989019013719/wm55193sup4.hkl

CCDC references: 1958163, 1958164, 1958165, 1958163, 1958164, 1958165

Additional supporting information:  crystallographic information; 3D view; checkCIF report

## Figures and Tables

**Table 1:** Selected inter­nuclear distances and bond angles (Å, °) for (**1**), (**2**) and (**3**)

Atoms	Distance	Atoms	Angle
(**1**)			
Ag1—C8	2.091 (2)	S1—Ag3—S2	127.68 (2)
Ag1—C37	2.085 (2)	S2—Ag3—S3	103.96 (2)
Ag2—C66	2.097 (2)	S3—Ag3—S1	126.94 (2)
Ag2—C95	2.102 (2)	Sum	358.58
Ag3—S1	2.4657 (5)	C8—Ag1—C37	173.06 (8)
Ag3—S2	2.5377 (6)	C66—Ag2—C95	172.01 (7)
Ag3—S3	2.4940 (6)	Ag3—S —C117	100.90 (7)
		Ag3—S2—C118	93.45 (8)
Ag3⋯N3	4.161 (2)	Ag3—S3—C119	105.56 (9)
Ag3⋯C66	3.069 (2)	S1—C117—N9	177.8 (2)
		S2—C118—N10	179.4 (3)
		S3—C119—N11	177.1 (2)
			
(**2**)			
Ag—Se1	2.6899 (4)	Br—Ag—Se1	102.274 (13)
Ag—Se2	2.7677 (4)	Br—Ag—Se2	109.628 (12)
Ag—Se2#1	2.7187 (4)	Br—Ag—Se2#1	126.883 (14)
Ag—Br	2.6631 (4)	Se1—Ag—Se2	110.623 (12)
		Se1—Ag—Se2#1	100.026 (11)
		Se2—Ag—Se2#1	106.352 (11)
		Ag—Se1—C8	94.72 (8)
		Ag—Se2—C37	100.72 (8)
		Ag#1—Se2—C37	108.43 (8)
		Ag—Se2—Ag#1	73.649 (11)
			
(**3**)			
Cu1—Se1	2.3900 (6)	Se1—Cu1—C59	125.06 (12)
Cu1—C59	1.898 (4)	Se1—Cu1—N6#2	110.25 (9)
Cu1—N6#2	1.939 (3)	C59—Cu1—N6#2	124.68 (14)
		Sum	359.99
Cu2—Se2	2.3861 (6)	Se2—Cu2—C60	126.88 (11)
Cu2—C60	1.895 (4)	Se2—Cu2—N5	110.28 (10)
Cu2—N5	1.937 (3)	C60—Cu2—N5	122.84 (15)
		Sum	360.00
		Cu1—Se1—C8	95.96 (10)
		Cu2—Se2—C37	93.84 (11)

Symmetry codes: (#1) −*x* + 1, −*y* + 1, −*z* + 2; (#2) −*x* + 2, *y* + 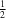, −*z* + 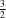.

**Table 2:** Hydrogen-bond geometry (Å, °) for (**1**)

*D*—H⋯*A*	*D*—H	H⋯*A*	*D*⋯*A*	*D*—H⋯*A*
C31—H31⋯O1	0.95	2.57	3.361 (4)	141
C32—H32⋯N10^i^	0.95	2.43	3.327 (4)	157
C40—H40⋯S1^ii^	0.95	2.82	3.684 (2)	152

Symmetry codes: (i) 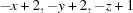; (ii) 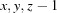.

**Table 3:** Experimental details

	(1)	(2)	(3)
Crystal data			
Chemical formula	[Ag(C_29_H_24_N_2_)_2_][Ag(NCS)_3_]·2C_4_H_10_O	[Ag_2_Br_2_(C_29_H_24_N_2_Se)_4_]·6CH_2_Cl_2_	[Cu(CN)(C_29_H_24_N_2_Se)]·C_2_H_3_N
*M*_r_	2248.09	2802.96	610.07
Crystal system, space group	Triclinic, *P* 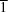	Triclinic, *P* 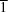	Monoclinic, *P*2_1_/*c*
Temperature (K)	100	150	100
*a*, *b*, *c* (Å)	14.86462 (8), 19.3714 (1), 19.85451 (9)	13.6265 (1), 14.7422 (1), 16.9397 (2)	13.7704 (3), 14.3398 (3), 28.4102 (7)
α, β, γ (°)	102.7710 (4), 100.8268 (4), 99.5778 (4)	106.4172 (7), 112.2820 (8), 96.2211 (6)	90, 93.024 (2), 90
*V* (Å^3^)	5345.84 (5)	2930.04 (5)	5602.2 (2)
*Z*	2	1	8
Radiation type	Cu *K*α	Mo *K*α	Mo *K*α
μ (mm^−1^)	5.37	2.58	2.11
Crystal size (mm)	0.32 × 0.06 × 0.04	0.37 × 0.26 × 0.20	0.35 × 0.12 × 0.11

Data collection			
Diffractometer	Rigaku SuperNova, Dual, Cu at zero, Atlas	Rigaku SuperNova, Dual, Cu at zero, Atlas	Rigaku SuperNova, Dual, Cu at zero, Atlas
Absorption correction	Gaussian (*CrysAlis PRO*; Rigaku OD, 2015 ▸)	Gaussian (*CrysAlis PRO*; Rigaku OD, 2015 ▸)	Gaussian (*CrysAlis PRO*; Rigaku OD, 2015 ▸)
*T*_min_, *T*_max_	0.437, 0.848	0.517, 0.664	0.643, 0.851
No. of measured, independent and observed [*I* > 2σ(*I*)] reflections	140384, 22376, 19576	119415, 12002, 10726	71725, 11392, 10122
*R*_int_	0.045	0.037	0.038
(sin θ/λ)_max_ (Å^−1^)	0.632	0.626	0.626

Refinement			
*R*[*F* ^2^ > 2σ(*F* ^2^)], *wR*(*F* ^2^), *S*	0.030, 0.080, 1.03	0.035, 0.096, 1.03	0.048, 0.119, 1.06
No. of reflections	22376	12002	11392
No. of parameters	1309	654	687
H-atom treatment	H-atom parameters constrained	H-atom parameters constrained	H-atom parameters constrained
Δρ_max_, Δρ_min_ (e Å^−3^)	0.98, −1.13	1.05, −1.49	2.04, −0.56

Computer programs: *CrysAlis PRO* (Rigaku OD, 2015 ▸), *SHELXT* (Sheldrick, 2015*a* ▸), *SHELXL2014* (Sheldrick, 2015*b* ▸), *DIAMOND* (Brandenburg, 1999 ▸) and *publCIF* (Westrip, 2010 ▸).

## supplementary crystallographic information

### Bis[bis(1,3-dibenzyl-4,5-diphenylimidazol-2-ylidene)silver(I)] tris(thiocyanato)argentate(I) diethyl ether disolvate (1) Crystal data

**Table:**

[Ag(C_29_H_24_N_2_)_2_][Ag(NCS)_3_]·2C_4_H_10_O	*Z* = 2
*M**_r_* = 2248.09	*F*(000) = 2320
Triclinic, *P*1	*D*_x_ = 1.397 Mg m^−^^3^
*a* = 14.86462 (8) Å	Cu *K*α radiation, λ = 1.54184 Å
*b* = 19.3714 (1) Å	Cell parameters from 59132 reflections
*c* = 19.85451 (9) Å	θ = 3.7–76.7°
α = 102.7710 (4)°	µ = 5.37 mm^−^^1^
β = 100.8268 (4)°	*T* = 100 K
γ = 99.5778 (4)°	Needle, colourless
*V* = 5345.84 (5) Å^3^	0.32 × 0.06 × 0.04 mm

### Bis[bis(1,3-dibenzyl-4,5-diphenylimidazol-2-ylidene)silver(I)] tris(thiocyanato)argentate(I) diethyl ether disolvate (1) Data collection

**Table:**

Rigaku SuperNova, Dual, Cu at zero, Atlas diffractometer	22376 independent reflections
Radiation source: micro-focus sealed X-ray tube	19576 reflections with *I* > 2σ(*I*)
Detector resolution: 10.3196 pixels mm^-1^	*R*_int_ = 0.045
ω scans	θ_max_ = 76.9°, θ_min_ = 3.4°
Absorption correction: gaussian (CrysAlis PRO; Rigaku OD, 2015)	*h* = −18→17
*T*_min_ = 0.437, *T*_max_ = 0.848	*k* = −24→24
140384 measured reflections	*l* = −24→25

### Bis[bis(1,3-dibenzyl-4,5-diphenylimidazol-2-ylidene)silver(I)] tris(thiocyanato)argentate(I) diethyl ether disolvate (1) Refinement

**Table:**

Refinement on *F*^2^	Primary atom site location: heavy-atom method
Least-squares matrix: full	Secondary atom site location: other
*R*[*F*^2^ > 2σ(*F*^2^)] = 0.030	Hydrogen site location: inferred from neighbouring sites
*wR*(*F*^2^) = 0.080	H-atom parameters constrained
*S* = 1.03	*w* = 1/[σ^2^(*F*_o_^2^) + (0.0438*P*)^2^ + 3.189*P*] where *P* = (*F*_o_^2^ + 2*F*_c_^2^)/3
22376 reflections	(Δ/σ)_max_ = 0.003
1309 parameters	Δρ_max_ = 0.98 e Å^−^^3^
0 restraints	Δρ_min_ = −1.13 e Å^−^^3^

### Bis[bis(1,3-dibenzyl-4,5-diphenylimidazol-2-ylidene)silver(I)] tris(thiocyanato)argentate(I) diethyl ether disolvate (1) Special details

**Table:**

Geometry. All esds (except the esd in the dihedral angle between two l.s. planes) are estimated using the full covariance matrix. The cell esds are taken into account individually in the estimation of esds in distances, angles and torsion angles; correlations between esds in cell parameters are only used when they are defined by crystal symmetry. An approximate (isotropic) treatment of cell esds is used for estimating esds involving l.s. planes.
Refinement. The structure was first solved by the Patterson method as implemented in SHELXS. The three silver atoms and one sulfur atom were located. The result was subjected to the Tangens Expansion formula as implemented in SHELXS. Almost all non-hydrogen atoms were located. The remainder was found in the difference fourier map from SHELXL refinements. The PLATON SQUEEZE procedure (A. L. Spek. Acta Cryst. C71, 2015, 9-18) was used to treat regions of disordered solvent which could not be modelled in terms of atomic sites. The number of electrons found in these regions, 82, was assigned to 2 molecules of diethylether. 2 diethylethers would give 84 electrons.

### Bis[bis(1,3-dibenzyl-4,5-diphenylimidazol-2-ylidene)silver(I)] tris(thiocyanato)argentate(I) diethyl ether disolvate (1) Fractional atomic coordinates and isotropic or equivalent isotropic displacement parameters (Å^2^)

**Table:**

	*x*	*y*	*z*	*U*_iso_*/*U*_eq_	Occ. (<1)
Ag1	0.98105 (2)	0.78382 (2)	−0.03379 (2)	0.02968 (4)	
C1	1.21022 (18)	0.70910 (14)	−0.00838 (13)	0.0331 (5)	
H1	1.2339	0.7517	0.0297	0.040*	
C2	1.2083 (2)	0.64179 (15)	0.00485 (16)	0.0424 (6)	
H2	1.2296	0.6383	0.0519	0.051*	
C3	1.1753 (2)	0.57962 (15)	−0.05035 (18)	0.0507 (7)	
H3	1.1755	0.5334	−0.0415	0.061*	
C4	1.1422 (2)	0.58496 (15)	−0.11839 (18)	0.0519 (7)	
H4	1.1180	0.5423	−0.1562	0.062*	
C5	1.14406 (19)	0.65222 (14)	−0.13158 (13)	0.0364 (5)	
H5	1.1221	0.6555	−0.1787	0.044*	
C6	1.17778 (15)	0.71508 (12)	−0.07678 (11)	0.0252 (4)	
C7	1.17739 (17)	0.78740 (12)	−0.09409 (11)	0.0266 (4)	
H7A	1.2299	0.7994	−0.1166	0.032*	
H7B	1.1181	0.7839	−0.1284	0.032*	
N1	1.18670 (13)	0.84520 (10)	−0.03008 (9)	0.0227 (3)	
C8	1.11579 (15)	0.85001 (12)	0.00299 (11)	0.0249 (4)	
C9	1.27183 (15)	0.88709 (11)	0.01320 (11)	0.0222 (4)	
C10	1.36079 (17)	0.89170 (12)	−0.01006 (13)	0.0295 (5)	
C11	1.3764 (2)	0.92388 (17)	−0.06298 (14)	0.0426 (6)	
H11	1.3296	0.9450	−0.0850	0.051*	
C12	1.4622 (2)	0.9258 (2)	−0.08513 (16)	0.0554 (9)	
H12	1.4715	0.9461	−0.1234	0.066*	
C13	1.5306 (3)	0.8988 (2)	−0.0515 (2)	0.0630 (9)	
H13	1.5880	0.9003	−0.0662	0.076*	
C14	1.5177 (3)	0.8688 (2)	0.0043 (3)	0.0773 (13)	
H14	1.5669	0.8513	0.0285	0.093*	
C15	1.4337 (2)	0.8643 (2)	0.0246 (2)	0.0573 (9)	
H15	1.4246	0.8427	0.0620	0.069*	
C16	1.25234 (14)	0.92016 (11)	0.07505 (11)	0.0226 (4)	
C17	1.31529 (15)	0.96909 (12)	0.14136 (11)	0.0256 (4)	
C18	1.31124 (18)	1.04121 (14)	0.16332 (13)	0.0344 (5)	
H18	1.2679	1.0604	0.1351	0.041*	
C19	1.3699 (2)	1.08602 (16)	0.22635 (14)	0.0423 (6)	
H19	1.3666	1.1355	0.2406	0.051*	
C20	1.4316 (2)	1.05965 (17)	0.26750 (14)	0.0467 (7)	
H20	1.4726	1.0908	0.3098	0.056*	
C21	1.4348 (3)	0.9874 (2)	0.24781 (18)	0.0632 (10)	
H21	1.4764	0.9684	0.2776	0.076*	
C22	1.3773 (2)	0.94223 (17)	0.18435 (17)	0.0533 (8)	
H22	1.3808	0.8927	0.1706	0.064*	
N2	1.15577 (13)	0.89728 (10)	0.06686 (9)	0.0247 (4)	
C23	1.10662 (16)	0.91377 (14)	0.12398 (12)	0.0326 (5)	
H23A	1.0382	0.8958	0.1046	0.039*	
H23B	1.1189	0.9669	0.1437	0.039*	
C24	1.13906 (18)	0.87875 (14)	0.18219 (13)	0.0343 (5)	
C25	1.1708 (2)	0.92001 (16)	0.25172 (14)	0.0421 (6)	
H25	1.1703	0.9702	0.2630	0.051*	
C26	1.2033 (3)	0.88852 (19)	0.30501 (15)	0.0544 (8)	
H26	1.2244	0.9171	0.3528	0.065*	
C27	1.2051 (3)	0.8163 (2)	0.28929 (18)	0.0653 (10)	
H27	1.2287	0.7951	0.3259	0.078*	
C28	1.1725 (3)	0.77426 (18)	0.21979 (18)	0.0627 (10)	
H28	1.1735	0.7241	0.2087	0.075*	
C29	1.1384 (2)	0.80514 (15)	0.16650 (15)	0.0467 (7)	
H29	1.1146	0.7760	0.1191	0.056*	
C30	0.7594 (2)	0.86196 (16)	0.02635 (14)	0.0430 (6)	
H30	0.7151	0.8203	0.0260	0.052*	
C31	0.7872 (3)	0.9201 (2)	0.08573 (18)	0.0684 (11)	
H31	0.7614	0.9181	0.1257	0.082*	
C32	0.8500 (4)	0.9794 (2)	0.0880 (2)	0.0846 (17)	
H32	0.8678	1.0193	0.1290	0.102*	
C33	0.8881 (3)	0.98228 (17)	0.0312 (3)	0.0818 (16)	
H33	0.9329	1.0242	0.0331	0.098*	
C34	0.8622 (2)	0.92402 (15)	−0.03031 (18)	0.0521 (8)	
H34	0.8898	0.9259	−0.0695	0.063*	
C35	0.79556 (17)	0.86395 (12)	−0.03230 (12)	0.0312 (5)	
C36	0.76054 (18)	0.80201 (12)	−0.09808 (11)	0.0297 (5)	
H36A	0.6947	0.8014	−0.1199	0.036*	
H36B	0.7986	0.8093	−0.1328	0.036*	
N3	0.76572 (13)	0.73222 (10)	−0.08206 (9)	0.0238 (4)	
C37	0.84843 (15)	0.71588 (12)	−0.05818 (11)	0.0252 (4)	
C38	0.68993 (15)	0.67697 (11)	−0.08559 (10)	0.0220 (4)	
C39	0.59068 (15)	0.67846 (11)	−0.11029 (11)	0.0233 (4)	
C40	0.55652 (17)	0.68480 (13)	−0.17863 (11)	0.0288 (5)	
H40	0.5977	0.6885	−0.2095	0.035*	
C41	0.46292 (18)	0.68568 (15)	−0.20165 (13)	0.0358 (5)	
H41	0.4404	0.6901	−0.2482	0.043*	
C42	0.40217 (18)	0.68017 (16)	−0.15754 (15)	0.0404 (6)	
H42	0.3382	0.6813	−0.1734	0.049*	
C43	0.4349 (2)	0.67302 (18)	−0.08997 (15)	0.0450 (7)	
H43	0.3931	0.6687	−0.0596	0.054*	
C44	0.52835 (18)	0.67213 (15)	−0.06656 (13)	0.0344 (5)	
H44	0.5502	0.6672	−0.0201	0.041*	
C45	0.72822 (15)	0.62438 (12)	−0.06349 (11)	0.0231 (4)	
C46	0.68002 (15)	0.55211 (12)	−0.06161 (11)	0.0250 (4)	
C47A	0.6756 (10)	0.4947 (3)	−0.1191 (3)	0.0382 (19)	0.55 (2)
H47A	0.7054	0.5019	−0.1561	0.046*	0.55 (2)
C48A	0.6263 (11)	0.4262 (4)	−0.1213 (4)	0.047 (2)	0.55 (2)
H48A	0.6223	0.3858	−0.1598	0.057*	0.55 (2)
C47B	0.6300 (10)	0.4998 (4)	−0.1238 (4)	0.030 (2)	0.45 (2)
H47B	0.6306	0.5101	−0.1683	0.036*	0.45 (2)
C48B	0.5799 (10)	0.4335 (4)	−0.1225 (4)	0.035 (2)	0.45 (2)
H48B	0.5449	0.3999	−0.1657	0.042*	0.45 (2)
C49	0.5803 (2)	0.41677 (14)	−0.06271 (16)	0.0413 (6)	
H49A	0.5496	0.3695	−0.0633	0.050*	0.45 (2)
H49	0.5445	0.3710	−0.0637	0.050*	0.55 (2)
C50A	0.5907 (9)	0.4743 (5)	−0.0091 (7)	0.046 (2)	0.55 (2)
H50A	0.5638	0.4690	0.0297	0.056*	0.55 (2)
C51A	0.6397 (9)	0.5417 (5)	−0.0082 (6)	0.0385 (19)	0.55 (2)
H51A	0.6451	0.5817	0.0310	0.046*	0.55 (2)
C50B	0.6251 (10)	0.4670 (7)	0.0033 (6)	0.039 (2)	0.45 (2)
H50B	0.6214	0.4548	0.0467	0.046*	0.45 (2)
C51B	0.6748 (9)	0.5350 (6)	0.0034 (5)	0.032 (2)	0.45 (2)
H51B	0.7052	0.5696	0.0472	0.038*	0.45 (2)
N4	0.82539 (13)	0.64955 (10)	−0.04759 (9)	0.0253 (4)	
C52	0.89538 (16)	0.60961 (13)	−0.02429 (12)	0.0309 (5)	
H52A	0.9519	0.6241	−0.0418	0.037*	
H52B	0.8699	0.5572	−0.0464	0.037*	
C53	0.92407 (16)	0.62147 (12)	0.05521 (12)	0.0275 (4)	
C54	0.9906 (2)	0.58529 (14)	0.08136 (15)	0.0407 (6)	
H54	1.0179	0.5561	0.0493	0.049*	
C55	1.0175 (2)	0.59117 (17)	0.15341 (17)	0.0513 (8)	
H55	1.0625	0.5658	0.1706	0.062*	
C56	0.9789 (2)	0.6341 (2)	0.20018 (17)	0.0566 (9)	
H56	0.9965	0.6376	0.2496	0.068*	
C57	0.9145 (2)	0.6719 (2)	0.17531 (16)	0.0555 (8)	
H57	0.8892	0.7025	0.2077	0.067*	
C58	0.88663 (18)	0.66519 (17)	0.10261 (13)	0.0387 (6)	
H58	0.8418	0.6907	0.0856	0.046*	
Ag2	0.75371 (2)	0.69309 (2)	0.54662 (2)	0.01911 (4)	
C59	0.76475 (15)	0.50709 (11)	0.46307 (11)	0.0225 (4)	
H59	0.8247	0.5304	0.4598	0.027*	
C60	0.69686 (16)	0.46941 (12)	0.40157 (11)	0.0260 (4)	
H60	0.7108	0.4670	0.3564	0.031*	
C61	0.60914 (16)	0.43546 (12)	0.40584 (12)	0.0263 (4)	
H61	0.5633	0.4095	0.3638	0.032*	
C62	0.58851 (16)	0.43949 (12)	0.47170 (12)	0.0274 (4)	
H62	0.5282	0.4166	0.4747	0.033*	
C63	0.65595 (15)	0.47693 (12)	0.53319 (11)	0.0249 (4)	
H63	0.6416	0.4796	0.5782	0.030*	
C64	0.74483 (14)	0.51063 (10)	0.52924 (11)	0.0198 (4)	
C65	0.81645 (14)	0.54679 (11)	0.59853 (10)	0.0199 (4)	
H65A	0.7839	0.5696	0.6337	0.024*	
H65B	0.8430	0.5090	0.6165	0.024*	
N5	0.89381 (11)	0.60198 (9)	0.59393 (8)	0.0176 (3)	
C66	0.88138 (14)	0.66826 (11)	0.58793 (10)	0.0183 (4)	
C67	0.98878 (14)	0.60157 (11)	0.61353 (10)	0.0180 (4)	
C68	1.02285 (14)	0.53624 (11)	0.62232 (10)	0.0190 (4)	
C69	0.98347 (14)	0.46886 (11)	0.57308 (11)	0.0218 (4)	
H69	0.9343	0.4652	0.5334	0.026*	
C70	1.01597 (16)	0.40742 (11)	0.58206 (12)	0.0255 (4)	
H70	0.9884	0.3619	0.5488	0.031*	
C71	1.08858 (17)	0.41245 (12)	0.63940 (13)	0.0286 (5)	
H71	1.1109	0.3705	0.6453	0.034*	
C72	1.12857 (17)	0.47899 (13)	0.68824 (12)	0.0288 (5)	
H72	1.1782	0.4825	0.7276	0.035*	
C73	1.09603 (15)	0.54048 (12)	0.67962 (11)	0.0239 (4)	
H73	1.1239	0.5859	0.7131	0.029*	
C74	1.03660 (14)	0.66983 (10)	0.62029 (10)	0.0176 (4)	
C75	1.13793 (14)	0.70362 (10)	0.64412 (10)	0.0190 (4)	
C76	1.20124 (15)	0.68387 (11)	0.60387 (11)	0.0229 (4)	
H76	1.1799	0.6467	0.5607	0.027*	
C77	1.29550 (15)	0.71837 (12)	0.62661 (12)	0.0262 (4)	
H77	1.3383	0.7049	0.5988	0.031*	
C78	1.32722 (15)	0.77236 (12)	0.68963 (13)	0.0278 (4)	
H78	1.3915	0.7963	0.7047	0.033*	
C79	1.26479 (16)	0.79140 (12)	0.73066 (11)	0.0265 (4)	
H79	1.2868	0.8279	0.7743	0.032*	
C80	1.17026 (15)	0.75747 (11)	0.70843 (11)	0.0231 (4)	
H80	1.1278	0.7707	0.7367	0.028*	
N6	0.96896 (11)	0.70968 (9)	0.60418 (8)	0.0174 (3)	
C81	0.98813 (14)	0.78589 (10)	0.60104 (10)	0.0203 (4)	
H81A	1.0433	0.8135	0.6395	0.024*	
H81B	0.9337	0.8070	0.6093	0.024*	
C82	1.00677 (15)	0.79387 (10)	0.53060 (11)	0.0208 (4)	
C83	1.09775 (16)	0.80758 (12)	0.52096 (12)	0.0266 (4)	
H83	1.1495	0.8135	0.5595	0.032*	
C84	1.11334 (18)	0.81269 (13)	0.45497 (14)	0.0337 (5)	
H84	1.1756	0.8219	0.4487	0.040*	
C85	1.0382 (2)	0.80441 (14)	0.39856 (13)	0.0379 (6)	
H85	1.0488	0.8070	0.3534	0.045*	
C86	0.9477 (2)	0.79239 (15)	0.40830 (13)	0.0377 (6)	
H86	0.8961	0.7876	0.3699	0.045*	
C87	0.93200 (17)	0.78729 (13)	0.47376 (12)	0.0293 (5)	
H87	0.8696	0.7792	0.4800	0.035*	
C88	0.66481 (17)	0.64638 (13)	0.33252 (12)	0.0301 (5)	
H88	0.6923	0.6888	0.3701	0.036*	
C89	0.6978 (2)	0.63552 (17)	0.27051 (13)	0.0405 (6)	
H89	0.7481	0.6703	0.2662	0.049*	
C90	0.6575 (2)	0.57434 (18)	0.21551 (14)	0.0491 (7)	
H90	0.6797	0.5672	0.1732	0.059*	
C91	0.5850 (3)	0.52357 (17)	0.22198 (14)	0.0540 (8)	
H91	0.5574	0.4813	0.1842	0.065*	
C92	0.5522 (2)	0.53423 (14)	0.28367 (13)	0.0412 (6)	
H92	0.5020	0.4993	0.2878	0.049*	
C93	0.59255 (16)	0.59583 (12)	0.33951 (11)	0.0246 (4)	
C94	0.55979 (15)	0.60271 (11)	0.40821 (11)	0.0214 (4)	
H94A	0.4955	0.5727	0.3977	0.026*	
H94B	0.6012	0.5830	0.4408	0.026*	
N7	0.55944 (12)	0.67719 (9)	0.44431 (8)	0.0182 (3)	
C95	0.63428 (14)	0.72101 (11)	0.49374 (10)	0.0186 (4)	
C96	0.48627 (14)	0.71328 (11)	0.43243 (10)	0.0202 (4)	
C97	0.38961 (14)	0.67823 (11)	0.39126 (11)	0.0230 (4)	
C98	0.36889 (17)	0.63429 (14)	0.32221 (12)	0.0321 (5)	
H98	0.4179	0.6280	0.2984	0.039*	
C99	0.27620 (19)	0.59946 (15)	0.28787 (14)	0.0408 (6)	
H99	0.2625	0.5686	0.2412	0.049*	
C100	0.20452 (17)	0.60977 (14)	0.32153 (15)	0.0395 (6)	
H100	0.1417	0.5856	0.2981	0.047*	
C101	0.22370 (16)	0.65493 (13)	0.38890 (15)	0.0357 (5)	
H101	0.1740	0.6632	0.4114	0.043*	
C102	0.31620 (16)	0.68858 (12)	0.42410 (13)	0.0292 (5)	
H102	0.3293	0.7189	0.4710	0.035*	
C103	0.51846 (14)	0.78202 (11)	0.47479 (10)	0.0198 (4)	
C104	0.46956 (14)	0.84285 (11)	0.48426 (11)	0.0230 (4)	
C105	0.43043 (16)	0.85935 (12)	0.54279 (12)	0.0276 (4)	
H105	0.4387	0.8338	0.5784	0.033*	
C106	0.37929 (18)	0.91328 (14)	0.54867 (14)	0.0365 (5)	
H106	0.3520	0.9244	0.5883	0.044*	
C107	0.3679 (2)	0.95088 (15)	0.49722 (17)	0.0437 (6)	
H107	0.3328	0.9877	0.5015	0.052*	
C108	0.4073 (2)	0.93514 (17)	0.43954 (18)	0.0477 (7)	
H108	0.3997	0.9614	0.4044	0.057*	
C109	0.45788 (19)	0.88118 (14)	0.43285 (14)	0.0360 (5)	
H109	0.4847	0.8703	0.3930	0.043*	
N8	0.60883 (12)	0.78514 (9)	0.51169 (8)	0.0188 (3)	
C110	0.67195 (14)	0.84941 (11)	0.56130 (10)	0.0212 (4)	
H11A	0.7090	0.8347	0.6005	0.025*	
H11B	0.6342	0.8823	0.5821	0.025*	
C111	0.73840 (14)	0.89022 (11)	0.52734 (11)	0.0214 (4)	
C112	0.81556 (17)	0.94234 (12)	0.57147 (12)	0.0294 (5)	
H112	0.8272	0.9490	0.6214	0.035*	
C113	0.87523 (19)	0.98436 (14)	0.54315 (15)	0.0394 (6)	
H113	0.9270	1.0201	0.5738	0.047*	
C114	0.85991 (19)	0.97460 (14)	0.47042 (16)	0.0394 (6)	
H114	0.9006	1.0039	0.4511	0.047*	
C115	0.78475 (18)	0.92178 (14)	0.42575 (13)	0.0333 (5)	
H115	0.7748	0.9142	0.3757	0.040*	
C116	0.72393 (16)	0.87995 (12)	0.45408 (12)	0.0258 (4)	
H116	0.6723	0.8442	0.4233	0.031*	
Ag3	0.79941 (2)	0.72828 (2)	0.71384 (2)	0.02795 (4)	
S1	0.64189 (4)	0.65134 (3)	0.65701 (3)	0.02631 (10)	
C117	0.57874 (15)	0.71425 (12)	0.64988 (11)	0.0240 (4)	
N9	0.53253 (14)	0.75603 (11)	0.64467 (11)	0.0297 (4)	
S2	0.84839 (4)	0.86528 (3)	0.74115 (3)	0.03126 (12)	
C118	0.96150 (18)	0.86638 (12)	0.77086 (12)	0.0295 (5)	
N10	1.04011 (16)	0.86674 (12)	0.79091 (13)	0.0412 (5)	
S3	0.93489 (4)	0.69294 (3)	0.78243 (3)	0.02904 (11)	
C119	0.89197 (18)	0.61118 (13)	0.79230 (12)	0.0322 (5)	
N11	0.8653 (2)	0.55532 (13)	0.80169 (13)	0.0496 (6)	
C120	0.9168 (4)	0.8774 (4)	0.2474 (3)	0.136 (3)	
H12A	0.8818	0.8463	0.2708	0.204*	
H12B	0.9833	0.8758	0.2591	0.204*	
H12C	0.8922	0.8603	0.1958	0.204*	
C121	0.9060 (3)	0.9548 (4)	0.2729 (2)	0.099 (2)	
H12D	0.9371	0.9747	0.3239	0.119*	
H12E	0.9350	0.9858	0.2458	0.119*	
O1	0.81017 (17)	0.95296 (14)	0.26223 (11)	0.0551 (5)	
C122	0.7881 (4)	1.0237 (2)	0.2809 (2)	0.0799 (14)	
H12F	0.8157	1.0552	0.2536	0.096*	
H12G	0.8141	1.0469	0.3322	0.096*	
C123	0.6848 (4)	1.0135 (2)	0.2638 (3)	0.0803 (14)	
H12H	0.6600	0.9920	0.2127	0.120*	
H12I	0.6678	1.0606	0.2773	0.120*	
H12J	0.6581	0.9812	0.2901	0.120*	

### Bis[bis(1,3-dibenzyl-4,5-diphenylimidazol-2-ylidene)silver(I)] tris(thiocyanato)argentate(I) diethyl ether disolvate (1) Atomic displacement parameters (Å^2^)

**Table:**

	*U*^11^	*U*^22^	*U*^33^	*U*^12^	*U*^13^	*U*^23^
Ag1	0.02005 (8)	0.03206 (9)	0.02983 (8)	−0.00063 (6)	0.00112 (6)	0.00219 (6)
C1	0.0323 (12)	0.0319 (12)	0.0317 (11)	0.0030 (10)	0.0026 (9)	0.0088 (9)
C2	0.0401 (15)	0.0414 (14)	0.0482 (15)	0.0082 (12)	0.0044 (12)	0.0220 (12)
C3	0.0563 (19)	0.0295 (13)	0.069 (2)	0.0084 (12)	0.0133 (15)	0.0201 (13)
C4	0.062 (2)	0.0268 (13)	0.0566 (17)	0.0042 (13)	0.0075 (15)	0.0003 (12)
C5	0.0387 (14)	0.0317 (12)	0.0320 (12)	0.0053 (10)	0.0048 (10)	−0.0007 (10)
C6	0.0200 (10)	0.0262 (10)	0.0272 (10)	0.0038 (8)	0.0049 (8)	0.0039 (8)
C7	0.0324 (12)	0.0255 (10)	0.0180 (9)	0.0041 (9)	0.0037 (8)	0.0015 (8)
N1	0.0224 (9)	0.0235 (8)	0.0191 (8)	0.0029 (7)	0.0026 (7)	0.0029 (6)
C8	0.0208 (10)	0.0271 (10)	0.0235 (10)	0.0031 (8)	0.0027 (8)	0.0033 (8)
C9	0.0197 (10)	0.0222 (9)	0.0224 (9)	0.0023 (8)	0.0028 (8)	0.0047 (7)
C10	0.0263 (11)	0.0249 (10)	0.0349 (11)	0.0015 (9)	0.0135 (9)	0.0006 (9)
C11	0.0337 (14)	0.0584 (17)	0.0352 (13)	0.0010 (12)	0.0091 (10)	0.0169 (12)
C12	0.0501 (18)	0.068 (2)	0.0377 (14)	−0.0167 (16)	0.0157 (13)	0.0104 (14)
C13	0.0454 (18)	0.060 (2)	0.093 (3)	0.0130 (16)	0.0425 (19)	0.0166 (19)
C14	0.046 (2)	0.085 (3)	0.135 (4)	0.0338 (19)	0.046 (2)	0.063 (3)
C15	0.0392 (16)	0.065 (2)	0.089 (2)	0.0229 (15)	0.0283 (16)	0.0452 (19)
C16	0.0175 (10)	0.0250 (10)	0.0227 (9)	0.0016 (8)	0.0046 (8)	0.0036 (8)
C17	0.0201 (10)	0.0302 (11)	0.0216 (9)	−0.0008 (8)	0.0038 (8)	0.0022 (8)
C18	0.0320 (12)	0.0346 (12)	0.0287 (11)	0.0054 (10)	0.0024 (9)	−0.0027 (9)
C19	0.0379 (14)	0.0408 (14)	0.0337 (13)	−0.0011 (11)	0.0046 (11)	−0.0093 (11)
C20	0.0368 (14)	0.0554 (17)	0.0295 (12)	−0.0097 (13)	−0.0041 (10)	−0.0020 (11)
C21	0.055 (2)	0.061 (2)	0.0525 (18)	0.0052 (16)	−0.0274 (15)	0.0109 (15)
C22	0.0471 (17)	0.0408 (15)	0.0533 (17)	0.0046 (13)	−0.0198 (14)	0.0044 (13)
N2	0.0189 (9)	0.0286 (9)	0.0231 (8)	0.0021 (7)	0.0052 (7)	0.0019 (7)
C23	0.0227 (11)	0.0401 (13)	0.0298 (11)	0.0012 (9)	0.0117 (9)	−0.0021 (9)
C24	0.0317 (12)	0.0361 (13)	0.0306 (11)	−0.0052 (10)	0.0163 (10)	0.0013 (9)
C25	0.0488 (16)	0.0429 (14)	0.0308 (12)	0.0014 (12)	0.0169 (11)	0.0015 (11)
C26	0.073 (2)	0.0573 (19)	0.0298 (13)	0.0016 (16)	0.0200 (14)	0.0077 (12)
C27	0.102 (3)	0.060 (2)	0.0434 (16)	0.007 (2)	0.0323 (18)	0.0267 (15)
C28	0.102 (3)	0.0379 (16)	0.0513 (17)	−0.0022 (17)	0.0335 (19)	0.0163 (13)
C29	0.0625 (19)	0.0358 (14)	0.0361 (13)	−0.0103 (13)	0.0218 (13)	0.0046 (11)
C30	0.0438 (15)	0.0458 (15)	0.0346 (13)	0.0136 (12)	0.0040 (11)	0.0024 (11)
C31	0.082 (3)	0.070 (2)	0.0405 (16)	0.041 (2)	−0.0061 (16)	−0.0142 (16)
C32	0.103 (3)	0.0426 (19)	0.070 (3)	0.036 (2)	−0.050 (3)	−0.0195 (18)
C33	0.077 (3)	0.0232 (14)	0.105 (3)	−0.0062 (15)	−0.058 (3)	0.0122 (17)
C34	0.0423 (16)	0.0340 (14)	0.0654 (19)	−0.0093 (12)	−0.0200 (14)	0.0238 (13)
C35	0.0315 (12)	0.0228 (10)	0.0330 (11)	0.0024 (9)	−0.0048 (9)	0.0076 (9)
C36	0.0367 (13)	0.0270 (11)	0.0233 (10)	0.0010 (9)	0.0017 (9)	0.0110 (8)
N3	0.0248 (9)	0.0221 (8)	0.0206 (8)	−0.0003 (7)	0.0020 (7)	0.0046 (6)
C37	0.0215 (10)	0.0283 (11)	0.0212 (9)	0.0006 (8)	0.0026 (8)	0.0024 (8)
C38	0.0223 (10)	0.0224 (10)	0.0193 (9)	0.0021 (8)	0.0032 (7)	0.0047 (7)
C39	0.0236 (10)	0.0215 (10)	0.0241 (10)	0.0051 (8)	0.0038 (8)	0.0055 (8)
C40	0.0284 (11)	0.0362 (12)	0.0222 (10)	0.0102 (9)	0.0050 (8)	0.0067 (9)
C41	0.0316 (13)	0.0481 (15)	0.0285 (11)	0.0157 (11)	0.0014 (9)	0.0106 (10)
C42	0.0241 (12)	0.0563 (16)	0.0450 (14)	0.0175 (11)	0.0054 (10)	0.0175 (12)
C43	0.0320 (14)	0.071 (2)	0.0445 (14)	0.0209 (13)	0.0181 (11)	0.0255 (14)
C44	0.0318 (13)	0.0485 (14)	0.0300 (11)	0.0148 (11)	0.0103 (9)	0.0172 (10)
C45	0.0216 (10)	0.0262 (10)	0.0205 (9)	0.0053 (8)	0.0037 (8)	0.0050 (8)
C46	0.0237 (10)	0.0252 (10)	0.0264 (10)	0.0076 (8)	0.0023 (8)	0.0087 (8)
C47A	0.051 (6)	0.030 (3)	0.032 (3)	0.004 (3)	0.008 (3)	0.0094 (19)
C48A	0.069 (7)	0.023 (3)	0.039 (3)	0.003 (3)	−0.004 (3)	0.007 (2)
C47B	0.037 (5)	0.026 (3)	0.025 (3)	0.002 (3)	0.003 (3)	0.008 (2)
C48B	0.043 (5)	0.023 (3)	0.032 (3)	0.003 (3)	0.001 (3)	0.002 (2)
C49	0.0378 (14)	0.0285 (12)	0.0535 (16)	0.0001 (10)	0.0000 (12)	0.0165 (11)
C50A	0.049 (6)	0.037 (3)	0.065 (5)	0.010 (4)	0.028 (5)	0.027 (3)
C51A	0.043 (5)	0.029 (3)	0.043 (4)	0.006 (3)	0.012 (4)	0.009 (3)
C50B	0.044 (6)	0.037 (4)	0.035 (4)	0.000 (4)	0.012 (4)	0.013 (3)
C51B	0.035 (5)	0.037 (4)	0.018 (3)	−0.007 (4)	0.003 (3)	0.008 (2)
N4	0.0211 (9)	0.0272 (9)	0.0237 (8)	0.0052 (7)	0.0012 (7)	0.0023 (7)
C52	0.0224 (11)	0.0345 (12)	0.0315 (11)	0.0105 (9)	−0.0001 (9)	0.0020 (9)
C53	0.0207 (10)	0.0269 (11)	0.0326 (11)	0.0009 (8)	0.0019 (8)	0.0099 (9)
C54	0.0360 (14)	0.0294 (12)	0.0478 (15)	0.0095 (10)	−0.0071 (11)	0.0045 (11)
C55	0.0502 (17)	0.0448 (16)	0.0539 (17)	0.0109 (13)	−0.0110 (14)	0.0216 (13)
C56	0.0475 (17)	0.090 (3)	0.0409 (15)	0.0136 (17)	0.0064 (13)	0.0389 (16)
C57	0.0427 (16)	0.099 (3)	0.0359 (14)	0.0271 (17)	0.0180 (12)	0.0252 (16)
C58	0.0291 (12)	0.0615 (17)	0.0331 (12)	0.0190 (12)	0.0101 (10)	0.0188 (12)
Ag2	0.01516 (7)	0.02111 (7)	0.02099 (7)	0.00652 (5)	0.00277 (5)	0.00464 (5)
C59	0.0174 (9)	0.0244 (10)	0.0258 (10)	0.0048 (8)	0.0069 (8)	0.0048 (8)
C60	0.0256 (11)	0.0266 (10)	0.0239 (10)	0.0059 (9)	0.0068 (8)	0.0015 (8)
C61	0.0241 (11)	0.0218 (10)	0.0271 (10)	0.0019 (8)	0.0019 (8)	0.0000 (8)
C62	0.0207 (10)	0.0269 (11)	0.0313 (11)	−0.0019 (8)	0.0057 (8)	0.0060 (9)
C63	0.0214 (10)	0.0267 (10)	0.0257 (10)	0.0010 (8)	0.0074 (8)	0.0066 (8)
C64	0.0171 (9)	0.0171 (9)	0.0247 (9)	0.0046 (7)	0.0045 (7)	0.0045 (7)
C65	0.0163 (9)	0.0213 (9)	0.0226 (9)	0.0034 (7)	0.0059 (7)	0.0063 (7)
N5	0.0148 (8)	0.0178 (8)	0.0195 (7)	0.0044 (6)	0.0031 (6)	0.0037 (6)
C66	0.0170 (9)	0.0207 (9)	0.0175 (8)	0.0058 (7)	0.0044 (7)	0.0040 (7)
C67	0.0160 (9)	0.0209 (9)	0.0172 (8)	0.0056 (7)	0.0047 (7)	0.0029 (7)
C68	0.0165 (9)	0.0198 (9)	0.0235 (9)	0.0063 (7)	0.0077 (7)	0.0072 (7)
C69	0.0182 (9)	0.0215 (9)	0.0261 (9)	0.0042 (8)	0.0064 (8)	0.0058 (8)
C70	0.0245 (11)	0.0193 (9)	0.0341 (11)	0.0055 (8)	0.0120 (9)	0.0048 (8)
C71	0.0297 (12)	0.0250 (10)	0.0395 (12)	0.0124 (9)	0.0147 (9)	0.0150 (9)
C72	0.0275 (11)	0.0328 (11)	0.0299 (11)	0.0122 (9)	0.0047 (9)	0.0138 (9)
C73	0.0236 (10)	0.0254 (10)	0.0230 (9)	0.0073 (8)	0.0044 (8)	0.0064 (8)
C74	0.0158 (9)	0.0202 (9)	0.0173 (8)	0.0067 (7)	0.0044 (7)	0.0035 (7)
C75	0.0163 (9)	0.0181 (9)	0.0222 (9)	0.0041 (7)	0.0033 (7)	0.0056 (7)
C76	0.0206 (10)	0.0190 (9)	0.0282 (10)	0.0059 (8)	0.0056 (8)	0.0035 (8)
C77	0.0198 (10)	0.0243 (10)	0.0373 (11)	0.0078 (8)	0.0109 (9)	0.0077 (9)
C78	0.0171 (10)	0.0258 (10)	0.0388 (12)	0.0025 (8)	0.0022 (8)	0.0103 (9)
C79	0.0243 (11)	0.0234 (10)	0.0258 (10)	0.0016 (8)	−0.0006 (8)	0.0021 (8)
C80	0.0204 (10)	0.0254 (10)	0.0223 (9)	0.0046 (8)	0.0055 (8)	0.0038 (8)
N6	0.0156 (8)	0.0181 (8)	0.0192 (7)	0.0057 (6)	0.0043 (6)	0.0045 (6)
C81	0.0209 (10)	0.0171 (9)	0.0227 (9)	0.0055 (7)	0.0052 (7)	0.0034 (7)
C82	0.0230 (10)	0.0157 (9)	0.0247 (9)	0.0051 (7)	0.0066 (8)	0.0054 (7)
C83	0.0250 (11)	0.0246 (10)	0.0315 (11)	0.0055 (8)	0.0071 (9)	0.0097 (8)
C84	0.0338 (13)	0.0337 (12)	0.0413 (13)	0.0085 (10)	0.0193 (10)	0.0160 (10)
C85	0.0512 (16)	0.0351 (13)	0.0322 (12)	0.0061 (11)	0.0168 (11)	0.0153 (10)
C86	0.0406 (14)	0.0409 (14)	0.0306 (12)	0.0025 (11)	0.0019 (10)	0.0170 (10)
C87	0.0255 (11)	0.0316 (11)	0.0327 (11)	0.0052 (9)	0.0051 (9)	0.0146 (9)
C88	0.0298 (12)	0.0352 (12)	0.0263 (10)	0.0080 (10)	0.0099 (9)	0.0065 (9)
C89	0.0397 (14)	0.0568 (17)	0.0314 (12)	0.0135 (12)	0.0169 (11)	0.0152 (11)
C90	0.067 (2)	0.0628 (19)	0.0240 (12)	0.0252 (16)	0.0191 (12)	0.0097 (12)
C91	0.086 (2)	0.0454 (16)	0.0249 (12)	0.0105 (16)	0.0151 (14)	−0.0025 (11)
C92	0.0588 (18)	0.0314 (13)	0.0261 (11)	0.0009 (12)	0.0077 (11)	0.0007 (9)
C93	0.0260 (11)	0.0257 (10)	0.0216 (9)	0.0085 (8)	0.0046 (8)	0.0041 (8)
C94	0.0208 (10)	0.0191 (9)	0.0238 (9)	0.0052 (7)	0.0052 (8)	0.0042 (7)
N7	0.0163 (8)	0.0189 (8)	0.0197 (7)	0.0052 (6)	0.0034 (6)	0.0050 (6)
C95	0.0173 (9)	0.0207 (9)	0.0193 (9)	0.0059 (7)	0.0058 (7)	0.0058 (7)
C96	0.0175 (9)	0.0218 (9)	0.0230 (9)	0.0069 (8)	0.0045 (7)	0.0079 (7)
C97	0.0175 (10)	0.0204 (9)	0.0300 (10)	0.0041 (8)	−0.0005 (8)	0.0096 (8)
C98	0.0248 (11)	0.0384 (13)	0.0283 (11)	0.0035 (10)	−0.0021 (9)	0.0082 (9)
C99	0.0340 (14)	0.0413 (14)	0.0355 (13)	−0.0008 (11)	−0.0102 (10)	0.0085 (11)
C100	0.0207 (11)	0.0328 (12)	0.0566 (16)	−0.0027 (9)	−0.0117 (10)	0.0182 (11)
C101	0.0169 (11)	0.0299 (12)	0.0615 (16)	0.0046 (9)	0.0062 (10)	0.0171 (11)
C102	0.0217 (11)	0.0245 (10)	0.0410 (12)	0.0052 (8)	0.0044 (9)	0.0096 (9)
C103	0.0168 (9)	0.0221 (9)	0.0206 (9)	0.0063 (7)	0.0023 (7)	0.0061 (7)
C104	0.0170 (9)	0.0224 (10)	0.0283 (10)	0.0062 (8)	0.0018 (8)	0.0051 (8)
C105	0.0250 (11)	0.0268 (11)	0.0278 (10)	0.0094 (9)	0.0019 (8)	0.0017 (8)
C106	0.0303 (12)	0.0353 (13)	0.0402 (13)	0.0149 (10)	0.0071 (10)	−0.0024 (10)
C107	0.0358 (14)	0.0348 (13)	0.0654 (18)	0.0231 (11)	0.0098 (13)	0.0130 (12)
C108	0.0467 (16)	0.0485 (16)	0.0673 (19)	0.0299 (14)	0.0192 (14)	0.0349 (15)
C109	0.0350 (13)	0.0412 (14)	0.0444 (13)	0.0202 (11)	0.0155 (11)	0.0228 (11)
N8	0.0167 (8)	0.0202 (8)	0.0192 (7)	0.0066 (6)	0.0024 (6)	0.0042 (6)
C110	0.0193 (10)	0.0212 (9)	0.0206 (9)	0.0044 (8)	0.0027 (7)	0.0021 (7)
C111	0.0193 (10)	0.0192 (9)	0.0271 (10)	0.0083 (8)	0.0054 (8)	0.0056 (7)
C112	0.0268 (11)	0.0262 (11)	0.0315 (11)	0.0031 (9)	0.0046 (9)	0.0039 (9)
C113	0.0318 (13)	0.0320 (12)	0.0484 (14)	−0.0032 (10)	0.0084 (11)	0.0066 (11)
C114	0.0359 (14)	0.0339 (13)	0.0558 (16)	0.0050 (11)	0.0217 (12)	0.0192 (11)
C115	0.0374 (13)	0.0367 (12)	0.0362 (12)	0.0163 (10)	0.0175 (10)	0.0170 (10)
C116	0.0251 (11)	0.0260 (10)	0.0286 (10)	0.0098 (8)	0.0073 (8)	0.0077 (8)
Ag3	0.02101 (8)	0.03363 (9)	0.02613 (8)	0.00446 (6)	0.00344 (6)	0.00431 (6)
S1	0.0207 (2)	0.0273 (2)	0.0305 (2)	0.00565 (19)	0.00707 (19)	0.0052 (2)
C117	0.0205 (10)	0.0260 (10)	0.0217 (9)	−0.0006 (8)	0.0063 (8)	0.0015 (8)
N9	0.0246 (9)	0.0307 (10)	0.0347 (10)	0.0072 (8)	0.0097 (8)	0.0069 (8)
S2	0.0326 (3)	0.0309 (3)	0.0323 (3)	0.0114 (2)	0.0085 (2)	0.0083 (2)
C118	0.0350 (13)	0.0242 (10)	0.0266 (10)	0.0047 (9)	0.0089 (9)	0.0006 (8)
N10	0.0338 (12)	0.0299 (11)	0.0511 (13)	0.0025 (9)	0.0049 (10)	−0.0003 (9)
S3	0.0251 (3)	0.0338 (3)	0.0254 (2)	0.0032 (2)	0.0011 (2)	0.0084 (2)
C119	0.0343 (13)	0.0339 (12)	0.0232 (10)	0.0057 (10)	0.0007 (9)	0.0032 (9)
N11	0.0614 (16)	0.0344 (12)	0.0413 (12)	−0.0008 (11)	−0.0036 (11)	0.0076 (10)
C120	0.090 (4)	0.268 (9)	0.169 (6)	0.122 (5)	0.092 (4)	0.182 (6)
C121	0.047 (2)	0.195 (6)	0.073 (3)	0.014 (3)	0.0118 (19)	0.083 (4)
O1	0.0558 (13)	0.0739 (15)	0.0417 (11)	0.0120 (11)	0.0185 (10)	0.0230 (10)
C122	0.132 (4)	0.049 (2)	0.057 (2)	−0.007 (2)	0.046 (2)	0.0105 (16)
C123	0.120 (4)	0.056 (2)	0.102 (3)	0.041 (2)	0.077 (3)	0.036 (2)

### Bis[bis(1,3-dibenzyl-4,5-diphenylimidazol-2-ylidene)silver(I)] tris(thiocyanato)argentate(I) diethyl ether disolvate (1) Geometric parameters (Å, º)

**Table:**

Ag1—C37	2.085 (2)	C60—H60	0.9500
Ag1—C8	2.091 (2)	C61—C62	1.387 (3)
C1—C2	1.383 (4)	C61—H61	0.9500
C1—C6	1.390 (3)	C62—C63	1.388 (3)
C1—H1	0.9500	C62—H62	0.9500
C2—C3	1.382 (4)	C63—C64	1.397 (3)
C2—H2	0.9500	C63—H63	0.9500
C3—C4	1.381 (5)	C64—C65	1.514 (3)
C3—H3	0.9500	C65—N5	1.465 (2)
C4—C5	1.382 (4)	C65—H65A	0.9900
C4—H4	0.9500	C65—H65B	0.9900
C5—C6	1.387 (3)	N5—C66	1.355 (3)
C5—H5	0.9500	N5—C67	1.394 (3)
C6—C7	1.515 (3)	C66—N6	1.350 (3)
C7—N1	1.463 (3)	C67—C74	1.358 (3)
C7—H7A	0.9900	C67—C68	1.472 (3)
C7—H7B	0.9900	C68—C73	1.396 (3)
N1—C8	1.348 (3)	C68—C69	1.403 (3)
N1—C9	1.393 (3)	C69—C70	1.391 (3)
C8—N2	1.350 (3)	C69—H69	0.9500
C9—C16	1.360 (3)	C70—C71	1.387 (3)
C9—C10	1.476 (3)	C70—H70	0.9500
C10—C11	1.371 (4)	C71—C72	1.390 (3)
C10—C15	1.414 (4)	C71—H71	0.9500
C11—C12	1.423 (4)	C72—C73	1.390 (3)
C11—H11	0.9500	C72—H72	0.9500
C12—C13	1.353 (5)	C73—H73	0.9500
C12—H12	0.9500	C74—N6	1.398 (2)
C13—C14	1.389 (6)	C74—C75	1.479 (3)
C13—H13	0.9500	C75—C76	1.392 (3)
C14—C15	1.378 (5)	C75—C80	1.401 (3)
C14—H14	0.9500	C76—C77	1.390 (3)
C15—H15	0.9500	C76—H76	0.9500
C16—N2	1.397 (3)	C77—C78	1.385 (3)
C16—C17	1.482 (3)	C77—H77	0.9500
C17—C22	1.382 (4)	C78—C79	1.388 (3)
C17—C18	1.383 (3)	C78—H78	0.9500
C18—C19	1.392 (3)	C79—C80	1.390 (3)
C18—H18	0.9500	C79—H79	0.9500
C19—C20	1.352 (4)	C80—H80	0.9500
C19—H19	0.9500	N6—C81	1.474 (2)
C20—C21	1.378 (5)	C81—C82	1.511 (3)
C20—H20	0.9500	C81—H81A	0.9900
C21—C22	1.394 (4)	C81—H81B	0.9900
C21—H21	0.9500	C82—C83	1.391 (3)
C22—H22	0.9500	C82—C87	1.394 (3)
N2—C23	1.467 (3)	C83—C84	1.394 (3)
C23—C24	1.508 (4)	C83—H83	0.9500
C23—H23A	0.9900	C84—C85	1.385 (4)
C23—H23B	0.9900	C84—H84	0.9500
C24—C25	1.382 (3)	C85—C86	1.385 (4)
C24—C29	1.389 (4)	C85—H85	0.9500
C25—C26	1.384 (5)	C86—C87	1.384 (3)
C25—H25	0.9500	C86—H86	0.9500
C26—C27	1.371 (5)	C87—H87	0.9500
C26—H26	0.9500	C88—C93	1.378 (3)
C27—C28	1.387 (5)	C88—C89	1.396 (3)
C27—H27	0.9500	C88—H88	0.9500
C28—C29	1.383 (5)	C89—C90	1.379 (4)
C28—H28	0.9500	C89—H89	0.9500
C29—H29	0.9500	C90—C91	1.379 (5)
C30—C35	1.376 (4)	C90—H90	0.9500
C30—C31	1.380 (4)	C91—C92	1.387 (4)
C30—H30	0.9500	C91—H91	0.9500
C31—C32	1.341 (7)	C92—C93	1.393 (3)
C31—H31	0.9500	C92—H92	0.9500
C32—C33	1.360 (8)	C93—C94	1.519 (3)
C32—H32	0.9500	C94—N7	1.463 (3)
C33—C34	1.409 (5)	C94—H94A	0.9900
C33—H33	0.9500	C94—H94B	0.9900
C34—C35	1.385 (4)	N7—C95	1.352 (3)
C34—H34	0.9500	N7—C96	1.399 (3)
C35—C36	1.503 (3)	C95—N8	1.351 (3)
C36—N3	1.466 (3)	C96—C103	1.361 (3)
C36—H36A	0.9900	C96—C97	1.477 (3)
C36—H36B	0.9900	C97—C98	1.392 (3)
N3—C37	1.347 (3)	C97—C102	1.392 (3)
N3—C38	1.398 (3)	C98—C99	1.397 (3)
C37—N4	1.346 (3)	C98—H98	0.9500
C38—C45	1.361 (3)	C99—C100	1.379 (4)
C38—C39	1.472 (3)	C99—H99	0.9500
C39—C44	1.392 (3)	C100—C101	1.375 (4)
C39—C40	1.396 (3)	C100—H100	0.9500
C40—C41	1.385 (3)	C101—C102	1.394 (3)
C40—H40	0.9500	C101—H101	0.9500
C41—C42	1.378 (4)	C102—H102	0.9500
C41—H41	0.9500	C103—N8	1.389 (3)
C42—C43	1.384 (4)	C103—C104	1.480 (3)
C42—H42	0.9500	C104—C109	1.391 (3)
C43—C44	1.384 (4)	C104—C105	1.394 (3)
C43—H43	0.9500	C105—C106	1.388 (3)
C44—H44	0.9500	C105—H105	0.9500
C45—N4	1.396 (3)	C106—C107	1.381 (4)
C45—C46	1.475 (3)	C106—H106	0.9500
C46—C51A	1.348 (9)	C107—C108	1.380 (4)
C46—C47A	1.390 (7)	C107—H107	0.9500
C46—C47B	1.399 (8)	C108—C109	1.383 (4)
C46—C51B	1.415 (9)	C108—H108	0.9500
C47A—C48A	1.392 (9)	C109—H109	0.9500
C47A—H47A	0.9500	N8—C110	1.457 (3)
C48A—C49	1.484 (12)	C110—C111	1.510 (3)
C48A—H48A	0.9500	C110—H11A	0.9900
C47B—C48B	1.382 (10)	C110—H11B	0.9900
C47B—H47B	0.9500	C111—C116	1.394 (3)
C48B—C49	1.297 (8)	C111—C112	1.396 (3)
C48B—H48B	0.9500	C112—C113	1.384 (4)
C49—C50A	1.325 (11)	C112—H112	0.9500
C49—C50B	1.415 (11)	C113—C114	1.384 (4)
C49—H49A	0.9500	C113—H113	0.9500
C49—H49	0.9500	C114—C115	1.388 (4)
C50A—C51A	1.379 (13)	C114—H114	0.9500
C50A—H50A	0.9500	C115—C116	1.393 (3)
C51A—H51A	0.9500	C115—H115	0.9500
C50B—C51B	1.399 (15)	C116—H116	0.9500
C50B—H50B	0.9500	Ag3—S1	2.4657 (5)
C51B—H51B	0.9500	Ag3—S3	2.4940 (6)
N4—C52	1.463 (3)	Ag3—S2	2.5377 (6)
C52—C53	1.510 (3)	S1—C117	1.671 (2)
C52—H52A	0.9900	C117—N9	1.154 (3)
C52—H52B	0.9900	S2—C118	1.669 (3)
C53—C58	1.384 (4)	C118—N10	1.160 (3)
C53—C54	1.392 (3)	S3—C119	1.674 (3)
C54—C55	1.385 (4)	C119—N11	1.156 (4)
C54—H54	0.9500	C120—C121	1.517 (9)
C55—C56	1.379 (5)	C120—H12A	0.9800
C55—H55	0.9500	C120—H12B	0.9800
C56—C57	1.384 (5)	C120—H12C	0.9800
C56—H56	0.9500	C121—O1	1.394 (5)
C57—C58	1.395 (4)	C121—H12D	0.9900
C57—H57	0.9500	C121—H12E	0.9900
C58—H58	0.9500	O1—C122	1.447 (5)
Ag2—C66	2.097 (2)	C122—C123	1.479 (7)
Ag2—C95	2.102 (2)	C122—H12F	0.9900
C59—C64	1.390 (3)	C122—H12G	0.9900
C59—C60	1.393 (3)	C123—H12H	0.9800
C59—H59	0.9500	C123—H12I	0.9800
C60—C61	1.386 (3)	C123—H12J	0.9800
			
C37—Ag1—C8	173.06 (8)	C60—C61—H61	120.1
C2—C1—C6	120.6 (2)	C62—C61—H61	120.1
C2—C1—H1	119.7	C61—C62—C63	120.0 (2)
C6—C1—H1	119.7	C61—C62—H62	120.0
C3—C2—C1	120.0 (3)	C63—C62—H62	120.0
C3—C2—H2	120.0	C62—C63—C64	120.4 (2)
C1—C2—H2	120.0	C62—C63—H63	119.8
C4—C3—C2	119.7 (3)	C64—C63—H63	119.8
C4—C3—H3	120.1	C59—C64—C63	119.32 (19)
C2—C3—H3	120.1	C59—C64—C65	123.21 (18)
C3—C4—C5	120.2 (3)	C63—C64—C65	117.42 (18)
C3—C4—H4	119.9	N5—C65—C64	114.90 (16)
C5—C4—H4	119.9	N5—C65—H65A	108.5
C4—C5—C6	120.7 (3)	C64—C65—H65A	108.5
C4—C5—H5	119.7	N5—C65—H65B	108.5
C6—C5—H5	119.7	C64—C65—H65B	108.5
C5—C6—C1	118.7 (2)	H65A—C65—H65B	107.5
C5—C6—C7	118.5 (2)	C66—N5—C67	111.26 (16)
C1—C6—C7	122.8 (2)	C66—N5—C65	121.43 (16)
N1—C7—C6	111.19 (17)	C67—N5—C65	125.21 (16)
N1—C7—H7A	109.4	N6—C66—N5	104.81 (16)
C6—C7—H7A	109.4	N6—C66—Ag2	128.78 (14)
N1—C7—H7B	109.4	N5—C66—Ag2	125.76 (14)
C6—C7—H7B	109.4	C74—C67—N5	106.39 (17)
H7A—C7—H7B	108.0	C74—C67—C68	130.61 (18)
C8—N1—C9	111.66 (17)	N5—C67—C68	122.99 (17)
C8—N1—C7	121.59 (18)	C73—C68—C69	118.72 (19)
C9—N1—C7	124.57 (18)	C73—C68—C67	120.38 (18)
N1—C8—N2	104.69 (18)	C69—C68—C67	120.90 (18)
N1—C8—Ag1	125.14 (15)	C70—C69—C68	120.41 (19)
N2—C8—Ag1	129.44 (16)	C70—C69—H69	119.8
C16—C9—N1	106.11 (18)	C68—C69—H69	119.8
C16—C9—C10	131.06 (19)	C71—C70—C69	120.2 (2)
N1—C9—C10	122.82 (19)	C71—C70—H70	119.9
C11—C10—C15	118.6 (2)	C69—C70—H70	119.9
C11—C10—C9	122.0 (2)	C70—C71—C72	119.9 (2)
C15—C10—C9	119.4 (2)	C70—C71—H71	120.0
C10—C11—C12	120.4 (3)	C72—C71—H71	120.0
C10—C11—H11	119.8	C71—C72—C73	120.1 (2)
C12—C11—H11	119.8	C71—C72—H72	120.0
C13—C12—C11	119.8 (3)	C73—C72—H72	120.0
C13—C12—H12	120.1	C72—C73—C68	120.7 (2)
C11—C12—H12	120.1	C72—C73—H73	119.7
C12—C13—C14	120.6 (3)	C68—C73—H73	119.7
C12—C13—H13	119.7	C67—C74—N6	106.25 (17)
C14—C13—H13	119.7	C67—C74—C75	131.52 (18)
C15—C14—C13	120.1 (4)	N6—C74—C75	122.10 (17)
C15—C14—H14	119.9	C76—C75—C80	119.53 (19)
C13—C14—H14	119.9	C76—C75—C74	121.65 (18)
C14—C15—C10	120.3 (3)	C80—C75—C74	118.82 (18)
C14—C15—H15	119.8	C77—C76—C75	120.21 (19)
C10—C15—H15	119.8	C77—C76—H76	119.9
C9—C16—N2	106.17 (18)	C75—C76—H76	119.9
C9—C16—C17	130.6 (2)	C78—C77—C76	120.3 (2)
N2—C16—C17	123.25 (18)	C78—C77—H77	119.9
C22—C17—C18	118.4 (2)	C76—C77—H77	119.9
C22—C17—C16	120.0 (2)	C77—C78—C79	119.8 (2)
C18—C17—C16	121.6 (2)	C77—C78—H78	120.1
C17—C18—C19	120.7 (3)	C79—C78—H78	120.1
C17—C18—H18	119.6	C78—C79—C80	120.5 (2)
C19—C18—H18	119.6	C78—C79—H79	119.8
C20—C19—C18	120.5 (3)	C80—C79—H79	119.8
C20—C19—H19	119.7	C79—C80—C75	119.7 (2)
C18—C19—H19	119.7	C79—C80—H80	120.2
C19—C20—C21	119.8 (2)	C75—C80—H80	120.2
C19—C20—H20	120.1	C66—N6—C74	111.27 (16)
C21—C20—H20	120.1	C66—N6—C81	122.92 (17)
C20—C21—C22	120.2 (3)	C74—N6—C81	125.73 (17)
C20—C21—H21	119.9	N6—C81—C82	112.59 (16)
C22—C21—H21	119.9	N6—C81—H81A	109.1
C17—C22—C21	120.3 (3)	C82—C81—H81A	109.1
C17—C22—H22	119.8	N6—C81—H81B	109.1
C21—C22—H22	119.8	C82—C81—H81B	109.1
C8—N2—C16	111.32 (18)	H81A—C81—H81B	107.8
C8—N2—C23	123.91 (19)	C83—C82—C87	118.8 (2)
C16—N2—C23	124.17 (18)	C83—C82—C81	121.23 (19)
N2—C23—C24	110.6 (2)	C87—C82—C81	119.93 (19)
N2—C23—H23A	109.5	C82—C83—C84	120.3 (2)
C24—C23—H23A	109.5	C82—C83—H83	119.8
N2—C23—H23B	109.5	C84—C83—H83	119.8
C24—C23—H23B	109.5	C85—C84—C83	120.1 (2)
H23A—C23—H23B	108.1	C85—C84—H84	119.9
C25—C24—C29	119.4 (3)	C83—C84—H84	119.9
C25—C24—C23	120.1 (2)	C86—C85—C84	119.7 (2)
C29—C24—C23	120.5 (2)	C86—C85—H85	120.1
C24—C25—C26	120.3 (3)	C84—C85—H85	120.1
C24—C25—H25	119.9	C87—C86—C85	120.2 (2)
C26—C25—H25	119.9	C87—C86—H86	119.9
C27—C26—C25	120.4 (3)	C85—C86—H86	119.9
C27—C26—H26	119.8	C86—C87—C82	120.7 (2)
C25—C26—H26	119.8	C86—C87—H87	119.7
C26—C27—C28	119.7 (3)	C82—C87—H87	119.7
C26—C27—H27	120.1	C93—C88—C89	120.3 (2)
C28—C27—H27	120.1	C93—C88—H88	119.8
C29—C28—C27	120.1 (3)	C89—C88—H88	119.8
C29—C28—H28	119.9	C90—C89—C88	120.1 (3)
C27—C28—H28	119.9	C90—C89—H89	120.0
C28—C29—C24	120.0 (3)	C88—C89—H89	120.0
C28—C29—H29	120.0	C91—C90—C89	120.0 (2)
C24—C29—H29	120.0	C91—C90—H90	120.0
C35—C30—C31	120.3 (3)	C89—C90—H90	120.0
C35—C30—H30	119.8	C90—C91—C92	120.0 (3)
C31—C30—H30	119.8	C90—C91—H91	120.0
C32—C31—C30	121.2 (4)	C92—C91—H91	120.0
C32—C31—H31	119.4	C91—C92—C93	120.4 (3)
C30—C31—H31	119.4	C91—C92—H92	119.8
C31—C32—C33	119.7 (3)	C93—C92—H92	119.8
C31—C32—H32	120.1	C88—C93—C92	119.2 (2)
C33—C32—H32	120.1	C88—C93—C94	121.89 (19)
C32—C33—C34	121.0 (4)	C92—C93—C94	118.8 (2)
C32—C33—H33	119.5	N7—C94—C93	113.90 (17)
C34—C33—H33	119.5	N7—C94—H94A	108.8
C35—C34—C33	118.4 (4)	C93—C94—H94A	108.8
C35—C34—H34	120.8	N7—C94—H94B	108.8
C33—C34—H34	120.8	C93—C94—H94B	108.8
C30—C35—C34	119.3 (3)	H94A—C94—H94B	107.7
C30—C35—C36	119.7 (2)	C95—N7—C96	111.11 (16)
C34—C35—C36	121.0 (3)	C95—N7—C94	122.37 (17)
N3—C36—C35	111.53 (17)	C96—N7—C94	126.49 (17)
N3—C36—H36A	109.3	N8—C95—N7	104.77 (17)
C35—C36—H36A	109.3	N8—C95—Ag2	125.79 (14)
N3—C36—H36B	109.3	N7—C95—Ag2	128.50 (14)
C35—C36—H36B	109.3	C103—C96—N7	106.17 (17)
H36A—C36—H36B	108.0	C103—C96—C97	128.07 (19)
C37—N3—C38	111.70 (18)	N7—C96—C97	125.00 (18)
C37—N3—C36	121.70 (19)	C98—C97—C102	118.8 (2)
C38—N3—C36	126.54 (19)	C98—C97—C96	123.2 (2)
N4—C37—N3	104.74 (18)	C102—C97—C96	118.02 (19)
N4—C37—Ag1	128.26 (16)	C97—C98—C99	120.1 (2)
N3—C37—Ag1	126.73 (16)	C97—C98—H98	119.9
C45—C38—N3	105.77 (18)	C99—C98—H98	119.9
C45—C38—C39	129.59 (19)	C100—C99—C98	120.2 (2)
N3—C38—C39	124.63 (19)	C100—C99—H99	119.9
C44—C39—C40	118.5 (2)	C98—C99—H99	119.9
C44—C39—C38	120.78 (19)	C101—C100—C99	120.2 (2)
C40—C39—C38	120.7 (2)	C101—C100—H100	119.9
C41—C40—C39	120.4 (2)	C99—C100—H100	119.9
C41—C40—H40	119.8	C100—C101—C102	119.9 (2)
C39—C40—H40	119.8	C100—C101—H101	120.1
C42—C41—C40	120.5 (2)	C102—C101—H101	120.1
C42—C41—H41	119.7	C97—C102—C101	120.7 (2)
C40—C41—H41	119.7	C97—C102—H102	119.6
C41—C42—C43	119.7 (2)	C101—C102—H102	119.6
C41—C42—H42	120.2	C96—C103—N8	106.35 (17)
C43—C42—H42	120.2	C96—C103—C104	129.11 (19)
C44—C43—C42	120.2 (2)	N8—C103—C104	124.51 (18)
C44—C43—H43	119.9	C109—C104—C105	119.6 (2)
C42—C43—H43	119.9	C109—C104—C103	120.2 (2)
C43—C44—C39	120.8 (2)	C105—C104—C103	120.07 (19)
C43—C44—H44	119.6	C106—C105—C104	119.6 (2)
C39—C44—H44	119.6	C106—C105—H105	120.2
C38—C45—N4	106.32 (18)	C104—C105—H105	120.2
C38—C45—C46	128.4 (2)	C107—C106—C105	120.3 (2)
N4—C45—C46	125.13 (19)	C107—C106—H106	119.9
C51A—C46—C47A	121.0 (5)	C105—C106—H106	119.9
C47B—C46—C51B	116.9 (5)	C108—C107—C106	120.2 (2)
C51A—C46—C45	122.4 (4)	C108—C107—H107	119.9
C47A—C46—C45	116.6 (3)	C106—C107—H107	119.9
C47B—C46—C45	121.5 (3)	C107—C108—C109	120.0 (3)
C51B—C46—C45	121.4 (4)	C107—C108—H108	120.0
C46—C47A—C48A	118.4 (6)	C109—C108—H108	120.0
C46—C47A—H47A	120.8	C108—C109—C104	120.2 (2)
C48A—C47A—H47A	120.8	C108—C109—H109	119.9
C47A—C48A—C49	119.4 (6)	C104—C109—H109	119.9
C47A—C48A—H48A	120.3	C95—N8—C103	111.58 (16)
C49—C48A—H48A	120.3	C95—N8—C110	122.79 (17)
C48B—C47B—C46	122.0 (6)	C103—N8—C110	125.57 (17)
C48B—C47B—H47B	119.0	N8—C110—C111	113.24 (16)
C46—C47B—H47B	119.0	N8—C110—H11A	108.9
C49—C48B—C47B	120.5 (6)	C111—C110—H11A	108.9
C49—C48B—H48B	119.8	N8—C110—H11B	108.9
C47B—C48B—H48B	119.8	C111—C110—H11B	108.9
C48B—C49—C50B	121.9 (6)	H11A—C110—H11B	107.7
C50A—C49—C48A	117.9 (5)	C116—C111—C112	118.8 (2)
C48B—C49—H49A	119.0	C116—C111—C110	122.77 (19)
C50B—C49—H49A	119.0	C112—C111—C110	118.43 (19)
C50A—C49—H49	121.1	C113—C112—C111	120.7 (2)
C48A—C49—H49	121.1	C113—C112—H112	119.7
C49—C50A—C51A	121.7 (8)	C111—C112—H112	119.7
C49—C50A—H50A	119.2	C114—C113—C112	120.4 (2)
C51A—C50A—H50A	119.2	C114—C113—H113	119.8
C46—C51A—C50A	121.6 (8)	C112—C113—H113	119.8
C46—C51A—H51A	119.2	C113—C114—C115	119.6 (2)
C50A—C51A—H51A	119.2	C113—C114—H114	120.2
C51B—C50B—C49	118.5 (8)	C115—C114—H114	120.2
C51B—C50B—H50B	120.7	C114—C115—C116	120.2 (2)
C49—C50B—H50B	120.7	C114—C115—H115	119.9
C50B—C51B—C46	120.0 (8)	C116—C115—H115	119.9
C50B—C51B—H51B	120.0	C115—C116—C111	120.4 (2)
C46—C51B—H51B	120.0	C115—C116—H116	119.8
C37—N4—C45	111.46 (18)	C111—C116—H116	119.8
C37—N4—C52	122.88 (19)	S1—Ag3—S3	126.936 (19)
C45—N4—C52	125.64 (19)	S1—Ag3—S2	127.68 (2)
N4—C52—C53	114.39 (19)	S3—Ag3—S2	103.957 (19)
N4—C52—H52A	108.7	C117—S1—Ag3	100.90 (7)
C53—C52—H52A	108.7	N9—C117—S1	177.8 (2)
N4—C52—H52B	108.7	C118—S2—Ag3	93.45 (8)
C53—C52—H52B	108.7	N10—C118—S2	179.4 (3)
H52A—C52—H52B	107.6	C119—S3—Ag3	105.56 (9)
C58—C53—C54	118.9 (2)	N11—C119—S3	177.1 (2)
C58—C53—C52	123.4 (2)	C121—C120—H12A	109.5
C54—C53—C52	117.7 (2)	C121—C120—H12B	109.5
C55—C54—C53	120.9 (3)	H12A—C120—H12B	109.5
C55—C54—H54	119.5	C121—C120—H12C	109.5
C53—C54—H54	119.5	H12A—C120—H12C	109.5
C56—C55—C54	119.7 (3)	H12B—C120—H12C	109.5
C56—C55—H55	120.1	O1—C121—C120	107.2 (4)
C54—C55—H55	120.1	O1—C121—H12D	110.3
C55—C56—C57	120.1 (3)	C120—C121—H12D	110.3
C55—C56—H56	119.9	O1—C121—H12E	110.3
C57—C56—H56	119.9	C120—C121—H12E	110.3
C56—C57—C58	120.0 (3)	H12D—C121—H12E	108.5
C56—C57—H57	120.0	C121—O1—C122	113.9 (4)
C58—C57—H57	120.0	O1—C122—C123	107.7 (3)
C53—C58—C57	120.3 (3)	O1—C122—H12F	110.2
C53—C58—H58	119.9	C123—C122—H12F	110.2
C57—C58—H58	119.9	O1—C122—H12G	110.2
C66—Ag2—C95	172.01 (7)	C123—C122—H12G	110.2
C64—C59—C60	120.02 (19)	H12F—C122—H12G	108.5
C64—C59—H59	120.0	C122—C123—H12H	109.5
C60—C59—H59	120.0	C122—C123—H12I	109.5
C61—C60—C59	120.4 (2)	H12H—C123—H12I	109.5
C61—C60—H60	119.8	C122—C123—H12J	109.5
C59—C60—H60	119.8	H12H—C123—H12J	109.5
C60—C61—C62	119.8 (2)	H12I—C123—H12J	109.5
			
C6—C1—C2—C3	1.1 (4)	C46—C45—N4—C52	−2.1 (3)
C1—C2—C3—C4	−1.6 (5)	C37—N4—C52—C53	92.3 (3)
C2—C3—C4—C5	1.6 (5)	C45—N4—C52—C53	−89.6 (3)
C3—C4—C5—C6	−1.0 (5)	N4—C52—C53—C58	1.2 (3)
C4—C5—C6—C1	0.4 (4)	N4—C52—C53—C54	−179.9 (2)
C4—C5—C6—C7	−179.4 (3)	C58—C53—C54—C55	1.6 (4)
C2—C1—C6—C5	−0.5 (4)	C52—C53—C54—C55	−177.4 (3)
C2—C1—C6—C7	179.4 (2)	C53—C54—C55—C56	−0.7 (5)
C5—C6—C7—N1	162.3 (2)	C54—C55—C56—C57	−1.0 (5)
C1—C6—C7—N1	−17.5 (3)	C55—C56—C57—C58	1.7 (5)
C6—C7—N1—C8	−72.3 (3)	C54—C53—C58—C57	−0.8 (4)
C6—C7—N1—C9	89.5 (2)	C52—C53—C58—C57	178.1 (3)
C9—N1—C8—N2	2.0 (2)	C56—C57—C58—C53	−0.8 (5)
C7—N1—C8—N2	165.95 (18)	C64—C59—C60—C61	0.2 (3)
C9—N1—C8—Ag1	−169.00 (15)	C59—C60—C61—C62	0.5 (3)
C7—N1—C8—Ag1	−5.1 (3)	C60—C61—C62—C63	−0.6 (3)
C8—N1—C9—C16	−1.1 (2)	C61—C62—C63—C64	−0.1 (3)
C7—N1—C9—C16	−164.48 (19)	C60—C59—C64—C63	−0.8 (3)
C8—N1—C9—C10	−179.9 (2)	C60—C59—C64—C65	176.41 (19)
C7—N1—C9—C10	16.8 (3)	C62—C63—C64—C59	0.8 (3)
C16—C9—C10—C11	−111.4 (3)	C62—C63—C64—C65	−176.6 (2)
N1—C9—C10—C11	67.0 (3)	C59—C64—C65—N5	23.7 (3)
C16—C9—C10—C15	66.5 (4)	C63—C64—C65—N5	−158.98 (18)
N1—C9—C10—C15	−115.1 (3)	C64—C65—N5—C66	74.7 (2)
C15—C10—C11—C12	3.5 (4)	C64—C65—N5—C67	−123.2 (2)
C9—C10—C11—C12	−178.5 (2)	C67—N5—C66—N6	−0.3 (2)
C10—C11—C12—C13	−3.1 (5)	C65—N5—C66—N6	164.03 (16)
C11—C12—C13—C14	0.3 (6)	C67—N5—C66—Ag2	171.15 (13)
C12—C13—C14—C15	2.0 (7)	C65—N5—C66—Ag2	−24.5 (2)
C13—C14—C15—C10	−1.5 (7)	C66—N5—C67—C74	0.4 (2)
C11—C10—C15—C14	−1.3 (5)	C65—N5—C67—C74	−163.23 (17)
C9—C10—C15—C14	−179.3 (4)	C66—N5—C67—C68	−178.55 (17)
N1—C9—C16—N2	−0.3 (2)	C65—N5—C67—C68	17.8 (3)
C10—C9—C16—N2	178.3 (2)	C74—C67—C68—C73	43.9 (3)
N1—C9—C16—C17	177.8 (2)	N5—C67—C68—C73	−137.5 (2)
C10—C9—C16—C17	−3.7 (4)	C74—C67—C68—C69	−135.4 (2)
C9—C16—C17—C22	−69.5 (4)	N5—C67—C68—C69	43.3 (3)
N2—C16—C17—C22	108.2 (3)	C73—C68—C69—C70	1.0 (3)
C9—C16—C17—C18	113.1 (3)	C67—C68—C69—C70	−179.74 (19)
N2—C16—C17—C18	−69.2 (3)	C68—C69—C70—C71	−0.8 (3)
C22—C17—C18—C19	1.6 (4)	C69—C70—C71—C72	0.4 (3)
C16—C17—C18—C19	179.0 (2)	C70—C71—C72—C73	−0.1 (3)
C17—C18—C19—C20	−0.4 (4)	C71—C72—C73—C68	0.3 (3)
C18—C19—C20—C21	−1.6 (5)	C69—C68—C73—C72	−0.8 (3)
C19—C20—C21—C22	2.5 (6)	C67—C68—C73—C72	180.00 (19)
C18—C17—C22—C21	−0.7 (5)	N5—C67—C74—N6	−0.3 (2)
C16—C17—C22—C21	−178.1 (3)	C68—C67—C74—N6	178.52 (19)
C20—C21—C22—C17	−1.3 (6)	N5—C67—C74—C75	175.57 (19)
N1—C8—N2—C16	−2.2 (2)	C68—C67—C74—C75	−5.6 (4)
Ag1—C8—N2—C16	168.31 (16)	C67—C74—C75—C76	68.8 (3)
N1—C8—N2—C23	−173.7 (2)	N6—C74—C75—C76	−115.9 (2)
Ag1—C8—N2—C23	−3.2 (3)	C67—C74—C75—C80	−112.0 (2)
C9—C16—N2—C8	1.6 (3)	N6—C74—C75—C80	63.3 (3)
C17—C16—N2—C8	−176.6 (2)	C80—C75—C76—C77	−1.3 (3)
C9—C16—N2—C23	173.0 (2)	C74—C75—C76—C77	177.84 (19)
C17—C16—N2—C23	−5.2 (3)	C75—C76—C77—C78	0.3 (3)
C8—N2—C23—C24	106.1 (2)	C76—C77—C78—C79	0.8 (3)
C16—N2—C23—C24	−64.3 (3)	C77—C78—C79—C80	−1.0 (3)
N2—C23—C24—C25	126.8 (2)	C78—C79—C80—C75	0.0 (3)
N2—C23—C24—C29	−52.5 (3)	C76—C75—C80—C79	1.1 (3)
C29—C24—C25—C26	1.2 (4)	C74—C75—C80—C79	−178.06 (19)
C23—C24—C25—C26	−178.0 (3)	N5—C66—N6—C74	0.1 (2)
C24—C25—C26—C27	0.6 (5)	Ag2—C66—N6—C74	−170.99 (13)
C25—C26—C27—C28	−1.3 (6)	N5—C66—N6—C81	177.12 (16)
C26—C27—C28—C29	0.2 (6)	Ag2—C66—N6—C81	6.0 (3)
C27—C28—C29—C24	1.6 (5)	C67—C74—N6—C66	0.1 (2)
C25—C24—C29—C28	−2.3 (4)	C75—C74—N6—C66	−176.22 (17)
C23—C24—C29—C28	176.9 (3)	C67—C74—N6—C81	−176.79 (17)
C35—C30—C31—C32	−0.4 (5)	C75—C74—N6—C81	6.9 (3)
C30—C31—C32—C33	−0.8 (6)	C66—N6—C81—C82	−93.6 (2)
C31—C32—C33—C34	0.5 (5)	C74—N6—C81—C82	83.0 (2)
C32—C33—C34—C35	1.1 (5)	N6—C81—C82—C83	−94.5 (2)
C31—C30—C35—C34	2.0 (4)	N6—C81—C82—C87	85.3 (2)
C31—C30—C35—C36	−176.5 (3)	C87—C82—C83—C84	−1.7 (3)
C33—C34—C35—C30	−2.3 (4)	C81—C82—C83—C84	178.2 (2)
C33—C34—C35—C36	176.2 (3)	C82—C83—C84—C85	0.2 (4)
C30—C35—C36—N3	−52.4 (3)	C83—C84—C85—C86	1.2 (4)
C34—C35—C36—N3	129.1 (2)	C84—C85—C86—C87	−1.2 (4)
C35—C36—N3—C37	−65.3 (3)	C85—C86—C87—C82	−0.2 (4)
C35—C36—N3—C38	111.4 (2)	C83—C82—C87—C86	1.7 (3)
C38—N3—C37—N4	1.0 (2)	C81—C82—C87—C86	−178.1 (2)
C36—N3—C37—N4	178.19 (18)	C93—C88—C89—C90	0.7 (4)
C38—N3—C37—Ag1	−173.48 (14)	C88—C89—C90—C91	−0.5 (5)
C36—N3—C37—Ag1	3.7 (3)	C89—C90—C91—C92	0.4 (5)
C37—N3—C38—C45	−0.6 (2)	C90—C91—C92—C93	−0.4 (5)
C36—N3—C38—C45	−177.58 (19)	C89—C88—C93—C92	−0.7 (4)
C37—N3—C38—C39	−179.36 (19)	C89—C88—C93—C94	175.1 (2)
C36—N3—C38—C39	3.6 (3)	C91—C92—C93—C88	0.5 (4)
C45—C38—C39—C44	59.2 (3)	C91—C92—C93—C94	−175.4 (3)
N3—C38—C39—C44	−122.3 (2)	C88—C93—C94—N7	38.4 (3)
C45—C38—C39—C40	−119.5 (3)	C92—C93—C94—N7	−145.8 (2)
N3—C38—C39—C40	59.0 (3)	C93—C94—N7—C95	−91.0 (2)
C44—C39—C40—C41	0.8 (4)	C93—C94—N7—C96	87.2 (2)
C38—C39—C40—C41	179.6 (2)	C96—N7—C95—N8	−1.4 (2)
C39—C40—C41—C42	−0.1 (4)	C94—N7—C95—N8	177.10 (16)
C40—C41—C42—C43	−0.6 (4)	C96—N7—C95—Ag2	167.96 (14)
C41—C42—C43—C44	0.6 (5)	C94—N7—C95—Ag2	−13.6 (3)
C42—C43—C44—C39	0.1 (5)	C95—N7—C96—C103	1.5 (2)
C40—C39—C44—C43	−0.8 (4)	C94—N7—C96—C103	−176.87 (18)
C38—C39—C44—C43	−179.5 (2)	C95—N7—C96—C97	−169.15 (19)
N3—C38—C45—N4	−0.1 (2)	C94—N7—C96—C97	12.5 (3)
C39—C38—C45—N4	178.6 (2)	C103—C96—C97—C98	137.9 (2)
N3—C38—C45—C46	−175.4 (2)	N7—C96—C97—C98	−53.6 (3)
C39—C38—C45—C46	3.3 (4)	C103—C96—C97—C102	−43.7 (3)
C38—C45—C46—C51A	−82.0 (7)	N7—C96—C97—C102	124.9 (2)
N4—C45—C46—C51A	103.5 (7)	C102—C97—C98—C99	−2.2 (3)
C38—C45—C46—C47A	96.2 (7)	C96—C97—C98—C99	176.3 (2)
N4—C45—C46—C47A	−78.3 (7)	C97—C98—C99—C100	1.5 (4)
C38—C45—C46—C47B	63.6 (8)	C98—C99—C100—C101	0.6 (4)
N4—C45—C46—C47B	−110.8 (7)	C99—C100—C101—C102	−2.0 (4)
C38—C45—C46—C51B	−110.3 (8)	C98—C97—C102—C101	0.7 (3)
N4—C45—C46—C51B	75.2 (8)	C96—C97—C102—C101	−177.8 (2)
C51A—C46—C47A—C48A	1.6 (9)	C100—C101—C102—C97	1.3 (4)
C47B—C46—C47A—C48A	−69.1 (10)	N7—C96—C103—N8	−1.0 (2)
C51B—C46—C47A—C48A	28.6 (9)	C97—C96—C103—N8	169.28 (19)
C45—C46—C47A—C48A	−176.6 (5)	N7—C96—C103—C104	−178.8 (2)
C46—C47A—C48A—C49	0.2 (10)	C97—C96—C103—C104	−8.5 (4)
C51A—C46—C47B—C48B	−26.2 (9)	C96—C103—C104—C109	−76.7 (3)
C47A—C46—C47B—C48B	95.3 (12)	N8—C103—C104—C109	105.8 (3)
C51B—C46—C47B—C48B	−1.8 (10)	C96—C103—C104—C105	99.7 (3)
C45—C46—C47B—C48B	−176.0 (6)	N8—C103—C104—C105	−77.8 (3)
C46—C47B—C48B—C49	−2.2 (12)	C109—C104—C105—C106	0.8 (3)
C47B—C48B—C49—C50A	30.1 (10)	C103—C104—C105—C106	−175.7 (2)
C47B—C48B—C49—C50B	5.1 (11)	C104—C105—C106—C107	−0.6 (4)
C47B—C48B—C49—C48A	−77.0 (11)	C105—C106—C107—C108	0.0 (4)
C47A—C48A—C49—C48B	84.6 (11)	C106—C107—C108—C109	0.5 (5)
C47A—C48A—C49—C50A	−2.2 (9)	C107—C108—C109—C104	−0.3 (5)
C47A—C48A—C49—C50B	−28.6 (9)	C105—C104—C109—C108	−0.3 (4)
C48B—C49—C50A—C51A	−29.5 (10)	C103—C104—C109—C108	176.1 (2)
C50B—C49—C50A—C51A	89 (2)	N7—C95—N8—C103	0.7 (2)
C48A—C49—C50A—C51A	2.3 (10)	Ag2—C95—N8—C103	−168.99 (14)
C47A—C46—C51A—C50A	−1.5 (9)	N7—C95—N8—C110	−176.58 (17)
C47B—C46—C51A—C50A	27.0 (9)	Ag2—C95—N8—C110	13.7 (3)
C51B—C46—C51A—C50A	−88.1 (18)	C96—C103—N8—C95	0.2 (2)
C45—C46—C51A—C50A	176.6 (6)	C104—C103—N8—C95	178.16 (19)
C49—C50A—C51A—C46	−0.6 (11)	C96—C103—N8—C110	177.40 (18)
C48B—C49—C50B—C51B	−3.8 (12)	C104—C103—N8—C110	−4.6 (3)
C50A—C49—C50B—C51B	−77.2 (19)	C95—N8—C110—C111	82.2 (2)
C48A—C49—C50B—C51B	28.1 (10)	C103—N8—C110—C111	−94.7 (2)
C49—C50B—C51B—C46	−0.4 (13)	N8—C110—C111—C116	16.9 (3)
C51A—C46—C51B—C50B	77.3 (16)	N8—C110—C111—C112	−165.55 (19)
C47A—C46—C51B—C50B	−29.3 (10)	C116—C111—C112—C113	1.7 (3)
C47B—C46—C51B—C50B	3.0 (11)	C110—C111—C112—C113	−175.9 (2)
C45—C46—C51B—C50B	177.2 (7)	C111—C112—C113—C114	−0.9 (4)
N3—C37—N4—C45	−1.1 (2)	C112—C113—C114—C115	−0.7 (4)
Ag1—C37—N4—C45	173.29 (15)	C113—C114—C115—C116	1.4 (4)
N3—C37—N4—C52	177.28 (18)	C114—C115—C116—C111	−0.6 (4)
Ag1—C37—N4—C52	−8.4 (3)	C112—C111—C116—C115	−0.9 (3)
C38—C45—N4—C37	0.8 (2)	C110—C111—C116—C115	176.6 (2)
C46—C45—N4—C37	176.21 (19)	C120—C121—O1—C122	−177.1 (3)
C38—C45—N4—C52	−177.54 (19)	C121—O1—C122—C123	177.5 (3)

### Bis[bis(1,3-dibenzyl-4,5-diphenylimidazol-2-ylidene)silver(I)] tris(thiocyanato)argentate(I) diethyl ether disolvate (1) Hydrogen-bond geometry (Å, º)

**Table:**

*D*—H···*A*	*D*—H	H···*A*	*D*···*A*	*D*—H···*A*
C31—H31···O1	0.95	2.57	3.361 (4)	141
C32—H32···N10^i^	0.95	2.43	3.327 (4)	157
C40—H40···S1^ii^	0.95	2.82	3.684 (2)	152

Symmetry codes: (i) −*x*+2, −*y*+2, −*z*+1; (ii) *x*, *y*, *z*−1.

### Bis(µ-1,3-dibenzyl-4,5-diphenyl-2-selenoimidazole-κ2Se:Se)bis[bromido(1,3-dibenzyl-4,5-diphenyl-2-selenoimidazole-κSe)silver(I)] dichloromethane hexasolvate (2) Crystal data

**Table:**

[Ag_2_Br_2_(C_29_H_24_N_2_Se)_4_]·6CH_2_Cl_2_	*Z* = 1
*M**_r_* = 2802.96	*F*(000) = 1400
Triclinic, *P*1	*D*_x_ = 1.589 Mg m^−^^3^
*a* = 13.6265 (1) Å	Mo *K*α radiation, λ = 0.71073 Å
*b* = 14.7422 (1) Å	Cell parameters from 42526 reflections
*c* = 16.9397 (2) Å	θ = 3.0–26.4°
α = 106.4172 (7)°	µ = 2.58 mm^−^^1^
β = 112.2820 (8)°	*T* = 150 K
γ = 96.2211 (6)°	Prism, clear pale yellow
*V* = 2930.04 (5) Å^3^	0.37 × 0.26 × 0.20 mm

### Bis(µ-1,3-dibenzyl-4,5-diphenyl-2-selenoimidazole-κ2Se:Se)bis[bromido(1,3-dibenzyl-4,5-diphenyl-2-selenoimidazole-κSe)silver(I)] dichloromethane hexasolvate (2) Data collection

**Table:**

Rigaku SuperNova, Dual, Cu at zero, Atlas diffractometer	12002 independent reflections
Radiation source: micro-focus sealed X-ray tube	10726 reflections with *I* > 2σ(*I*)
Detector resolution: 10.3196 pixels mm^-1^	*R*_int_ = 0.037
ω scans	θ_max_ = 26.4°, θ_min_ = 2.8°
Absorption correction: gaussian (CrysAlis PRO; Rigaku OD, 2015)	*h* = −17→17
*T*_min_ = 0.517, *T*_max_ = 0.664	*k* = −18→18
119415 measured reflections	*l* = −21→21

### Bis(µ-1,3-dibenzyl-4,5-diphenyl-2-selenoimidazole-κ2Se:Se)bis[bromido(1,3-dibenzyl-4,5-diphenyl-2-selenoimidazole-κSe)silver(I)] dichloromethane hexasolvate (2) Refinement

**Table:**

Refinement on *F*^2^	Primary atom site location: structure-invariant direct methods
Least-squares matrix: full	Secondary atom site location: difference Fourier map
*R*[*F*^2^ > 2σ(*F*^2^)] = 0.035	Hydrogen site location: inferred from neighbouring sites
*wR*(*F*^2^) = 0.096	H-atom parameters constrained
*S* = 1.03	*w* = 1/[σ^2^(*F*_o_^2^) + (0.0468*P*)^2^ + 5.4618*P*] where *P* = (*F*_o_^2^ + 2*F*_c_^2^)/3
12002 reflections	(Δ/σ)_max_ = 0.002
654 parameters	Δρ_max_ = 1.05 e Å^−^^3^
0 restraints	Δρ_min_ = −1.48 e Å^−^^3^

### Bis(µ-1,3-dibenzyl-4,5-diphenyl-2-selenoimidazole-κ2Se:Se)bis[bromido(1,3-dibenzyl-4,5-diphenyl-2-selenoimidazole-κSe)silver(I)] dichloromethane hexasolvate (2) Special details

**Table:**

Geometry. All esds (except the esd in the dihedral angle between two l.s. planes) are estimated using the full covariance matrix. The cell esds are taken into account individually in the estimation of esds in distances, angles and torsion angles; correlations between esds in cell parameters are only used when they are defined by crystal symmetry. An approximate (isotropic) treatment of cell esds is used for estimating esds involving l.s. planes.
Refinement. The PLATON SQUEEZE procedure (A. L. Spek. Acta Cryst. C71, 2015, 9-18) was used to treat regions of disordered solvent which could not be modelled in terms of atomic sites. The number of electrons found in these regions, 84, was assigned to 2 molecules of dichloromethane. 2 dichloromethanes would give 84 electrons.

### Bis(µ-1,3-dibenzyl-4,5-diphenyl-2-selenoimidazole-κ2Se:Se)bis[bromido(1,3-dibenzyl-4,5-diphenyl-2-selenoimidazole-κSe)silver(I)] dichloromethane hexasolvate (2) Fractional atomic coordinates and isotropic or equivalent isotropic displacement parameters (Å^2^)

**Table:**

	*x*	*y*	*z*	*U*_iso_*/*U*_eq_	Occ. (<1)
C1	0.2991 (3)	0.2585 (2)	0.5958 (2)	0.0303 (6)	
H1	0.3686	0.2453	0.6224	0.036*	
C2	0.2264 (3)	0.2008 (3)	0.5078 (2)	0.0390 (8)	
H2	0.2460	0.1483	0.4743	0.047*	
C3	0.1255 (3)	0.2194 (3)	0.4687 (3)	0.0476 (9)	
H3	0.0752	0.1796	0.4083	0.057*	
C4	0.0978 (3)	0.2950 (3)	0.5167 (3)	0.0530 (10)	
H4	0.0283	0.3078	0.4894	0.064*	
C5	0.1709 (3)	0.3540 (3)	0.6060 (3)	0.0422 (8)	
H5	0.1509	0.4065	0.6390	0.051*	
C6	0.2721 (2)	0.3356 (2)	0.6459 (2)	0.0281 (6)	
C7	0.3529 (2)	0.3973 (2)	0.7424 (2)	0.0277 (6)	
H7A	0.3201	0.4477	0.7680	0.033*	
H7B	0.3677	0.3555	0.7801	0.033*	
N1	0.45650 (19)	0.44492 (16)	0.74753 (15)	0.0228 (5)	
C8	0.5529 (2)	0.42207 (19)	0.78510 (18)	0.0227 (5)	
Se1	0.57786 (3)	0.32825 (2)	0.83916 (2)	0.02863 (8)	
C9	0.4711 (2)	0.52088 (19)	0.71564 (18)	0.0232 (5)	
C10	0.3796 (2)	0.5572 (2)	0.66477 (19)	0.0246 (6)	
C11	0.3552 (3)	0.5471 (2)	0.5752 (2)	0.0351 (7)	
H11	0.3998	0.5197	0.5481	0.042*	
C12	0.2666 (3)	0.5764 (3)	0.5246 (2)	0.0444 (9)	
H12	0.2507	0.5691	0.4632	0.053*	
C13	0.2019 (3)	0.6158 (3)	0.5630 (3)	0.0462 (9)	
H13	0.1414	0.6362	0.5283	0.055*	
C14	0.2241 (4)	0.6259 (3)	0.6511 (3)	0.0519 (10)	
H14	0.1781	0.6521	0.6771	0.062*	
C15	0.3137 (3)	0.5981 (3)	0.7029 (2)	0.0412 (8)	
H15	0.3300	0.6070	0.7647	0.049*	
C16	0.5788 (2)	0.54519 (19)	0.73583 (18)	0.0227 (5)	
C17	0.6406 (2)	0.6262 (2)	0.72556 (19)	0.0252 (6)	
C18	0.6984 (3)	0.6095 (2)	0.6728 (2)	0.0329 (7)	
H18	0.6978	0.5446	0.6418	0.039*	
C19	0.7568 (3)	0.6868 (3)	0.6651 (3)	0.0418 (8)	
H19	0.7959	0.6749	0.6288	0.050*	
C20	0.7583 (3)	0.7811 (3)	0.7102 (3)	0.0441 (8)	
H20	0.7991	0.8341	0.7055	0.053*	
C21	0.7003 (3)	0.7986 (2)	0.7623 (3)	0.0436 (8)	
H21	0.7011	0.8638	0.7929	0.052*	
C22	0.6411 (3)	0.7217 (2)	0.7702 (2)	0.0331 (7)	
H22	0.6011	0.7340	0.8058	0.040*	
N2	0.62857 (19)	0.48262 (16)	0.77814 (15)	0.0220 (5)	
C23	0.7469 (2)	0.4912 (2)	0.82079 (19)	0.0267 (6)	
H23A	0.7644	0.4699	0.8739	0.032*	
H23B	0.7852	0.5608	0.8439	0.032*	
C24	0.7915 (2)	0.4329 (2)	0.75860 (19)	0.0273 (6)	
C25	0.7432 (3)	0.3347 (2)	0.7062 (2)	0.0311 (6)	
H25	0.6773	0.3042	0.7050	0.037*	
C26	0.7911 (3)	0.2812 (3)	0.6557 (2)	0.0387 (7)	
H26	0.7580	0.2142	0.6206	0.046*	
C27	0.8863 (3)	0.3247 (3)	0.6561 (2)	0.0393 (8)	
H27	0.9187	0.2875	0.6216	0.047*	
C28	0.9342 (3)	0.4220 (3)	0.7065 (2)	0.0413 (8)	
H28	0.9996	0.4524	0.7069	0.050*	
C29	0.8862 (3)	0.4759 (3)	0.7571 (2)	0.0351 (7)	
H29	0.9190	0.5432	0.7912	0.042*	
C30	0.5585 (3)	0.1771 (3)	1.0909 (2)	0.0491 (9)	
H30	0.6202	0.2240	1.1018	0.059*	
C31	0.5587 (4)	0.1424 (4)	1.1588 (3)	0.0648 (13)	
H31	0.6199	0.1666	1.2167	0.078*	
C32	0.4709 (4)	0.0730 (3)	1.1427 (3)	0.0563 (12)	
H32	0.4721	0.0474	1.1885	0.068*	
C33	0.3819 (4)	0.0412 (3)	1.0607 (3)	0.0651 (13)	
H33	0.3199	−0.0052	1.0498	0.078*	
C34	0.3818 (4)	0.0765 (3)	0.9930 (3)	0.0558 (11)	
H34	0.3197	0.0531	0.9356	0.067*	
C35	0.4684 (3)	0.1437 (2)	1.00705 (19)	0.0283 (6)	
C36	0.4722 (2)	0.1824 (2)	0.93410 (18)	0.0236 (5)	
H36A	0.5324	0.2419	0.9626	0.028*	
H36B	0.4878	0.1331	0.8900	0.028*	
N3	0.36874 (18)	0.20544 (16)	0.88532 (14)	0.0195 (4)	
C37	0.3351 (2)	0.28258 (19)	0.92241 (17)	0.0208 (5)	
Se2	0.41926 (2)	0.38665 (2)	1.03116 (2)	0.02432 (7)	
C38	0.2820 (2)	0.14190 (19)	0.80476 (17)	0.0208 (5)	
C39	0.2888 (2)	0.0524 (2)	0.74254 (18)	0.0222 (5)	
C40	0.3653 (2)	0.0541 (2)	0.7071 (2)	0.0280 (6)	
H40	0.4178	0.1129	0.7258	0.034*	
C41	0.3649 (3)	−0.0308 (2)	0.6440 (2)	0.0344 (7)	
H41	0.4175	−0.0297	0.6199	0.041*	
C42	0.2887 (3)	−0.1161 (2)	0.6165 (2)	0.0348 (7)	
H42	0.2887	−0.1736	0.5733	0.042*	
C43	0.2124 (3)	−0.1183 (2)	0.6515 (2)	0.0315 (7)	
H43	0.1601	−0.1773	0.6325	0.038*	
C44	0.2119 (2)	−0.0345 (2)	0.71439 (19)	0.0261 (6)	
H44	0.1592	−0.0362	0.7383	0.031*	
C45	0.1938 (2)	0.18073 (19)	0.79410 (18)	0.0217 (5)	
C46	0.0815 (2)	0.14143 (19)	0.72006 (18)	0.0224 (5)	
C47	0.0640 (2)	0.1324 (2)	0.63145 (19)	0.0273 (6)	
H47	0.1229	0.1550	0.6194	0.033*	
C48	−0.0397 (3)	0.0901 (2)	0.5609 (2)	0.0336 (7)	
H48	−0.0521	0.0847	0.5004	0.040*	
C49	−0.1246 (3)	0.0560 (2)	0.5781 (2)	0.0359 (7)	
H49	−0.1954	0.0268	0.5294	0.043*	
C50	−0.1075 (3)	0.0638 (2)	0.6657 (2)	0.0358 (7)	
H50	−0.1664	0.0395	0.6769	0.043*	
C51	−0.0047 (2)	0.1073 (2)	0.7372 (2)	0.0301 (6)	
H51	0.0068	0.1136	0.7976	0.036*	
N4	0.22814 (18)	0.26809 (16)	0.86718 (15)	0.0210 (4)	
C52	0.1591 (2)	0.3352 (2)	0.87987 (19)	0.0236 (5)	
H52A	0.1031	0.3288	0.8193	0.028*	
H52B	0.2048	0.4028	0.9088	0.028*	
C53	0.1023 (2)	0.3184 (2)	0.93731 (19)	0.0262 (6)	
C54	0.1310 (3)	0.2618 (2)	0.9917 (2)	0.0325 (7)	
H54	0.1874	0.2290	0.9922	0.039*	
C55	0.0770 (3)	0.2531 (3)	1.0453 (2)	0.0434 (8)	
H55	0.0962	0.2139	1.0820	0.052*	
C56	−0.0046 (3)	0.3012 (3)	1.0454 (3)	0.0464 (9)	
H56	−0.0407	0.2956	1.0827	0.056*	
C57	−0.0336 (3)	0.3573 (3)	0.9915 (3)	0.0463 (9)	
H57	−0.0898	0.3903	0.9914	0.056*	
C58	0.0191 (3)	0.3656 (3)	0.9375 (2)	0.0375 (7)	
H58	−0.0017	0.4040	0.9001	0.045*	
Ag	0.59867 (2)	0.44437 (2)	1.00177 (2)	0.03306 (7)	
Br	0.77487 (3)	0.41077 (3)	1.11754 (2)	0.04318 (9)	
C59	0.4858 (4)	0.7506 (4)	0.5306 (3)	0.0671 (13)	
H59A	0.5389	0.7634	0.5941	0.081*	
H59B	0.4115	0.7398	0.5281	0.081*	
Cl1	0.49731 (8)	0.64544 (7)	0.45851 (6)	0.0459 (2)	
Cl2	0.50991 (12)	0.85328 (9)	0.50091 (11)	0.0817 (4)	
C60	0.7655 (4)	0.1514 (3)	0.8195 (3)	0.0628 (12)	
H60A	0.7439	0.1961	0.8623	0.075*	0.898 (4)
H60B	0.8157	0.1914	0.8058	0.075*	0.898 (4)
H60C	0.7912	0.2167	0.8192	0.075*	0.102 (4)
H60D	0.8253	0.1173	0.8271	0.075*	0.102 (4)
Cl3	0.64844 (9)	0.08367 (10)	0.71812 (10)	0.0764 (4)	
Cl4A	0.83340 (11)	0.07387 (9)	0.87093 (9)	0.0670 (5)	0.898 (4)
Cl4B	0.7203 (18)	0.1617 (16)	0.9129 (15)	0.120 (7)*	0.102 (4)

### Bis(µ-1,3-dibenzyl-4,5-diphenyl-2-selenoimidazole-κ2Se:Se)bis[bromido(1,3-dibenzyl-4,5-diphenyl-2-selenoimidazole-κSe)silver(I)] dichloromethane hexasolvate (2) Atomic displacement parameters (Å^2^)

**Table:**

	*U*^11^	*U*^22^	*U*^33^	*U*^12^	*U*^13^	*U*^23^
C1	0.0294 (15)	0.0298 (15)	0.0306 (15)	0.0035 (12)	0.0132 (13)	0.0099 (12)
C2	0.0415 (19)	0.0386 (18)	0.0322 (17)	−0.0023 (14)	0.0181 (15)	0.0073 (14)
C3	0.041 (2)	0.055 (2)	0.0329 (18)	−0.0089 (17)	0.0050 (16)	0.0178 (17)
C4	0.0272 (18)	0.057 (2)	0.064 (3)	0.0042 (16)	0.0014 (17)	0.032 (2)
C5	0.0316 (17)	0.0375 (18)	0.056 (2)	0.0100 (14)	0.0151 (16)	0.0194 (16)
C6	0.0261 (15)	0.0273 (14)	0.0339 (16)	0.0040 (11)	0.0143 (13)	0.0138 (12)
C7	0.0294 (15)	0.0283 (14)	0.0292 (15)	0.0058 (12)	0.0173 (13)	0.0094 (12)
N1	0.0287 (12)	0.0201 (11)	0.0191 (11)	0.0052 (9)	0.0111 (10)	0.0054 (9)
C8	0.0306 (15)	0.0183 (12)	0.0171 (12)	0.0058 (11)	0.0103 (11)	0.0030 (10)
Se1	0.04242 (18)	0.02315 (14)	0.02676 (15)	0.01194 (12)	0.01801 (13)	0.01214 (12)
C9	0.0297 (15)	0.0211 (13)	0.0175 (12)	0.0055 (11)	0.0101 (11)	0.0053 (10)
C10	0.0269 (14)	0.0212 (13)	0.0252 (14)	0.0067 (11)	0.0115 (12)	0.0068 (11)
C11	0.0392 (18)	0.0455 (18)	0.0283 (16)	0.0242 (15)	0.0173 (14)	0.0147 (14)
C12	0.048 (2)	0.054 (2)	0.0320 (17)	0.0304 (18)	0.0127 (16)	0.0164 (16)
C13	0.041 (2)	0.047 (2)	0.057 (2)	0.0287 (17)	0.0187 (18)	0.0240 (18)
C14	0.061 (2)	0.057 (2)	0.068 (3)	0.042 (2)	0.045 (2)	0.032 (2)
C15	0.059 (2)	0.0440 (19)	0.0388 (18)	0.0285 (17)	0.0317 (17)	0.0190 (15)
C16	0.0283 (14)	0.0220 (13)	0.0169 (12)	0.0072 (11)	0.0090 (11)	0.0060 (10)
C17	0.0267 (14)	0.0259 (14)	0.0211 (13)	0.0065 (11)	0.0067 (11)	0.0107 (11)
C18	0.0386 (17)	0.0327 (16)	0.0321 (16)	0.0095 (13)	0.0182 (14)	0.0134 (13)
C19	0.044 (2)	0.047 (2)	0.048 (2)	0.0112 (16)	0.0277 (17)	0.0258 (17)
C20	0.0414 (19)	0.0384 (18)	0.056 (2)	0.0012 (15)	0.0201 (17)	0.0258 (17)
C21	0.051 (2)	0.0247 (16)	0.050 (2)	0.0044 (14)	0.0191 (18)	0.0113 (15)
C22	0.0392 (17)	0.0256 (15)	0.0346 (17)	0.0069 (13)	0.0166 (14)	0.0102 (13)
N2	0.0263 (12)	0.0204 (11)	0.0182 (11)	0.0069 (9)	0.0085 (9)	0.0063 (9)
C23	0.0255 (14)	0.0277 (14)	0.0185 (13)	0.0042 (11)	0.0031 (11)	0.0059 (11)
C24	0.0247 (14)	0.0347 (15)	0.0221 (14)	0.0121 (12)	0.0058 (11)	0.0133 (12)
C25	0.0315 (16)	0.0305 (15)	0.0330 (16)	0.0108 (12)	0.0140 (13)	0.0120 (13)
C26	0.0450 (19)	0.0378 (17)	0.0352 (17)	0.0201 (15)	0.0166 (15)	0.0127 (14)
C27	0.0399 (18)	0.054 (2)	0.0351 (17)	0.0282 (16)	0.0193 (15)	0.0210 (16)
C28	0.0298 (17)	0.062 (2)	0.0378 (18)	0.0155 (16)	0.0146 (15)	0.0229 (17)
C29	0.0288 (16)	0.0428 (18)	0.0293 (16)	0.0059 (13)	0.0086 (13)	0.0128 (14)
C30	0.038 (2)	0.080 (3)	0.0338 (18)	0.0264 (19)	0.0095 (16)	0.0301 (19)
C31	0.064 (3)	0.106 (4)	0.040 (2)	0.045 (3)	0.017 (2)	0.046 (2)
C32	0.104 (4)	0.054 (2)	0.041 (2)	0.054 (3)	0.041 (2)	0.0347 (19)
C33	0.096 (4)	0.051 (2)	0.046 (2)	−0.004 (2)	0.030 (2)	0.024 (2)
C34	0.070 (3)	0.050 (2)	0.0279 (18)	−0.0117 (19)	0.0063 (18)	0.0170 (16)
C35	0.0396 (17)	0.0255 (14)	0.0216 (14)	0.0170 (12)	0.0115 (13)	0.0096 (11)
C36	0.0210 (13)	0.0240 (13)	0.0201 (13)	0.0064 (10)	0.0026 (11)	0.0080 (11)
N3	0.0195 (11)	0.0202 (11)	0.0158 (10)	0.0044 (8)	0.0049 (9)	0.0060 (9)
C37	0.0234 (13)	0.0202 (12)	0.0154 (12)	0.0034 (10)	0.0061 (10)	0.0054 (10)
Se2	0.02608 (15)	0.02286 (14)	0.01577 (13)	0.00262 (11)	0.00472 (11)	0.00219 (10)
C38	0.0199 (13)	0.0222 (13)	0.0154 (12)	0.0030 (10)	0.0035 (10)	0.0062 (10)
C39	0.0215 (13)	0.0240 (13)	0.0156 (12)	0.0093 (10)	0.0020 (10)	0.0062 (10)
C40	0.0247 (14)	0.0323 (15)	0.0244 (14)	0.0096 (12)	0.0077 (12)	0.0093 (12)
C41	0.0331 (16)	0.0427 (18)	0.0276 (15)	0.0167 (14)	0.0142 (13)	0.0084 (13)
C42	0.0388 (17)	0.0315 (16)	0.0228 (15)	0.0169 (13)	0.0053 (13)	0.0012 (12)
C43	0.0307 (16)	0.0229 (14)	0.0270 (15)	0.0073 (12)	0.0012 (12)	0.0041 (12)
C44	0.0258 (14)	0.0253 (14)	0.0221 (13)	0.0068 (11)	0.0053 (11)	0.0080 (11)
C45	0.0233 (13)	0.0209 (13)	0.0164 (12)	0.0044 (10)	0.0055 (11)	0.0049 (10)
C46	0.0202 (13)	0.0210 (13)	0.0200 (13)	0.0074 (10)	0.0036 (11)	0.0050 (10)
C47	0.0294 (15)	0.0276 (14)	0.0213 (14)	0.0084 (12)	0.0071 (12)	0.0082 (11)
C48	0.0373 (17)	0.0317 (16)	0.0198 (14)	0.0104 (13)	0.0013 (13)	0.0064 (12)
C49	0.0260 (15)	0.0278 (15)	0.0321 (16)	0.0048 (12)	−0.0040 (13)	0.0031 (13)
C50	0.0234 (15)	0.0359 (17)	0.0383 (18)	0.0030 (12)	0.0079 (13)	0.0083 (14)
C51	0.0265 (15)	0.0343 (16)	0.0253 (15)	0.0063 (12)	0.0093 (12)	0.0076 (12)
N4	0.0216 (11)	0.0202 (11)	0.0175 (11)	0.0054 (9)	0.0059 (9)	0.0046 (9)
C52	0.0249 (14)	0.0214 (13)	0.0221 (13)	0.0094 (11)	0.0080 (11)	0.0057 (11)
C53	0.0246 (14)	0.0258 (14)	0.0197 (13)	0.0034 (11)	0.0065 (11)	0.0014 (11)
C54	0.0337 (16)	0.0324 (16)	0.0297 (16)	0.0061 (13)	0.0127 (13)	0.0107 (13)
C55	0.047 (2)	0.045 (2)	0.0382 (19)	0.0003 (16)	0.0190 (16)	0.0175 (16)
C56	0.042 (2)	0.051 (2)	0.0402 (19)	−0.0052 (16)	0.0238 (17)	0.0052 (16)
C57	0.0378 (19)	0.050 (2)	0.048 (2)	0.0116 (16)	0.0242 (17)	0.0057 (17)
C58	0.0346 (17)	0.0434 (19)	0.0366 (18)	0.0158 (14)	0.0170 (15)	0.0125 (15)
Ag	0.03885 (14)	0.03004 (12)	0.02210 (12)	0.00221 (10)	0.00806 (10)	0.00678 (9)
Br	0.03466 (18)	0.0598 (2)	0.03813 (19)	0.01376 (16)	0.01402 (15)	0.02296 (16)
C59	0.078 (3)	0.079 (3)	0.044 (2)	0.035 (3)	0.031 (2)	0.008 (2)
Cl1	0.0467 (5)	0.0523 (5)	0.0417 (5)	0.0182 (4)	0.0221 (4)	0.0143 (4)
Cl2	0.0795 (8)	0.0507 (6)	0.1100 (11)	0.0188 (6)	0.0553 (8)	0.0001 (6)
C60	0.056 (3)	0.046 (2)	0.070 (3)	0.0032 (19)	0.023 (2)	0.006 (2)
Cl3	0.0393 (6)	0.0724 (8)	0.0863 (9)	0.0018 (5)	0.0126 (6)	0.0080 (6)
Cl4A	0.0709 (9)	0.0580 (8)	0.0583 (8)	−0.0081 (6)	0.0150 (6)	0.0272 (6)

### Bis(µ-1,3-dibenzyl-4,5-diphenyl-2-selenoimidazole-κ2Se:Se)bis[bromido(1,3-dibenzyl-4,5-diphenyl-2-selenoimidazole-κSe)silver(I)] dichloromethane hexasolvate (2) Geometric parameters (Å, º)

**Table:**

C1—C2	1.381 (5)	C33—C34	1.387 (5)
C1—C6	1.390 (4)	C33—H33	0.9500
C1—H1	0.9500	C34—C35	1.352 (5)
C2—C3	1.379 (5)	C34—H34	0.9500
C2—H2	0.9500	C35—C36	1.515 (4)
C3—C4	1.362 (6)	C36—N3	1.471 (3)
C3—H3	0.9500	C36—H36A	0.9900
C4—C5	1.400 (6)	C36—H36B	0.9900
C4—H4	0.9500	N3—C37	1.351 (3)
C5—C6	1.383 (5)	N3—C38	1.397 (3)
C5—H5	0.9500	C37—N4	1.353 (3)
C6—C7	1.512 (4)	C37—Se2	1.866 (3)
C7—N1	1.466 (4)	Se2—Ag^i^	2.7187 (4)
C7—H7A	0.9900	Se2—Ag	2.7677 (4)
C7—H7B	0.9900	C38—C45	1.359 (4)
N1—C8	1.352 (4)	C38—C39	1.475 (4)
N1—C9	1.400 (3)	C39—C40	1.386 (4)
C8—N2	1.351 (4)	C39—C44	1.397 (4)
C8—Se1	1.857 (3)	C40—C41	1.395 (4)
Se1—Ag	2.6899 (4)	C40—H40	0.9500
C9—C16	1.354 (4)	C41—C42	1.377 (5)
C9—C10	1.475 (4)	C41—H41	0.9500
C10—C11	1.384 (4)	C42—C43	1.380 (5)
C10—C15	1.386 (4)	C42—H42	0.9500
C11—C12	1.384 (4)	C43—C44	1.387 (4)
C11—H11	0.9500	C43—H43	0.9500
C12—C13	1.368 (5)	C44—H44	0.9500
C12—H12	0.9500	C45—N4	1.392 (3)
C13—C14	1.365 (6)	C45—C46	1.481 (4)
C13—H13	0.9500	C46—C51	1.389 (4)
C14—C15	1.389 (5)	C46—C47	1.392 (4)
C14—H14	0.9500	C47—C48	1.387 (4)
C15—H15	0.9500	C47—H47	0.9500
C16—N2	1.402 (3)	C48—C49	1.374 (5)
C16—C17	1.476 (4)	C48—H48	0.9500
C17—C18	1.389 (4)	C49—C50	1.380 (5)
C17—C22	1.396 (4)	C49—H49	0.9500
C18—C19	1.384 (5)	C50—C51	1.386 (4)
C18—H18	0.9500	C50—H50	0.9500
C19—C20	1.377 (5)	C51—H51	0.9500
C19—H19	0.9500	N4—C52	1.465 (3)
C20—C21	1.384 (5)	C52—C53	1.507 (4)
C20—H20	0.9500	C52—H52A	0.9900
C21—C22	1.387 (5)	C52—H52B	0.9900
C21—H21	0.9500	C53—C54	1.389 (4)
C22—H22	0.9500	C53—C58	1.395 (4)
N2—C23	1.469 (4)	C54—C55	1.392 (5)
C23—C24	1.518 (4)	C54—H54	0.9500
C23—H23A	0.9900	C55—C56	1.383 (6)
C23—H23B	0.9900	C55—H55	0.9500
C24—C29	1.387 (4)	C56—C57	1.378 (6)
C24—C25	1.393 (4)	C56—H56	0.9500
C25—C26	1.391 (5)	C57—C58	1.381 (5)
C25—H25	0.9500	C57—H57	0.9500
C26—C27	1.381 (5)	C58—H58	0.9500
C26—H26	0.9500	Ag—Br	2.6631 (4)
C27—C28	1.377 (5)	Ag—Se2^i^	2.7186 (4)
C27—H27	0.9500	C59—Cl1	1.746 (4)
C28—C29	1.395 (5)	C59—Cl2	1.761 (6)
C28—H28	0.9500	C59—H59A	0.9900
C29—H29	0.9500	C59—H59B	0.9900
C30—C31	1.384 (6)	C60—Cl3	1.760 (5)
C30—C35	1.385 (5)	C60—Cl4A	1.762 (5)
C30—H30	0.9500	C60—Cl4B	1.88 (2)
C31—C32	1.372 (7)	C60—H60A	0.9900
C31—H31	0.9500	C60—H60B	0.9900
C32—C33	1.360 (7)	C60—H60C	0.9900
C32—H32	0.9500	C60—H60D	0.9900
			
C2—C1—C6	120.9 (3)	C35—C34—H34	119.4
C2—C1—H1	119.5	C33—C34—H34	119.4
C6—C1—H1	119.5	C34—C35—C30	118.8 (3)
C3—C2—C1	119.9 (3)	C34—C35—C36	122.7 (3)
C3—C2—H2	120.0	C30—C35—C36	118.4 (3)
C1—C2—H2	120.0	N3—C36—C35	112.0 (2)
C4—C3—C2	119.9 (3)	N3—C36—H36A	109.2
C4—C3—H3	120.0	C35—C36—H36A	109.2
C2—C3—H3	120.0	N3—C36—H36B	109.2
C3—C4—C5	120.7 (3)	C35—C36—H36B	109.2
C3—C4—H4	119.7	H36A—C36—H36B	107.9
C5—C4—H4	119.7	C37—N3—C38	109.6 (2)
C6—C5—C4	119.8 (4)	C37—N3—C36	123.6 (2)
C6—C5—H5	120.1	C38—N3—C36	125.3 (2)
C4—C5—H5	120.1	N3—C37—N4	106.8 (2)
C5—C6—C1	118.7 (3)	N3—C37—Se2	126.4 (2)
C5—C6—C7	121.4 (3)	N4—C37—Se2	126.8 (2)
C1—C6—C7	119.8 (3)	C37—Se2—Ag^i^	108.43 (8)
N1—C7—C6	112.7 (2)	C37—Se2—Ag	100.72 (8)
N1—C7—H7A	109.1	Ag^i^—Se2—Ag	73.649 (11)
C6—C7—H7A	109.1	C45—C38—N3	106.9 (2)
N1—C7—H7B	109.1	C45—C38—C39	127.7 (2)
C6—C7—H7B	109.1	N3—C38—C39	125.4 (2)
H7A—C7—H7B	107.8	C40—C39—C44	119.5 (3)
C8—N1—C9	109.8 (2)	C40—C39—C38	121.4 (3)
C8—N1—C7	124.5 (2)	C44—C39—C38	118.9 (3)
C9—N1—C7	125.7 (2)	C39—C40—C41	119.9 (3)
N2—C8—N1	106.8 (2)	C39—C40—H40	120.1
N2—C8—Se1	126.4 (2)	C41—C40—H40	120.1
N1—C8—Se1	126.8 (2)	C42—C41—C40	120.3 (3)
C8—Se1—Ag	94.72 (8)	C42—C41—H41	119.9
C16—C9—N1	106.7 (2)	C40—C41—H41	119.9
C16—C9—C10	130.3 (3)	C41—C42—C43	120.2 (3)
N1—C9—C10	122.8 (3)	C41—C42—H42	119.9
C11—C10—C15	118.5 (3)	C43—C42—H42	119.9
C11—C10—C9	119.6 (3)	C42—C43—C44	120.2 (3)
C15—C10—C9	121.9 (3)	C42—C43—H43	119.9
C10—C11—C12	120.7 (3)	C44—C43—H43	119.9
C10—C11—H11	119.6	C43—C44—C39	120.0 (3)
C12—C11—H11	119.6	C43—C44—H44	120.0
C13—C12—C11	120.1 (3)	C39—C44—H44	120.0
C13—C12—H12	120.0	C38—C45—N4	106.9 (2)
C11—C12—H12	120.0	C38—C45—C46	128.4 (2)
C14—C13—C12	120.2 (3)	N4—C45—C46	124.7 (2)
C14—C13—H13	119.9	C51—C46—C47	119.9 (3)
C12—C13—H13	119.9	C51—C46—C45	120.6 (3)
C13—C14—C15	120.2 (3)	C47—C46—C45	119.3 (3)
C13—C14—H14	119.9	C48—C47—C46	119.7 (3)
C15—C14—H14	119.9	C48—C47—H47	120.2
C10—C15—C14	120.3 (3)	C46—C47—H47	120.2
C10—C15—H15	119.8	C49—C48—C47	120.2 (3)
C14—C15—H15	119.8	C49—C48—H48	119.9
C9—C16—N2	107.2 (2)	C47—C48—H48	119.9
C9—C16—C17	129.5 (3)	C48—C49—C50	120.4 (3)
N2—C16—C17	123.1 (2)	C48—C49—H49	119.8
C18—C17—C22	119.4 (3)	C50—C49—H49	119.8
C18—C17—C16	121.6 (3)	C49—C50—C51	120.2 (3)
C22—C17—C16	119.0 (3)	C49—C50—H50	119.9
C19—C18—C17	120.5 (3)	C51—C50—H50	119.9
C19—C18—H18	119.8	C50—C51—C46	119.6 (3)
C17—C18—H18	119.8	C50—C51—H51	120.2
C20—C19—C18	120.0 (3)	C46—C51—H51	120.2
C20—C19—H19	120.0	C37—N4—C45	109.7 (2)
C18—C19—H19	120.0	C37—N4—C52	125.6 (2)
C19—C20—C21	120.1 (3)	C45—N4—C52	124.7 (2)
C19—C20—H20	120.0	N4—C52—C53	114.1 (2)
C21—C20—H20	120.0	N4—C52—H52A	108.7
C20—C21—C22	120.4 (3)	C53—C52—H52A	108.7
C20—C21—H21	119.8	N4—C52—H52B	108.7
C22—C21—H21	119.8	C53—C52—H52B	108.7
C21—C22—C17	119.6 (3)	H52A—C52—H52B	107.6
C21—C22—H22	120.2	C54—C53—C58	118.9 (3)
C17—C22—H22	120.2	C54—C53—C52	123.7 (3)
C8—N2—C16	109.5 (2)	C58—C53—C52	117.3 (3)
C8—N2—C23	124.8 (2)	C53—C54—C55	119.9 (3)
C16—N2—C23	125.1 (2)	C53—C54—H54	120.0
N2—C23—C24	115.0 (2)	C55—C54—H54	120.0
N2—C23—H23A	108.5	C56—C55—C54	120.4 (3)
C24—C23—H23A	108.5	C56—C55—H55	119.8
N2—C23—H23B	108.5	C54—C55—H55	119.8
C24—C23—H23B	108.5	C57—C56—C55	120.0 (3)
H23A—C23—H23B	107.5	C57—C56—H56	120.0
C29—C24—C25	118.5 (3)	C55—C56—H56	120.0
C29—C24—C23	119.8 (3)	C56—C57—C58	120.0 (3)
C25—C24—C23	121.6 (3)	C56—C57—H57	120.0
C26—C25—C24	120.2 (3)	C58—C57—H57	120.0
C26—C25—H25	119.9	C57—C58—C53	120.8 (3)
C24—C25—H25	119.9	C57—C58—H58	119.6
C27—C26—C25	120.7 (3)	C53—C58—H58	119.6
C27—C26—H26	119.7	Br—Ag—Se1	102.274 (13)
C25—C26—H26	119.7	Br—Ag—Se2^i^	126.883 (14)
C28—C27—C26	119.8 (3)	Se1—Ag—Se2^i^	100.026 (11)
C28—C27—H27	120.1	Br—Ag—Se2	109.628 (12)
C26—C27—H27	120.1	Se1—Ag—Se2	110.623 (12)
C27—C28—C29	119.7 (3)	Se2^i^—Ag—Se2	106.352 (11)
C27—C28—H28	120.2	Cl1—C59—Cl2	112.3 (3)
C29—C28—H28	120.2	Cl1—C59—H59A	109.1
C24—C29—C28	121.2 (3)	Cl2—C59—H59A	109.1
C24—C29—H29	119.4	Cl1—C59—H59B	109.1
C28—C29—H29	119.4	Cl2—C59—H59B	109.1
C31—C30—C35	120.1 (4)	H59A—C59—H59B	107.9
C31—C30—H30	119.9	Cl3—C60—Cl4A	110.9 (2)
C35—C30—H30	119.9	Cl3—C60—Cl4B	104.9 (7)
C32—C31—C30	120.2 (4)	Cl3—C60—H60A	109.5
C32—C31—H31	119.9	Cl4A—C60—H60A	109.5
C30—C31—H31	119.9	Cl3—C60—H60B	109.5
C33—C32—C31	119.5 (4)	Cl4A—C60—H60B	109.5
C33—C32—H32	120.2	H60A—C60—H60B	108.0
C31—C32—H32	120.2	Cl3—C60—H60C	110.8
C32—C33—C34	120.0 (4)	Cl4B—C60—H60C	110.8
C32—C33—H33	120.0	Cl3—C60—H60D	110.8
C34—C33—H33	120.0	Cl4B—C60—H60D	110.8
C35—C34—C33	121.2 (4)	H60C—C60—H60D	108.8
			
C6—C1—C2—C3	0.0 (5)	C30—C31—C32—C33	−2.3 (7)
C1—C2—C3—C4	0.3 (5)	C31—C32—C33—C34	2.0 (7)
C2—C3—C4—C5	−0.3 (6)	C32—C33—C34—C35	−1.0 (8)
C3—C4—C5—C6	0.1 (6)	C33—C34—C35—C30	0.1 (6)
C4—C5—C6—C1	0.2 (5)	C33—C34—C35—C36	178.7 (4)
C4—C5—C6—C7	−179.6 (3)	C31—C30—C35—C34	−0.4 (6)
C2—C1—C6—C5	−0.2 (5)	C31—C30—C35—C36	−179.0 (4)
C2—C1—C6—C7	179.6 (3)	C34—C35—C36—N3	44.2 (4)
C5—C6—C7—N1	−120.5 (3)	C30—C35—C36—N3	−137.3 (3)
C1—C6—C7—N1	59.7 (4)	C35—C36—N3—C37	72.1 (3)
C6—C7—N1—C8	−111.0 (3)	C35—C36—N3—C38	−92.7 (3)
C6—C7—N1—C9	69.7 (3)	C38—N3—C37—N4	0.8 (3)
C9—N1—C8—N2	0.4 (3)	C36—N3—C37—N4	−166.1 (2)
C7—N1—C8—N2	−179.0 (2)	C38—N3—C37—Se2	178.82 (19)
C9—N1—C8—Se1	178.68 (19)	C36—N3—C37—Se2	11.9 (4)
C7—N1—C8—Se1	−0.7 (4)	N3—C37—Se2—Ag^i^	128.8 (2)
N2—C8—Se1—Ag	94.9 (2)	N4—C37—Se2—Ag^i^	−53.6 (2)
N1—C8—Se1—Ag	−83.1 (2)	N3—C37—Se2—Ag	52.6 (2)
C8—N1—C9—C16	−1.0 (3)	N4—C37—Se2—Ag	−129.7 (2)
C7—N1—C9—C16	178.4 (2)	C37—N3—C38—C45	−1.2 (3)
C8—N1—C9—C10	175.2 (2)	C36—N3—C38—C45	165.5 (2)
C7—N1—C9—C10	−5.5 (4)	C37—N3—C38—C39	176.5 (3)
C16—C9—C10—C11	59.5 (4)	C36—N3—C38—C39	−16.9 (4)
N1—C9—C10—C11	−115.8 (3)	C45—C38—C39—C40	123.0 (3)
C16—C9—C10—C15	−123.2 (4)	N3—C38—C39—C40	−54.1 (4)
N1—C9—C10—C15	61.6 (4)	C45—C38—C39—C44	−52.8 (4)
C15—C10—C11—C12	−0.5 (5)	N3—C38—C39—C44	130.1 (3)
C9—C10—C11—C12	176.9 (3)	C44—C39—C40—C41	−0.1 (4)
C10—C11—C12—C13	0.0 (6)	C38—C39—C40—C41	−176.0 (3)
C11—C12—C13—C14	−0.3 (6)	C39—C40—C41—C42	0.2 (5)
C12—C13—C14—C15	1.2 (7)	C40—C41—C42—C43	−0.3 (5)
C11—C10—C15—C14	1.4 (5)	C41—C42—C43—C44	0.2 (5)
C9—C10—C15—C14	−176.0 (3)	C42—C43—C44—C39	−0.1 (4)
C13—C14—C15—C10	−1.8 (6)	C40—C39—C44—C43	0.1 (4)
N1—C9—C16—N2	1.2 (3)	C38—C39—C44—C43	176.0 (2)
C10—C9—C16—N2	−174.6 (3)	N3—C38—C45—N4	1.0 (3)
N1—C9—C16—C17	−173.6 (3)	C39—C38—C45—N4	−176.5 (3)
C10—C9—C16—C17	10.6 (5)	N3—C38—C45—C46	−179.5 (3)
C9—C16—C17—C18	−121.8 (3)	C39—C38—C45—C46	2.9 (5)
N2—C16—C17—C18	64.1 (4)	C38—C45—C46—C51	111.2 (3)
C9—C16—C17—C22	58.3 (4)	N4—C45—C46—C51	−69.5 (4)
N2—C16—C17—C22	−115.7 (3)	C38—C45—C46—C47	−64.2 (4)
C22—C17—C18—C19	0.6 (5)	N4—C45—C46—C47	115.2 (3)
C16—C17—C18—C19	−179.3 (3)	C51—C46—C47—C48	0.7 (4)
C17—C18—C19—C20	0.2 (5)	C45—C46—C47—C48	176.1 (3)
C18—C19—C20—C21	−0.8 (6)	C46—C47—C48—C49	−1.0 (4)
C19—C20—C21—C22	0.5 (6)	C47—C48—C49—C50	0.3 (5)
C20—C21—C22—C17	0.3 (5)	C48—C49—C50—C51	0.7 (5)
C18—C17—C22—C21	−0.9 (5)	C49—C50—C51—C46	−1.0 (5)
C16—C17—C22—C21	179.0 (3)	C47—C46—C51—C50	0.3 (4)
N1—C8—N2—C16	0.4 (3)	C45—C46—C51—C50	−175.0 (3)
Se1—C8—N2—C16	−177.93 (19)	N3—C37—N4—C45	−0.1 (3)
N1—C8—N2—C23	171.8 (2)	Se2—C37—N4—C45	−178.15 (19)
Se1—C8—N2—C23	−6.5 (4)	N3—C37—N4—C52	−178.2 (2)
C9—C16—N2—C8	−1.0 (3)	Se2—C37—N4—C52	3.8 (4)
C17—C16—N2—C8	174.2 (2)	C38—C45—N4—C37	−0.6 (3)
C9—C16—N2—C23	−172.4 (2)	C46—C45—N4—C37	180.0 (2)
C17—C16—N2—C23	2.8 (4)	C38—C45—N4—C52	177.5 (2)
C8—N2—C23—C24	97.6 (3)	C46—C45—N4—C52	−2.0 (4)
C16—N2—C23—C24	−92.3 (3)	C37—N4—C52—C53	−89.4 (3)
N2—C23—C24—C29	134.4 (3)	C45—N4—C52—C53	92.9 (3)
N2—C23—C24—C25	−49.4 (4)	N4—C52—C53—C54	14.8 (4)
C29—C24—C25—C26	1.5 (4)	N4—C52—C53—C58	−167.5 (3)
C23—C24—C25—C26	−174.7 (3)	C58—C53—C54—C55	−0.1 (5)
C24—C25—C26—C27	−0.6 (5)	C52—C53—C54—C55	177.5 (3)
C25—C26—C27—C28	−0.3 (5)	C53—C54—C55—C56	−0.5 (5)
C26—C27—C28—C29	0.2 (5)	C54—C55—C56—C57	0.7 (6)
C25—C24—C29—C28	−1.7 (5)	C55—C56—C57—C58	−0.1 (6)
C23—C24—C29—C28	174.7 (3)	C56—C57—C58—C53	−0.5 (5)
C27—C28—C29—C24	0.8 (5)	C54—C53—C58—C57	0.7 (5)
C35—C30—C31—C32	1.5 (7)	C52—C53—C58—C57	−177.2 (3)

Symmetry code: (i) −*x*+1, −*y*+1, −*z*+2.

### catena-Poly[[[(1,3-dibenzyl-4,5-diphenyl-2-selenoimidazole-κSe)copper(I)]-µ-cyanido-κ2C:N] acetonitrile monosolvate] (3) Crystal data

**Table:**

[Cu(CN)(C_29_H_24_N_2_Se)]·C_2_H_3_N	*F*(000) = 2480
*M**_r_* = 610.07	*D*_x_ = 1.447 Mg m^−^^3^
Monoclinic, *P*2_1_/*c*	Mo *K*α radiation, λ = 0.71073 Å
*a* = 13.7704 (3) Å	Cell parameters from 23662 reflections
*b* = 14.3398 (3) Å	θ = 3.0–26.3°
*c* = 28.4102 (7) Å	µ = 2.11 mm^−^^1^
β = 93.024 (2)°	*T* = 100 K
*V* = 5602.2 (2) Å^3^	Rod, colourless
*Z* = 8	0.35 × 0.12 × 0.11 mm

### catena-Poly[[[(1,3-dibenzyl-4,5-diphenyl-2-selenoimidazole-κSe)copper(I)]-µ-cyanido-κ2C:N] acetonitrile monosolvate] (3) Data collection

**Table:**

Rigaku SuperNova, Dual, Cu at zero, Atlas diffractometer	11392 independent reflections
Radiation source: micro-focus sealed X-ray tube	10122 reflections with *I* > 2σ(*I*)
Detector resolution: 10.3196 pixels mm^-1^	*R*_int_ = 0.038
ω scans	θ_max_ = 26.4°, θ_min_ = 2.8°
Absorption correction: gaussian (CrysAlis PRO; Rigaku OD, 2015)	*h* = −17→17
*T*_min_ = 0.643, *T*_max_ = 0.851	*k* = −17→17
71725 measured reflections	*l* = −34→35

### catena-Poly[[[(1,3-dibenzyl-4,5-diphenyl-2-selenoimidazole-κSe)copper(I)]-µ-cyanido-κ2C:N] acetonitrile monosolvate] (3) Refinement

**Table:**

Refinement on *F*^2^	Primary atom site location: structure-invariant direct methods
Least-squares matrix: full	Secondary atom site location: difference Fourier map
*R*[*F*^2^ > 2σ(*F*^2^)] = 0.048	Hydrogen site location: inferred from neighbouring sites
*wR*(*F*^2^) = 0.119	H-atom parameters constrained
*S* = 1.06	*w* = 1/[σ^2^(*F*_o_^2^) + (0.045*P*)^2^ + 17.9881*P*] where *P* = (*F*_o_^2^ + 2*F*_c_^2^)/3
11392 reflections	(Δ/σ)_max_ = 0.002
687 parameters	Δρ_max_ = 2.04 e Å^−^^3^
0 restraints	Δρ_min_ = −0.56 e Å^−^^3^

### catena-Poly[[[(1,3-dibenzyl-4,5-diphenyl-2-selenoimidazole-κSe)copper(I)]-µ-cyanido-κ2C:N] acetonitrile monosolvate] (3) Special details

**Table:**

Geometry. All esds (except the esd in the dihedral angle between two l.s. planes) are estimated using the full covariance matrix. The cell esds are taken into account individually in the estimation of esds in distances, angles and torsion angles; correlations between esds in cell parameters are only used when they are defined by crystal symmetry. An approximate (isotropic) treatment of cell esds is used for estimating esds involving l.s. planes.

### catena-Poly[[[(1,3-dibenzyl-4,5-diphenyl-2-selenoimidazole-κSe)copper(I)]-µ-cyanido-κ2C:N] acetonitrile monosolvate] (3) Fractional atomic coordinates and isotropic or equivalent isotropic displacement parameters (Å^2^)

**Table:**

	*x*	*y*	*z*	*U*_iso_*/*U*_eq_	
C1	0.7661 (3)	0.8105 (3)	0.56492 (13)	0.0266 (8)	
H1	0.8151	0.7909	0.5448	0.032*	
C2	0.6869 (3)	0.8612 (3)	0.54673 (14)	0.0324 (9)	
H2	0.6819	0.8758	0.5141	0.039*	
C3	0.6153 (3)	0.8906 (3)	0.57562 (15)	0.0332 (9)	
H3	0.5620	0.9265	0.5631	0.040*	
C4	0.6218 (3)	0.8673 (3)	0.62283 (15)	0.0343 (9)	
H4	0.5720	0.8859	0.6427	0.041*	
C5	0.7008 (3)	0.8171 (3)	0.64125 (13)	0.0253 (7)	
H5	0.7051	0.8019	0.6738	0.030*	
C6	0.7738 (2)	0.7886 (2)	0.61249 (12)	0.0189 (7)	
C7	0.8551 (2)	0.7292 (2)	0.63438 (12)	0.0204 (7)	
H7A	0.8727	0.7532	0.6664	0.024*	
H7B	0.8310	0.6646	0.6377	0.024*	
N1	0.9424 (2)	0.72729 (19)	0.60732 (9)	0.0183 (6)	
C8	0.9750 (2)	0.6511 (2)	0.58509 (11)	0.0200 (7)	
C9	1.0034 (2)	0.8028 (2)	0.60041 (11)	0.0174 (6)	
C10	0.9878 (2)	0.8956 (2)	0.62140 (11)	0.0177 (6)	
C11	0.9911 (2)	0.9067 (2)	0.67034 (12)	0.0218 (7)	
H11	1.0039	0.8545	0.6903	0.026*	
C12	0.9757 (3)	0.9937 (3)	0.68992 (13)	0.0262 (8)	
H12	0.9773	1.0007	0.7232	0.031*	
C13	0.9580 (3)	1.0703 (3)	0.66121 (14)	0.0281 (8)	
H13	0.9476	1.1298	0.6747	0.034*	
C14	0.9554 (3)	1.0602 (3)	0.61274 (14)	0.0284 (8)	
H14	0.9438	1.1130	0.5930	0.034*	
C15	0.9697 (3)	0.9730 (2)	0.59275 (12)	0.0237 (7)	
H15	0.9671	0.9663	0.5594	0.028*	
C16	1.0749 (2)	0.7722 (2)	0.57321 (11)	0.0188 (7)	
C17	1.1600 (2)	0.8218 (2)	0.55600 (11)	0.0180 (6)	
C18	1.1500 (3)	0.8798 (3)	0.51648 (13)	0.0301 (8)	
H18	1.0883	0.8867	0.5003	0.036*	
C19	1.2298 (3)	0.9274 (3)	0.50083 (15)	0.0357 (9)	
H19	1.2227	0.9667	0.4739	0.043*	
C20	1.3199 (3)	0.9176 (3)	0.52444 (14)	0.0313 (8)	
H20	1.3743	0.9508	0.5139	0.038*	
C21	1.3312 (3)	0.8598 (3)	0.56325 (13)	0.0281 (8)	
H21	1.3933	0.8525	0.5790	0.034*	
C22	1.2508 (3)	0.8122 (3)	0.57926 (12)	0.0235 (7)	
H22	1.2582	0.7730	0.6062	0.028*	
N2	1.0562 (2)	0.67822 (19)	0.56411 (9)	0.0195 (6)	
C23	1.1121 (3)	0.6179 (2)	0.53376 (12)	0.0224 (7)	
H23A	1.1495	0.6576	0.5127	0.027*	
H23B	1.0663	0.5800	0.5137	0.027*	
C24	1.1821 (3)	0.5528 (3)	0.56083 (13)	0.0253 (7)	
C25	1.2045 (3)	0.5611 (3)	0.60863 (13)	0.0269 (8)	
H25	1.1780	0.6111	0.6257	0.032*	
C26	1.2657 (3)	0.4965 (3)	0.63204 (15)	0.0384 (10)	
H26	1.2812	0.5033	0.6648	0.046*	
C27	1.3040 (3)	0.4226 (4)	0.60772 (16)	0.0453 (11)	
H27	1.3435	0.3774	0.6239	0.054*	
C28	1.2840 (3)	0.4152 (3)	0.55930 (16)	0.0464 (11)	
H28	1.3121	0.3661	0.5421	0.056*	
C29	1.2230 (3)	0.4793 (3)	0.53623 (15)	0.0390 (10)	
H29	1.2089	0.4733	0.5033	0.047*	
Se1	0.92117 (3)	0.53117 (2)	0.58409 (2)	0.02398 (10)	
Cu1	0.95522 (3)	0.49927 (3)	0.66587 (2)	0.02577 (11)	
C59	0.9042 (3)	0.3973 (3)	0.69925 (13)	0.0282 (8)	
N5	0.8749 (2)	0.3390 (2)	0.72193 (12)	0.0322 (7)	
Cu2	0.82825 (3)	0.24571 (3)	0.76454 (2)	0.02719 (12)	
Se2	0.66152 (3)	0.27327 (3)	0.77892 (2)	0.02914 (10)	
C30	0.5614 (4)	0.3668 (4)	0.59119 (16)	0.0474 (11)	
H30	0.5037	0.4033	0.5905	0.057*	
C31	0.6036 (5)	0.3434 (5)	0.54929 (18)	0.0644 (16)	
H31	0.5758	0.3649	0.5200	0.077*	
C32	0.6879 (4)	0.2878 (4)	0.55079 (18)	0.0641 (17)	
H32	0.7136	0.2659	0.5224	0.077*	
C33	0.7316 (5)	0.2661 (5)	0.5926 (2)	0.084 (2)	
H33	0.7924	0.2347	0.5937	0.101*	
C34	0.6894 (4)	0.2887 (4)	0.63443 (18)	0.0614 (16)	
H34	0.7205	0.2706	0.6636	0.074*	
C35	0.6040 (3)	0.3366 (3)	0.63423 (13)	0.0280 (8)	
C36	0.5573 (3)	0.3625 (3)	0.67936 (13)	0.0262 (8)	
H36A	0.4960	0.3965	0.6714	0.031*	
H36B	0.5404	0.3047	0.6961	0.031*	
N3	0.6190 (2)	0.4204 (2)	0.71106 (10)	0.0216 (6)	
C37	0.6612 (3)	0.3937 (2)	0.75286 (12)	0.0232 (7)	
C38	0.6364 (2)	0.5153 (2)	0.70361 (12)	0.0199 (7)	
C39	0.6004 (2)	0.5646 (2)	0.66026 (12)	0.0208 (7)	
C40	0.6554 (3)	0.5642 (3)	0.62088 (13)	0.0283 (8)	
H40	0.7164	0.5330	0.6219	0.034*	
C41	0.6218 (3)	0.6090 (3)	0.57994 (14)	0.0364 (10)	
H41	0.6600	0.6091	0.5531	0.044*	
C42	0.5336 (3)	0.6530 (3)	0.57830 (15)	0.0427 (11)	
H42	0.5104	0.6835	0.5502	0.051*	
C43	0.4786 (3)	0.6529 (4)	0.61719 (19)	0.0559 (14)	
H43	0.4170	0.6831	0.6157	0.067*	
C44	0.5114 (3)	0.6097 (3)	0.65840 (16)	0.0430 (11)	
H44	0.4732	0.6109	0.6853	0.052*	
C45	0.6898 (2)	0.5460 (2)	0.74189 (12)	0.0194 (7)	
C46	0.7299 (2)	0.6395 (2)	0.75337 (11)	0.0194 (7)	
C47	0.8295 (3)	0.6547 (2)	0.75481 (12)	0.0233 (7)	
H47	0.8723	0.6051	0.7480	0.028*	
C48	0.8669 (3)	0.7425 (3)	0.76618 (12)	0.0276 (8)	
H48	0.9352	0.7525	0.7673	0.033*	
C49	0.8050 (3)	0.8150 (3)	0.77590 (13)	0.0313 (9)	
H49	0.8305	0.8750	0.7836	0.038*	
C50	0.7054 (3)	0.7999 (3)	0.77436 (13)	0.0314 (8)	
H50	0.6629	0.8497	0.7811	0.038*	
C51	0.6676 (3)	0.7132 (3)	0.76306 (12)	0.0251 (7)	
H51	0.5993	0.7035	0.7619	0.030*	
N4	0.7045 (2)	0.4698 (2)	0.77188 (10)	0.0214 (6)	
C52	0.7539 (3)	0.4747 (2)	0.81887 (12)	0.0235 (7)	
H52A	0.8209	0.4984	0.8159	0.028*	
H52B	0.7586	0.4112	0.8325	0.028*	
C53	0.7010 (3)	0.5374 (2)	0.85174 (12)	0.0223 (7)	
C54	0.5996 (3)	0.5417 (3)	0.84997 (13)	0.0292 (8)	
H54	0.5627	0.5049	0.8277	0.035*	
C55	0.5525 (3)	0.5998 (3)	0.88072 (15)	0.0381 (10)	
H55	0.4835	0.6028	0.8793	0.046*	
C56	0.6058 (3)	0.6533 (3)	0.91339 (16)	0.0426 (10)	
H56	0.5736	0.6932	0.9342	0.051*	
C57	0.7057 (3)	0.6483 (3)	0.91558 (15)	0.0411 (10)	
H57	0.7424	0.6846	0.9381	0.049*	
C58	0.7534 (3)	0.5903 (3)	0.88486 (13)	0.0302 (8)	
H58	0.8224	0.5871	0.8867	0.036*	
C60	0.9083 (3)	0.1490 (2)	0.79035 (12)	0.0238 (7)	
N6	0.9596 (2)	0.0943 (2)	0.80625 (11)	0.0280 (7)	
N7	0.5766 (3)	0.4987 (3)	0.24282 (14)	0.0445 (9)	
C61	0.6400 (3)	0.4766 (3)	0.22229 (16)	0.0365 (9)	
C62	0.7238 (4)	0.4507 (4)	0.1964 (2)	0.0624 (16)	
H62A	0.7024	0.4155	0.1682	0.094*	
H62B	0.7677	0.4121	0.2164	0.094*	
H62C	0.7581	0.5072	0.1871	0.094*	
N8	0.9405 (3)	0.7629 (3)	0.47523 (14)	0.0484 (10)	
C63	0.9333 (3)	0.7302 (3)	0.43914 (15)	0.0340 (9)	
C64	0.9265 (4)	0.6919 (3)	0.39148 (16)	0.0476 (11)	
H64A	0.8753	0.7245	0.3727	0.071*	
H64B	0.9109	0.6253	0.3929	0.071*	
H64C	0.9888	0.7001	0.3769	0.071*	

### catena-Poly[[[(1,3-dibenzyl-4,5-diphenyl-2-selenoimidazole-κSe)copper(I)]-µ-cyanido-κ2C:N] acetonitrile monosolvate] (3) Atomic displacement parameters (Å^2^)

**Table:**

	*U*^11^	*U*^22^	*U*^33^	*U*^12^	*U*^13^	*U*^23^
C1	0.0223 (18)	0.035 (2)	0.0229 (18)	0.0024 (15)	0.0034 (14)	0.0067 (15)
C2	0.030 (2)	0.039 (2)	0.028 (2)	−0.0039 (17)	−0.0033 (16)	0.0129 (17)
C3	0.0228 (19)	0.029 (2)	0.047 (2)	0.0033 (15)	−0.0051 (17)	0.0048 (17)
C4	0.0237 (19)	0.038 (2)	0.041 (2)	0.0055 (16)	0.0071 (17)	−0.0092 (18)
C5	0.0246 (18)	0.0293 (19)	0.0222 (18)	0.0007 (14)	0.0043 (14)	−0.0037 (14)
C6	0.0183 (16)	0.0174 (15)	0.0212 (16)	−0.0028 (12)	0.0018 (13)	−0.0007 (13)
C7	0.0200 (16)	0.0244 (17)	0.0173 (16)	0.0015 (13)	0.0062 (13)	0.0041 (13)
N1	0.0178 (14)	0.0198 (14)	0.0172 (13)	0.0013 (11)	0.0015 (11)	0.0004 (11)
C8	0.0218 (17)	0.0209 (16)	0.0170 (16)	0.0032 (13)	−0.0014 (13)	0.0002 (13)
C9	0.0169 (16)	0.0216 (16)	0.0135 (15)	0.0027 (12)	−0.0005 (12)	0.0018 (12)
C10	0.0155 (15)	0.0195 (16)	0.0184 (16)	0.0019 (12)	0.0037 (12)	−0.0004 (12)
C11	0.0187 (16)	0.0263 (17)	0.0204 (17)	0.0001 (13)	0.0004 (13)	−0.0008 (14)
C12	0.0219 (18)	0.0318 (19)	0.0251 (18)	−0.0009 (14)	0.0047 (14)	−0.0084 (15)
C13	0.0225 (18)	0.0231 (18)	0.039 (2)	0.0000 (14)	0.0069 (16)	−0.0098 (16)
C14	0.0283 (19)	0.0226 (18)	0.035 (2)	0.0040 (15)	0.0062 (16)	0.0042 (15)
C15	0.0266 (18)	0.0240 (17)	0.0207 (17)	0.0031 (14)	0.0040 (14)	0.0003 (14)
C16	0.0223 (17)	0.0209 (16)	0.0131 (15)	0.0050 (13)	−0.0019 (12)	0.0016 (12)
C17	0.0193 (16)	0.0207 (16)	0.0144 (15)	0.0036 (13)	0.0035 (12)	−0.0014 (12)
C18	0.0244 (18)	0.040 (2)	0.0260 (19)	0.0019 (16)	0.0010 (15)	0.0080 (16)
C19	0.032 (2)	0.043 (2)	0.033 (2)	−0.0007 (18)	0.0044 (17)	0.0143 (18)
C20	0.0261 (19)	0.032 (2)	0.037 (2)	−0.0038 (16)	0.0085 (16)	0.0020 (17)
C21	0.0207 (18)	0.033 (2)	0.031 (2)	0.0021 (15)	0.0011 (15)	−0.0050 (16)
C22	0.0243 (18)	0.0279 (18)	0.0185 (16)	0.0024 (14)	0.0021 (14)	0.0005 (14)
N2	0.0196 (14)	0.0224 (14)	0.0165 (14)	0.0035 (11)	0.0022 (11)	−0.0019 (11)
C23	0.0254 (18)	0.0247 (17)	0.0172 (16)	0.0039 (14)	0.0035 (14)	−0.0032 (13)
C24	0.0227 (18)	0.0296 (19)	0.0235 (18)	0.0052 (14)	0.0008 (14)	−0.0017 (15)
C25	0.0266 (19)	0.0305 (19)	0.0237 (18)	0.0027 (15)	0.0015 (14)	−0.0045 (15)
C26	0.035 (2)	0.053 (3)	0.026 (2)	0.0072 (19)	−0.0058 (17)	−0.0002 (18)
C27	0.038 (2)	0.057 (3)	0.040 (2)	0.016 (2)	−0.0028 (19)	0.009 (2)
C28	0.048 (3)	0.050 (3)	0.041 (2)	0.025 (2)	0.000 (2)	−0.004 (2)
C29	0.046 (2)	0.044 (2)	0.027 (2)	0.017 (2)	−0.0013 (18)	−0.0035 (18)
Se1	0.02574 (19)	0.02198 (18)	0.02412 (19)	−0.00181 (13)	0.00024 (14)	−0.00282 (13)
Cu1	0.0309 (2)	0.0204 (2)	0.0263 (2)	−0.00198 (17)	0.00483 (18)	0.00115 (17)
C59	0.036 (2)	0.0238 (18)	0.0250 (19)	−0.0014 (15)	0.0041 (16)	−0.0006 (15)
N5	0.0349 (18)	0.0259 (17)	0.0360 (18)	0.0016 (14)	0.0032 (14)	0.0023 (14)
Cu2	0.0365 (3)	0.0186 (2)	0.0263 (2)	0.00050 (18)	0.00004 (19)	0.00181 (17)
Se2	0.0374 (2)	0.02157 (18)	0.0290 (2)	−0.00150 (15)	0.00708 (16)	0.00452 (14)
C30	0.043 (3)	0.065 (3)	0.035 (2)	−0.007 (2)	−0.001 (2)	−0.007 (2)
C31	0.072 (4)	0.090 (4)	0.031 (3)	−0.021 (3)	0.002 (2)	−0.013 (3)
C32	0.066 (4)	0.089 (4)	0.039 (3)	−0.022 (3)	0.024 (3)	−0.037 (3)
C33	0.076 (4)	0.116 (6)	0.060 (4)	0.047 (4)	0.015 (3)	−0.030 (4)
C34	0.065 (3)	0.083 (4)	0.036 (3)	0.034 (3)	0.002 (2)	−0.013 (3)
C35	0.033 (2)	0.0274 (19)	0.0243 (18)	−0.0089 (15)	0.0049 (15)	−0.0091 (15)
C36	0.0266 (19)	0.0271 (18)	0.0253 (18)	−0.0062 (15)	0.0028 (15)	−0.0051 (15)
N3	0.0247 (15)	0.0220 (14)	0.0185 (14)	−0.0006 (12)	0.0041 (12)	−0.0025 (11)
C37	0.0268 (18)	0.0210 (17)	0.0224 (17)	0.0010 (14)	0.0070 (14)	−0.0007 (14)
C38	0.0188 (16)	0.0213 (16)	0.0202 (17)	0.0006 (13)	0.0056 (13)	−0.0002 (13)
C39	0.0215 (17)	0.0225 (16)	0.0181 (16)	−0.0036 (13)	−0.0009 (13)	−0.0023 (13)
C40	0.033 (2)	0.0285 (19)	0.0243 (18)	−0.0025 (15)	0.0062 (15)	−0.0016 (15)
C41	0.054 (3)	0.036 (2)	0.0202 (19)	−0.0099 (19)	0.0053 (18)	−0.0018 (16)
C42	0.052 (3)	0.044 (2)	0.030 (2)	−0.014 (2)	−0.018 (2)	0.0143 (19)
C43	0.033 (2)	0.069 (3)	0.065 (3)	0.012 (2)	−0.003 (2)	0.035 (3)
C44	0.033 (2)	0.057 (3)	0.040 (2)	0.015 (2)	0.0140 (19)	0.020 (2)
C45	0.0185 (16)	0.0216 (16)	0.0188 (16)	0.0041 (13)	0.0068 (13)	−0.0007 (13)
C46	0.0248 (17)	0.0213 (16)	0.0123 (15)	0.0006 (13)	0.0010 (13)	0.0010 (12)
C47	0.0267 (18)	0.0257 (18)	0.0175 (16)	0.0010 (14)	0.0017 (14)	0.0015 (13)
C48	0.030 (2)	0.032 (2)	0.0205 (17)	−0.0070 (16)	0.0002 (15)	0.0021 (15)
C49	0.049 (2)	0.0245 (19)	0.0204 (18)	−0.0069 (17)	−0.0023 (16)	0.0005 (14)
C50	0.044 (2)	0.0229 (18)	0.0271 (19)	0.0058 (16)	0.0042 (17)	−0.0024 (15)
C51	0.0247 (18)	0.0271 (18)	0.0235 (18)	0.0025 (14)	0.0032 (14)	−0.0005 (14)
N4	0.0261 (15)	0.0209 (14)	0.0174 (14)	0.0020 (12)	0.0027 (11)	0.0013 (11)
C52	0.0244 (18)	0.0251 (17)	0.0209 (17)	0.0003 (14)	−0.0015 (14)	0.0051 (14)
C53	0.0267 (18)	0.0249 (17)	0.0155 (16)	−0.0027 (14)	0.0035 (13)	0.0037 (13)
C54	0.0261 (19)	0.036 (2)	0.0251 (19)	−0.0069 (16)	0.0014 (15)	−0.0022 (16)
C55	0.028 (2)	0.054 (3)	0.032 (2)	0.0029 (19)	0.0047 (17)	−0.0054 (19)
C56	0.048 (3)	0.044 (2)	0.038 (2)	−0.002 (2)	0.014 (2)	−0.0121 (19)
C57	0.046 (3)	0.046 (2)	0.032 (2)	−0.018 (2)	0.0035 (19)	−0.0149 (19)
C58	0.029 (2)	0.036 (2)	0.0264 (19)	−0.0104 (16)	0.0034 (15)	−0.0013 (16)
C60	0.0297 (19)	0.0225 (17)	0.0195 (17)	−0.0037 (15)	0.0045 (14)	0.0009 (14)
N6	0.0349 (18)	0.0267 (16)	0.0229 (16)	−0.0004 (14)	0.0078 (13)	−0.0035 (13)
N7	0.037 (2)	0.047 (2)	0.050 (2)	−0.0062 (17)	0.0076 (18)	0.0022 (18)
C61	0.031 (2)	0.037 (2)	0.042 (2)	−0.0038 (18)	−0.0033 (19)	0.0119 (18)
C62	0.044 (3)	0.064 (3)	0.081 (4)	0.023 (3)	0.020 (3)	0.030 (3)
N8	0.042 (2)	0.072 (3)	0.032 (2)	−0.003 (2)	−0.0019 (17)	−0.0028 (19)
C63	0.032 (2)	0.036 (2)	0.034 (2)	0.0025 (17)	−0.0027 (17)	0.0068 (18)
C64	0.064 (3)	0.040 (2)	0.038 (2)	0.012 (2)	0.003 (2)	−0.005 (2)

### catena-Poly[[[(1,3-dibenzyl-4,5-diphenyl-2-selenoimidazole-κSe)copper(I)]-µ-cyanido-κ2C:N] acetonitrile monosolvate] (3) Geometric parameters (Å, º)

**Table:**

C1—C6	1.386 (5)	C30—H30	0.9500
C1—C2	1.388 (5)	C31—C32	1.407 (9)
C1—H1	0.9500	C31—H31	0.9500
C2—C3	1.382 (6)	C32—C33	1.340 (9)
C2—H2	0.9500	C32—H32	0.9500
C3—C4	1.381 (6)	C33—C34	1.389 (7)
C3—H3	0.9500	C33—H33	0.9500
C4—C5	1.384 (5)	C34—C35	1.360 (6)
C4—H4	0.9500	C34—H34	0.9500
C5—C6	1.391 (5)	C35—C36	1.512 (5)
C5—H5	0.9500	C36—N3	1.464 (4)
C6—C7	1.513 (5)	C36—H36A	0.9900
C7—N1	1.461 (4)	C36—H36B	0.9900
C7—H7A	0.9900	N3—C37	1.350 (5)
C7—H7B	0.9900	N3—C38	1.399 (4)
N1—C8	1.351 (4)	C37—N4	1.343 (5)
N1—C9	1.391 (4)	C38—C45	1.354 (5)
C8—N2	1.351 (4)	C38—C39	1.483 (5)
C8—Se1	1.872 (3)	C39—C40	1.383 (5)
C9—C16	1.356 (5)	C39—C44	1.385 (5)
C9—C10	1.478 (4)	C40—C41	1.387 (6)
C10—C15	1.391 (5)	C40—H40	0.9500
C10—C11	1.398 (5)	C41—C42	1.367 (7)
C11—C12	1.386 (5)	C41—H41	0.9500
C11—H11	0.9500	C42—C43	1.372 (7)
C12—C13	1.382 (5)	C42—H42	0.9500
C12—H12	0.9500	C43—C44	1.379 (6)
C13—C14	1.383 (5)	C43—H43	0.9500
C13—H13	0.9500	C44—H44	0.9500
C14—C15	1.391 (5)	C45—N4	1.394 (4)
C14—H14	0.9500	C45—C46	1.480 (5)
C15—H15	0.9500	C46—C47	1.387 (5)
C16—N2	1.393 (4)	C46—C51	1.397 (5)
C16—C17	1.478 (5)	C47—C48	1.391 (5)
C17—C22	1.389 (5)	C47—H47	0.9500
C17—C18	1.398 (5)	C48—C49	1.382 (6)
C18—C19	1.386 (6)	C48—H48	0.9500
C18—H18	0.9500	C49—C50	1.386 (6)
C19—C20	1.386 (6)	C49—H49	0.9500
C19—H19	0.9500	C50—C51	1.380 (5)
C20—C21	1.382 (6)	C50—H50	0.9500
C20—H20	0.9500	C51—H51	0.9500
C21—C22	1.398 (5)	N4—C52	1.468 (4)
C21—H21	0.9500	C52—C53	1.512 (5)
C22—H22	0.9500	C52—H52A	0.9900
N2—C23	1.468 (4)	C52—H52B	0.9900
C23—C24	1.521 (5)	C53—C58	1.382 (5)
C23—H23A	0.9900	C53—C54	1.395 (5)
C23—H23B	0.9900	C54—C55	1.392 (6)
C24—C25	1.382 (5)	C54—H54	0.9500
C24—C29	1.399 (5)	C55—C56	1.384 (6)
C25—C26	1.397 (6)	C55—H55	0.9500
C25—H25	0.9500	C56—C57	1.375 (6)
C26—C27	1.384 (6)	C56—H56	0.9500
C26—H26	0.9500	C57—C58	1.395 (6)
C27—C28	1.393 (6)	C57—H57	0.9500
C27—H27	0.9500	C58—H58	0.9500
C28—C29	1.386 (6)	C60—N6	1.134 (5)
C28—H28	0.9500	N6—Cu1^ii^	1.939 (3)
C29—H29	0.9500	N7—C61	1.121 (6)
Se1—Cu1	2.3900 (6)	C61—C62	1.450 (7)
Cu1—C59	1.898 (4)	C62—H62A	0.9800
Cu1—N6^i^	1.939 (3)	C62—H62B	0.9800
C59—N5	1.141 (5)	C62—H62C	0.9800
N5—Cu2	1.937 (3)	N8—C63	1.127 (6)
Cu2—C60	1.895 (4)	C63—C64	1.460 (6)
Cu2—Se2	2.3861 (6)	C64—H64A	0.9800
Se2—C37	1.879 (3)	C64—H64B	0.9800
C30—C31	1.393 (7)	C64—H64C	0.9800
C30—C35	1.397 (6)		
			
C6—C1—C2	120.0 (3)	C30—C31—C32	119.4 (5)
C6—C1—H1	120.0	C30—C31—H31	120.3
C2—C1—H1	120.0	C32—C31—H31	120.3
C3—C2—C1	120.7 (4)	C33—C32—C31	119.3 (5)
C3—C2—H2	119.7	C33—C32—H32	120.3
C1—C2—H2	119.7	C31—C32—H32	120.3
C4—C3—C2	119.4 (4)	C32—C33—C34	121.0 (6)
C4—C3—H3	120.3	C32—C33—H33	119.5
C2—C3—H3	120.3	C34—C33—H33	119.5
C3—C4—C5	120.2 (4)	C35—C34—C33	121.0 (5)
C3—C4—H4	119.9	C35—C34—H34	119.5
C5—C4—H4	119.9	C33—C34—H34	119.5
C4—C5—C6	120.6 (3)	C34—C35—C30	118.9 (4)
C4—C5—H5	119.7	C34—C35—C36	121.8 (4)
C6—C5—H5	119.7	C30—C35—C36	119.2 (4)
C1—C6—C5	119.1 (3)	N3—C36—C35	113.7 (3)
C1—C6—C7	123.1 (3)	N3—C36—H36A	108.8
C5—C6—C7	117.8 (3)	C35—C36—H36A	108.8
N1—C7—C6	113.9 (3)	N3—C36—H36B	108.8
N1—C7—H7A	108.8	C35—C36—H36B	108.8
C6—C7—H7A	108.8	H36A—C36—H36B	107.7
N1—C7—H7B	108.8	C37—N3—C38	109.9 (3)
C6—C7—H7B	108.8	C37—N3—C36	126.1 (3)
H7A—C7—H7B	107.7	C38—N3—C36	123.8 (3)
C8—N1—C9	110.2 (3)	N4—C37—N3	106.5 (3)
C8—N1—C7	124.4 (3)	N4—C37—Se2	126.6 (3)
C9—N1—C7	125.4 (3)	N3—C37—Se2	126.9 (3)
N2—C8—N1	106.0 (3)	C45—C38—N3	106.6 (3)
N2—C8—Se1	126.5 (2)	C45—C38—C39	130.9 (3)
N1—C8—Se1	127.5 (3)	N3—C38—C39	122.5 (3)
C16—C9—N1	106.8 (3)	C40—C39—C44	119.5 (3)
C16—C9—C10	130.3 (3)	C40—C39—C38	119.8 (3)
N1—C9—C10	122.9 (3)	C44—C39—C38	120.7 (3)
C15—C10—C11	119.1 (3)	C39—C40—C41	120.2 (4)
C15—C10—C9	120.5 (3)	C39—C40—H40	119.9
C11—C10—C9	120.4 (3)	C41—C40—H40	119.9
C12—C11—C10	120.3 (3)	C42—C41—C40	120.0 (4)
C12—C11—H11	119.9	C42—C41—H41	120.0
C10—C11—H11	119.9	C40—C41—H41	120.0
C13—C12—C11	120.3 (3)	C41—C42—C43	120.0 (4)
C13—C12—H12	119.9	C41—C42—H42	120.0
C11—C12—H12	119.9	C43—C42—H42	120.0
C12—C13—C14	119.9 (3)	C42—C43—C44	120.9 (4)
C12—C13—H13	120.0	C42—C43—H43	119.6
C14—C13—H13	120.0	C44—C43—H43	119.6
C13—C14—C15	120.3 (3)	C43—C44—C39	119.5 (4)
C13—C14—H14	119.9	C43—C44—H44	120.3
C15—C14—H14	119.9	C39—C44—H44	120.3
C10—C15—C14	120.2 (3)	C38—C45—N4	106.9 (3)
C10—C15—H15	119.9	C38—C45—C46	130.8 (3)
C14—C15—H15	119.9	N4—C45—C46	122.4 (3)
C9—C16—N2	106.7 (3)	C47—C46—C51	119.4 (3)
C9—C16—C17	130.1 (3)	C47—C46—C45	120.4 (3)
N2—C16—C17	123.1 (3)	C51—C46—C45	120.1 (3)
C22—C17—C18	119.3 (3)	C46—C47—C48	120.2 (3)
C22—C17—C16	120.3 (3)	C46—C47—H47	119.9
C18—C17—C16	120.4 (3)	C48—C47—H47	119.9
C19—C18—C17	120.3 (3)	C49—C48—C47	120.2 (4)
C19—C18—H18	119.9	C49—C48—H48	119.9
C17—C18—H18	119.9	C47—C48—H48	119.9
C18—C19—C20	119.9 (4)	C48—C49—C50	119.7 (4)
C18—C19—H19	120.0	C48—C49—H49	120.2
C20—C19—H19	120.0	C50—C49—H49	120.2
C21—C20—C19	120.5 (4)	C51—C50—C49	120.6 (4)
C21—C20—H20	119.8	C51—C50—H50	119.7
C19—C20—H20	119.8	C49—C50—H50	119.7
C20—C21—C22	119.7 (3)	C50—C51—C46	119.9 (3)
C20—C21—H21	120.2	C50—C51—H51	120.0
C22—C21—H21	120.2	C46—C51—H51	120.0
C17—C22—C21	120.3 (3)	C37—N4—C45	110.1 (3)
C17—C22—H22	119.8	C37—N4—C52	125.4 (3)
C21—C22—H22	119.8	C45—N4—C52	124.3 (3)
C8—N2—C16	110.2 (3)	N4—C52—C53	112.0 (3)
C8—N2—C23	124.1 (3)	N4—C52—H52A	109.2
C16—N2—C23	125.5 (3)	C53—C52—H52A	109.2
N2—C23—C24	113.8 (3)	N4—C52—H52B	109.2
N2—C23—H23A	108.8	C53—C52—H52B	109.2
C24—C23—H23A	108.8	H52A—C52—H52B	107.9
N2—C23—H23B	108.8	C58—C53—C54	119.1 (3)
C24—C23—H23B	108.8	C58—C53—C52	119.7 (3)
H23A—C23—H23B	107.7	C54—C53—C52	121.2 (3)
C25—C24—C29	118.7 (3)	C55—C54—C53	120.2 (4)
C25—C24—C23	123.1 (3)	C55—C54—H54	119.9
C29—C24—C23	118.1 (3)	C53—C54—H54	119.9
C24—C25—C26	120.6 (4)	C56—C55—C54	120.3 (4)
C24—C25—H25	119.7	C56—C55—H55	119.9
C26—C25—H25	119.7	C54—C55—H55	119.9
C27—C26—C25	120.4 (4)	C57—C56—C55	119.7 (4)
C27—C26—H26	119.8	C57—C56—H56	120.2
C25—C26—H26	119.8	C55—C56—H56	120.2
C26—C27—C28	119.4 (4)	C56—C57—C58	120.4 (4)
C26—C27—H27	120.3	C56—C57—H57	119.8
C28—C27—H27	120.3	C58—C57—H57	119.8
C29—C28—C27	119.9 (4)	C53—C58—C57	120.4 (4)
C29—C28—H28	120.0	C53—C58—H58	119.8
C27—C28—H28	120.0	C57—C58—H58	119.8
C28—C29—C24	120.9 (4)	N6—C60—Cu2	176.7 (3)
C28—C29—H29	119.6	C60—N6—Cu1^ii^	178.6 (3)
C24—C29—H29	119.6	N7—C61—C62	178.0 (5)
C8—Se1—Cu1	95.96 (10)	C61—C62—H62A	109.5
C59—Cu1—N6^i^	124.68 (14)	C61—C62—H62B	109.5
C59—Cu1—Se1	125.06 (12)	H62A—C62—H62B	109.5
N6^i^—Cu1—Se1	110.25 (9)	C61—C62—H62C	109.5
N5—C59—Cu1	175.6 (3)	H62A—C62—H62C	109.5
C59—N5—Cu2	175.7 (3)	H62B—C62—H62C	109.5
C60—Cu2—N5	122.84 (15)	N8—C63—C64	177.2 (5)
C60—Cu2—Se2	126.88 (11)	C63—C64—H64A	109.5
N5—Cu2—Se2	110.28 (10)	C63—C64—H64B	109.5
C37—Se2—Cu2	93.84 (11)	H64A—C64—H64B	109.5
C31—C30—C35	119.8 (5)	C63—C64—H64C	109.5
C31—C30—H30	120.1	H64A—C64—H64C	109.5
C35—C30—H30	120.1	H64B—C64—H64C	109.5
			
C6—C1—C2—C3	0.3 (6)	C35—C30—C31—C32	1.3 (8)
C1—C2—C3—C4	−1.5 (6)	C30—C31—C32—C33	−6.6 (9)
C2—C3—C4—C5	1.7 (6)	C31—C32—C33—C34	7.2 (11)
C3—C4—C5—C6	−0.7 (6)	C32—C33—C34—C35	−2.3 (11)
C2—C1—C6—C5	0.8 (5)	C33—C34—C35—C30	−3.1 (9)
C2—C1—C6—C7	176.6 (3)	C33—C34—C35—C36	−179.5 (6)
C4—C5—C6—C1	−0.5 (5)	C31—C30—C35—C34	3.5 (7)
C4—C5—C6—C7	−176.6 (3)	C31—C30—C35—C36	−180.0 (4)
C1—C6—C7—N1	22.7 (5)	C34—C35—C36—N3	58.9 (6)
C5—C6—C7—N1	−161.4 (3)	C30—C35—C36—N3	−117.5 (4)
C6—C7—N1—C8	−112.7 (3)	C35—C36—N3—C37	−110.2 (4)
C6—C7—N1—C9	66.9 (4)	C35—C36—N3—C38	75.0 (4)
C9—N1—C8—N2	−0.1 (4)	C38—N3—C37—N4	0.2 (4)
C7—N1—C8—N2	179.5 (3)	C36—N3—C37—N4	−175.2 (3)
C9—N1—C8—Se1	178.8 (2)	C38—N3—C37—Se2	−178.8 (2)
C7—N1—C8—Se1	−1.5 (5)	C36—N3—C37—Se2	5.8 (5)
C8—N1—C9—C16	0.2 (4)	Cu2—Se2—C37—N4	−72.0 (3)
C7—N1—C9—C16	−179.5 (3)	Cu2—Se2—C37—N3	106.7 (3)
C8—N1—C9—C10	−178.5 (3)	C37—N3—C38—C45	−0.3 (4)
C7—N1—C9—C10	1.8 (5)	C36—N3—C38—C45	175.2 (3)
C16—C9—C10—C15	63.8 (5)	C37—N3—C38—C39	179.0 (3)
N1—C9—C10—C15	−117.8 (4)	C36—N3—C38—C39	−5.5 (5)
C16—C9—C10—C11	−116.2 (4)	C45—C38—C39—C40	92.5 (5)
N1—C9—C10—C11	62.2 (4)	N3—C38—C39—C40	−86.6 (4)
C15—C10—C11—C12	0.5 (5)	C45—C38—C39—C44	−88.8 (5)
C9—C10—C11—C12	−179.5 (3)	N3—C38—C39—C44	92.1 (5)
C10—C11—C12—C13	−0.7 (5)	C44—C39—C40—C41	0.4 (6)
C11—C12—C13—C14	0.1 (5)	C38—C39—C40—C41	179.1 (3)
C12—C13—C14—C15	0.6 (6)	C39—C40—C41—C42	−0.7 (6)
C11—C10—C15—C14	0.2 (5)	C40—C41—C42—C43	0.2 (7)
C9—C10—C15—C14	−179.8 (3)	C41—C42—C43—C44	0.6 (8)
C13—C14—C15—C10	−0.7 (6)	C42—C43—C44—C39	−0.9 (8)
N1—C9—C16—N2	−0.1 (3)	C40—C39—C44—C43	0.4 (7)
C10—C9—C16—N2	178.4 (3)	C38—C39—C44—C43	−178.3 (4)
N1—C9—C16—C17	−178.4 (3)	N3—C38—C45—N4	0.3 (4)
C10—C9—C16—C17	0.1 (6)	C39—C38—C45—N4	−178.9 (3)
C9—C16—C17—C22	98.5 (4)	N3—C38—C45—C46	179.5 (3)
N2—C16—C17—C22	−79.5 (4)	C39—C38—C45—C46	0.3 (6)
C9—C16—C17—C18	−80.8 (5)	C38—C45—C46—C47	−111.4 (4)
N2—C16—C17—C18	101.2 (4)	N4—C45—C46—C47	67.7 (4)
C22—C17—C18—C19	−0.2 (6)	C38—C45—C46—C51	68.9 (5)
C16—C17—C18—C19	179.1 (4)	N4—C45—C46—C51	−112.1 (4)
C17—C18—C19—C20	0.0 (6)	C51—C46—C47—C48	0.5 (5)
C18—C19—C20—C21	0.7 (6)	C45—C46—C47—C48	−179.2 (3)
C19—C20—C21—C22	−1.1 (6)	C46—C47—C48—C49	−0.3 (5)
C18—C17—C22—C21	−0.1 (5)	C47—C48—C49—C50	0.1 (5)
C16—C17—C22—C21	−179.4 (3)	C48—C49—C50—C51	−0.2 (6)
C20—C21—C22—C17	0.8 (5)	C49—C50—C51—C46	0.4 (6)
N1—C8—N2—C16	0.0 (4)	C47—C46—C51—C50	−0.6 (5)
Se1—C8—N2—C16	−178.9 (2)	C45—C46—C51—C50	179.1 (3)
N1—C8—N2—C23	−176.4 (3)	N3—C37—N4—C45	0.0 (4)
Se1—C8—N2—C23	4.6 (5)	Se2—C37—N4—C45	178.9 (2)
C9—C16—N2—C8	0.0 (4)	N3—C37—N4—C52	175.7 (3)
C17—C16—N2—C8	178.5 (3)	Se2—C37—N4—C52	−5.3 (5)
C9—C16—N2—C23	176.4 (3)	C38—C45—N4—C37	−0.1 (4)
C17—C16—N2—C23	−5.1 (5)	C46—C45—N4—C37	−179.4 (3)
C8—N2—C23—C24	−81.7 (4)	C38—C45—N4—C52	−175.9 (3)
C16—N2—C23—C24	102.4 (4)	C46—C45—N4—C52	4.8 (5)
N2—C23—C24—C25	−11.3 (5)	C37—N4—C52—C53	−113.1 (4)
N2—C23—C24—C29	166.4 (4)	C45—N4—C52—C53	62.0 (4)
C29—C24—C25—C26	−0.9 (6)	N4—C52—C53—C58	−145.1 (3)
C23—C24—C25—C26	176.9 (4)	N4—C52—C53—C54	36.2 (5)
C24—C25—C26—C27	−0.8 (7)	C58—C53—C54—C55	1.1 (6)
C25—C26—C27—C28	2.5 (7)	C52—C53—C54—C55	179.8 (4)
C26—C27—C28—C29	−2.6 (8)	C53—C54—C55—C56	−0.3 (6)
C27—C28—C29—C24	1.0 (8)	C54—C55—C56—C57	−0.4 (7)
C25—C24—C29—C28	0.8 (7)	C55—C56—C57—C58	0.5 (7)
C23—C24—C29—C28	−177.1 (4)	C54—C53—C58—C57	−1.0 (6)
N2—C8—Se1—Cu1	111.9 (3)	C52—C53—C58—C57	−179.8 (4)
N1—C8—Se1—Cu1	−66.9 (3)	C56—C57—C58—C53	0.3 (7)

Symmetry codes: (i) −*x*+2, *y*+1/2, −*z*+3/2; (ii) −*x*+2, *y*−1/2, −*z*+3/2.
